# Material Analysis and a Visual Guide of Degradation Phenomena in Historical Synthetic Polymers as Tools to Follow Ageing Processes in Industrial Heritage Collections

**DOI:** 10.3390/polym14010121

**Published:** 2021-12-29

**Authors:** Till Krieg, Cristian Mazzon, Elena Gómez-Sánchez

**Affiliations:** Material Science Department, Deutsches Bergbau-Museum Bochum, Herner Straße 45, 44787 Bochum, Germany; till.krieg@bergbaumuseum.de (T.K.); christian.mazzon@bergbaumuseum.de (C.M.)

**Keywords:** polymer degradation, plastics, rubbers, visual atlas, damage phenomena, museum survey, industrial heritage, ATR-FTIR, py-GCMS

## Abstract

Identifying the most vulnerable plastics and monitoring their deterioration is one of the main problems within heritage collections with historical synthetic polymers. Gathering and interpreting data about material and degradation phenomena in a collection reveals its conservation needs. A systematic survey of the collection can help towards this purpose. Surveys aiming at inspecting and documenting damages rely on several tools in order to fulfill their purpose. Firstly, objective descriptions of the damages that may appear, and secondly, the means of acquiring and interpreting material information. To address these needs, this article presents (a) a visual damage catalogue of degradation phenomena in plastic and rubber materials, and (b) the implementation of Fourier-transform infrared spectroscopy (FTIR) and pyrolysis–gas chromatography–mass spectrometry (py-GCMS) for the identification of analytically challenging rubber materials and of blooming phenomena. The damage catalogue is based solely on visual and olfactory signs, so that the assessment is independent of possible causes of damages and underlying processes, with the purpose of allowing objectivity to prime over interpretation. The limitations of the use of FTIR in the identification of heavily compounded rubbers in museum surveys is highlighted, and examples are presented. The use of py-GCMS on these cases conveniently allowed the identification of the constituting monomers of several rubber materials where FTIR could not provide a univocal classification of the material present. The study of several cases of blooming allowed the identification of diverse compositions and origins, showing that the description of a degradation phenomenon is only the first step towards its understanding. Unveiling the nature of a particular case of blooming is particularly critical when conservation treatments, such as the removal of a (potentially protecting) layer, are planned. For this purpose, attenuated total reflection-FTIR (ATR-FTIR) as a surface technique was particularly useful.

## 1. Introduction

The preservation of plastic- and rubber-based objects is a challenging task for conservators and conservation scientists alike [1]. Identifying the most vulnerable polymeric materials and those most susceptible to deterioration is one of the main problems within a collection. The documentation of damage phenomena in historical synthetic polymers and their changes with time provides information about the material stability, and is the first step for understanding ageing processes in plastics and rubbers (for the sake of clarity henceforth referred to as ‘plastics’ in this text). Carried out in the frame of a periodical survey, this information is helpful in collections planning, storage and treatment needs.

Supplementing this data with material information, while it may be demanding for most collections to perform, is a powerful tool for a number of reasons. Previous projects have highlighted the advantages of material analysis as the basis for the implementation of conservation measures in plastic collections. During the POPART project, for example, it became clear that it is important to know the (often complex) composition of plastics and rubbers because only by understanding the degradation processes can an appropriate approach to the problem of conservation and preservation be established. Identification of plastic materials confronts us with a challenge that requires appropriate analytical techniques often limited by the three-dimensionality of the object, the fact that it is not always possible to remove a sample and the need to support the results with reference materials. 

Critically, the identification and management of the so-called “malignant plastics” can have a beneficial effect on the welfare of surrounding objects. These plastics are known to cause damage to nearby objects by emitting corrosive gases upon degradation [2,3,4]. It is therefore of extreme importance to localise these plastics in a collection, to isolate them if possible [5], and to carry out regular monitoring campaigns. A systematic survey of the collection can help achieve this purpose. 

Surveys aiming at inspecting and documenting damages rely on several tools in order to fulfill their purpose. These include establishing objective descriptions of the degradation signs or phenomena that may appear; and the means of acquiring and interpreting material information, both material constitutive of the object and of the degradation phenomena, such as blooming, which may be found on the material.

### 1.1. Describing Damage in Plastics: State of the Art

Generally, the conservation state of the object is initially defined through a visual study of the signs of deterioration. The advantages of this approach are multiple: some plastics are recognisable from their characteristic damage phenomena, and the kind and extension of damage can be compared throughout timeto study the evolution of the object and how endangered it is. However, although recognised as a vital part of collection management [6], surveys relying on assessment conditions through the use of guides are not without flaw and their reliability has been questioned [7]. In research projects, it is particularly advantageous to document the damage in plastic objects as precisely as possible–by linking this information with material data and environmental conditions, whenever available, it may be possible to better understand certain damage phenomena and their implications for the object. Regarding chemical information, fortunately, portable and non-destructive analytical techniques, e.g., infrared spectrometers capable of performing measurements in attenuated total reflection (ATR) mode, are presently available that make it possible to qualitatively identify many plastics on site. 

Several projects and authors have previously documented and defined damages found in museum objects made of plastic materials. When gathering the data presented in this work, the authors were confronted with the need of documenting the different instances of damages appearing in the collection of the Deutsches Bergbau-Museum Bochum (see Supplementary Materials), so that these studies were reviewed and a comprehensive list of damages was compiled. 

In her article “A Survey of Synthetic Plastic and Rubber Objects in the Collections of the Victoria and Albert Museum” (2001), Brenda Keneghan listed and defined plastic damage encountered during a survey of five collections. The choice of collections and objects surveyed were subject to accessibility and time available. The collection in the Bethnal Green Museum of Childhood was, however, surveyed in its entirety, with the expectation of finding as many different plastics as possible. Together with conservators and scientists, definitions of damage specific to plastic objects in museums were, to the best of our knowledge, published for the first time [8]. Twenty-two different types of damages were divided into three main groups according to their origin or cause: chemical (from more to less common: ‘brittle’, ‘discoloured’, ‘yellowed’, ‘blooming’, ‘waxy’, ‘sweating’), physical (‘stain’, ‘abrade’, ‘scratch’, ‘crack’, ‘break’, ‘warp’, ‘torn’, ‘peeling’, ‘chip’, ‘craze’) and other (‘surface damage’, ‘missing’, ‘corrosion’, ‘loose’, ‘inclusion’, ‘biological’).

Ten years earlier, Jan Michaels [9] discussed the results of the survey carried out on the sound recordings at the National Library of Canada. The information obtained was relevant for collection management as well as for preservation and conservation planning. Depending on the type of sound carrier (e.g., open reel tape, shellac discs), rubbing and odour tests or physical examination were carried out and damage phenomena itemised, among them: ‘brittle’, ‘chips’, ‘creases’, ‘curl’, ‘dirt’, ‘distortion’, ‘flaking’, ‘peel off’, ‘plasticizer migration’, ‘scratches’, ‘stains’, ‘stickiness’, ‘stretching’, ‘tears’, ‘vinegar smell’ and ‘warpage’; these damages were however not defined. Some of the damages listed in this work are fairly specific of the format of the sound recordings surveyed, but, somewhat surprisingly, many could apply or relate to damages in any kind of plastic object. A more recent resource listing many of the damage phenomena in audio-visual carriers is the preservation glossary of the National Film and Sound Archive of Australia [10]. The comprehensive glossary mainly includes technical terms relevant for film and sound archives.

The Australian Institute for the Conservation of Cultural Material (AICCM) offers an online visual glossary [11] of damages usually encountered in museum objects. The comprehensive glossary was compiled by Australian conservators under the guidance of Alice Cannon, and not specifically designed for plastic objects. It explains the different types of degradation through a definition, aided by photographic examples. Of the 75 damage phenomena listed, seven are specific of plastics (‘brittle’, ‘chalking’, ‘ferrotyping’, ‘offsetting’, ‘softening’, ‘sweating’ and ‘vinegar syndrome’) and a further 49 can apply to them.

To the best of the authors’ knowledge, Shashoua [12] published in 2008 the first visual guide specific of plastic damage. She included seventeen terms, along with descriptions and pictures. The pictures allow to make the identification of damages less dependent on a definition that may be difficult to translate into other languages. The information is complemented with the underlying causes and the kind of plastics that can be affected by each of them. The defined terms can be seen below in Table 1. 

The “POPART” Project (Preservation of Plastic ARTefacts in museum collections, 2008–2012), had the aim to create, on a European scale, a strategy to preserve collections of artefacts composed of modern materials through conservation treatments and preventive conservation. In the frame of the project, surveys were carried out in several European museums. Particularly interesting were the results of the Victoria and Albert Museum, the Stedelijk Museum in Amsterdam, and three French museums: MAMAC (Nice), Musée d’Art Moderne (St-Etienne), Musée Galliera (Paris).

In the project webpage, useful tools can be found [13]. Apart from a survey form, a list of definitions for damage phenomena is available [14]. In a further document, a photographic documentation of damages for chosen case studies is presented [15], along with the most common damages for different plastics. This document also offers a list of typical damages shown by the most common polymers in museum context.

In these documents, the damages are classified in main categories (‘visible degradation’, ‘feel’, ‘smell’) whereas the category ‘visible degradation’ is divided into different subcategories (‘biological attack’, ‘colour change’, ‘deformation’, ‘deposit’, ‘others’, ‘other elements’). Overall, 39 different damage phenomena were mentioned, and 33 were defined. A photographic documentation is offered for 18 of them. 

Each partner modified the database developed in the project to fit their needs and requirements. Table 1 gathers the degradation phenomena surveyed in each case, compared to those proposed by Keneghan and Shashoua. All partners in the POPART project considerably enlarged the previously existing lists. 

As can be seen in Table 1, the damage list differs for the different museums, both in the classification of the damages and in the inclusion of different phenomena. This perhaps points out the difficulty in developing a single tool describing damage phenomena that is usable in every museum.

In the POPART surveys, the damages most commonly observed [16] (p. 136) were ‘scratch’, ‘dirt’, ‘yellowing’, ‘stain’, ‘crack’, ‘dust’, ‘corrosion’, ‘break’ and ‘discoloration’.

More recently, Bressan [17] has published a framework for the description of age-related symptoms in audio media as a diagnostic tool. The tool works as a module that can be integrated in existing databases, and includes a dictionary of symptoms: 49 items which can apply to one or more of the eight types of audio media considered in her work. She further proposes using this tool as the basis of a ‘hypermedia knowledge base’, a collaborative tool to gather data from different collections. The idea is that, as soon as a critical mass of data from community experience is reached, the tool may allow more consistent evaluations. 

The previous references together offer a comprehensive guide of changes specific to plastic objects, albeit not always supported by images and not in one single work. Although the importance of describing signs of damage and deterioration in a systematic way has already been stressed [18], a methodical description of the terms used to describe signs of damage in plastics and an accompanying photographic documentation of relevant examples has been seldom offered until now. However, different terms may sometimes seem to describe the same type of damage, making the choice of term used to describe it subjective. Similar damages need to be sufficiently differentiated to avoid overlap, so that a given damage cannot be described by several terms at the same time. 

### 1.2. Relevance, Occurrence and Identification of Rubbers in Heritage Collections

During their service life, and before they reach the museum, many industrial heritage objects require the presence of elements with elastomeric properties to carry out their function. This means having a high extensibility combined with the ability to recover from extension [19]. This property is related to a particular molecular, structural pattern of which natural rubber (as obtained from the tree *Hevea brasiliensis*) is the prototype. The fundamental features of this structure, common to rubber-like materials, are (a) a long, very flexible chain and (b) intramolecular forces between the chains. If, additionally, cross-links give rise to a three-dimensional structure, the material is referred to as a rubber. 

There exists a large number of rubbers and rubber-like materials. Apart from natural rubber (NR, a polyisoprene), butadiene rubber (BR), styrene butadiene rubber (SBR), chloroprene rubber (CR), nitrile rubber (NBR), ethylene-propylene-diene monomer rubber (EPDM), butyl rubber (a copolymer of isobutylene and isoprene, IIR) but also polyether- (EU) and polyesterurethanes (AU) and silicone rubbers (polyalkylsiloxane) can be named as the most important groups. The number of possible materials is huge, particularly taking into account that, in the final product, variability can be brought about not just by combining different monomers to give rise to copolymers, but also by mixing several polymeric products in blends. Added to this, a large number of different additives can be found forming part of the final product, among them are fillers, dyes, pigments, antioxidants, extenders, stabilisers, tackifiers etc. Indeed, elastomers are complex mixtures of several components, a normal formulation containing up to 20 of them [20]. At this point, it is worth mentioning carbon black, a typical filler and reinforcing agent often used in rubbers, and added to the rubber before vulcanization in order to improve the stiffness and the toughness of the materials, to enhance their resistance to fire or simply to reduce costs [21].

In museums, rubbers make up a relevant part of the macromolecule-based materials. For example, the surveys carried out in the frame of the POPART project all found different elastomers and rubbers [16]; it can be expected that the number of such materials in industrial heritage or technical museums will be higher. The problems concerning the storage and conservation of these materials in museums are widely acknowledged in the conservation community [16,22]. Rubbers may deteriorate by influence of heat, light, UV radiations, moisture and the presence of metal and oil; also biological factors can be part of the ageing process [23]. Particularly in rubbers displaying a significant amount of double bonds, the physical properties can be altered by oxidative processes, leading to chain scission and cross-linking, with appearance of new functional groups of high oxidation state. The chemical changes upon ageing cause substantial and even visible changes in the material. Hardening, brittleness, crazing and blooming [11] are some of the most common phenomena affecting this material group that have been described in the literature [24].

A wide range of analytical methods has been proposed for the identification of unknown rubbers (see for example [25,26,27]). Fourier-transform infrared spectroscopy (FTIR) is the technique most used in museum surveys, as it is able to provide a compromise between ease of acquisition (which has a direct influence on the number of spectra that can be acquired per unit of time), and relevance of the information that can be achieved with it. 

In general and in the ideal case, FTIR enables fast analysis and reliable identification of the generic type of base polymer(s) and bulk additives (inorganic fillers, plasticiser) by comparison with reference spectra [25] (p. 129). The development of reflection and attenuated total reflection (ATR) methods has also made non-destructive analysis possible, which has been dutifully implemented in museums [28,29,30,31]. The availability of portable instrumentation based on ATR currently allows to carry out these analyses in situ, a reason why this technique is most commonly used in museum surveys. The quality of the spectra acquired with this instrumentation, although it reaches in some instances that of benchmark spectrometers, cannot still be compared with the ideal case of the analysis of a small sample in an FTIR microscope. In the case of most plastics, however, it is sufficient to identify the main polymeric component. Thanks to the development of the ATR method, the analysis of insoluble resins and rubbers, for which it is not possible to cast thin films that can be analysed by transmission [32], has become much easier.

However, several authors have outlined the difficulties of analysing rubber materials by means of infrared spectroscopy. While the acquisition of the best spectrum possible can benefit from the use of different sample preparation techniques, most of them are not practicable for the application of identification of museum plastics, particularly so in the case of rubbers. For example, the presence of carbon black excludes analytical techniques using reflection [24] or limits its use as in the case of ATR [33]. In fact, carbon black interferes by absorbing substantial amounts of energy in the full spectral range [19], whilst inorganic fillers superimpose their spectra on that of the polymer or polymer blend [34]. Indeed, fillers like titanium dioxide or calcium carbonate often interfere with the spectroscopic analysis [35]; it has been described that the polymeric material often only makes up to 50% of the total mass of the sample [25]. The fact that vulcanised samples are not soluble in solvents limits the possibility of performing solvent extraction to analyse complex samples; here ashing offers an alternative but unfortunately only for the analysis of the inorganic components, not of the polymeric moiety. 

The analysis of the pyrolysis products by FTIR or gas chromatography–mass spectrometry (GCMS) has been extensively pursued [34]. The use of pyrolysis followed by chromatographic analysis allows a micro-invasive stuy on the object, a compromise that allows a deeper knowledge of the material and especially of its state of preservation. However, invasive sampling should be an exception in order to avoid loss of original material, and also for practical reasons –indeed, the aim of surveys is to acquire as high amount of data as possible in the least amount of time; hundreds of thousands of objects are not an exceptionally high number of holdings in museums. The whole process, from sampling, sample preparation to chromtographic analyses and interpretation, in those cases where the (bad) condition of the object allows having enough material for it, is generally too time-consuming, as opposed to fast in situ infrared analysis, to build the basis of material analysis in a survey. Nevertheless, in the case of rubbers, and for the reasons stated above, it is almost essential in order to determine the main components of elastomeric materials. 

### 1.3. The Analysis of Degradation Phenomena on Historical Polymers: Nature and Occurrence of Blooming in Polymeric Materials

Not an uncommon ageing or degradation phenomena, the nature of blooming appearing on a plastic or elastomeric part can be diverse. Previous authors have reported the appearance of blooming in polymeric materials in museums. They range from inorganic, such as sulphur blooming from rubbers [36], to organic compounds, such as fats [37], biocides (e.g., DDT [38]), fatty acids e.g., the lubricant stearic acid [12], plasticiser and flame retardant triphenyl phosphate [39] and organometallic compounds, such as the vulcanisation agent zinc stearate [36]. Oligomeric components in the plastic or rubber can also cause blooming. Their origin has also been documented to come both from chemical degradation, e.g., hydrolysis reactions (adipic acid blooming in polyester urethane objects [40]) as from physical processes, such as a reduction of the solubility of a given component in the plastic and rubber formulation [36]. In some cases, the ability of a plastic material to produce bloom can be a desirable effect, as in the case of components whose effect is to be delivered in the surface of the plastic or rubber: lubricants in O-rings and seals, biocides or antistatic agents, or the protecting effect against external ageing agents, such as light or oxygen of bloomed sulphur or wax in rubbers [36]. In these cases, blooming can be mistaken for a degradation phenomenon if not regarded in its material context, so that its identification becomes important. For the study of migration phenomena, such as blooming, due to the often relatively high purity of the substance migrating to the surface and causing it, FTIR analysis has proven to be a good method of identification. 

### 1.4. Aim of This Work

For a number of reasons, plastics in museums may exhibit highly heterogeneous degradation phenomena [41]. Although the kind of degradation processes possible may seem finite, the type of plastic in which it takes place, the influence of the manufacturing process, and the combination of all the ageing influences suffered, all have an influence on the final aspect of a given damage and may complicate their recognition, assessment and description. When documenting the present state of the objects in a collection, a set of well-defined terms are needed. While the reliability of assessment surveys has been shown to be poor, previous work has relied on barely defined and often overlapping damage categories [42]. While it is clear that the degree of complexity offered by a set of definitions will always be lower than that of the condition of the objects that need to be described, the complexity of a usable set of damage descriptors cannot be too far removed from it. In this respect, survey forms need to offer “a systematic approach to avoid overlap” and “ensure exhaustiveness” [42]. It has been described that “categories that are independent or mutually exclusive tend to have the highest level of reliability”, and that “faulty definition is one of the main contributors to unreliability” when several operators are asked to describe an item using categories [42,43].

The first aim of this work is therefore regarding the description of degradation phenomena in plastics found in industrial heritage accurately enough as to make their identification unequivocal and to avoid overlap. For this purpose, a visual damage catalogue of degradation phenomena in plastic and rubber materials has been developed. The approach presented here focuses on a purely visually (or olfactory) descriptive evaluation of the damage phenomena, regardless of the causes that may be behind the damage observed, using what have been called “observational terms” [39], with the purpose of allowing objectivity to prime over interpretation. 

The cause of a given damage cannot normally be established with confidence in the frame of a museum survey, and usually requires chemical analysis. Attempts to establish the origin and cause of a damage visually are usually based on interpretation and assumptions; it is therefore problematic to include them in descriptions. For this purpose, chemical analysis is needed to supplement and interpret the visual information, and to determine possible causes and underlying processes. Therefore, the second aim of this work is to present the implementation of analytical techniques for the identification of analytically challenging rubber materials and of blooming phenomena.

These aims were pursued in the frame of a survey performed at the Deutsches Bergbau-Museum Bochum (see Supplementary Materials for more details about the survey).

## 2. Materials and Methods

### 2.1. Infrared Spectroscopy

During the survey (see Supplementary Materials), objects were analysed by means of a portable infrared spectrometer from Agilent (4300 Handheld FTIR, Santa Clara, CA, USA), connected to a laptop and controlled by MicroLab Mobile Software (version B.05.4). The FTIR spectrometer can be operated in ATR mode using special sample interfaces. Apart from black plastics (e.g., rubber), where the ATR sample interface with germanium crystal was used, diamond crystal was used for the remaining materials (especially for hard materials or surfaces). The region scanned was 4000–650 cm^−1^, with a resolution of 4 cm^−1^. The number of scans was chosen according to the use of the handheld device: 16 to 32 scans if the part of the object was brought into contact with the crystal by hand, and 64 scans if the sample could be mounted on an ATR stage supporting the handheld device. In the stage, the sample is pressed onto the ATR crystal with the press arm. The latter method was also applied on loose samples. 

The analysis of small samples taken during the survey was alternatively carried out with an infrared spectrometer from Thermo Fischer, model Nicolet iS50, with gold optic, an ATR sampling unit, a KBr beam splitter and a DTGS detector. The region scanned was 6.000–400 cm^−1^, with a resolution of 4 cm^•1^. The number of scans was 64. The resulting spectra were compared with reference spectra from IRUG, commercial (S.T. Japan, Tokyo, Japan and Thermo Fisher, Waltham, MA, USA) and authors’ own databases. For a better presentation of spectra, they have been prepared using OriginPro 2019 9.6.0.172.

### 2.2. Pyrolysis–Gas Chromatography–Mass Spectrometry

Analyses were performed using a multi-shot pyrolyzer EGA/PY-3030D (Frontier Lab, Koriyama, Japan), coupled with a Thermo Fisher TRACE 1310 gas chromatograph with a split/splitless injection (SSL) port, and with a single quadrupole mass spectrometer unit (Thermo Fisher, ISQ 7000, Waltham, MA, USA). 

*Pyrolysis method*: Single shot pyrolysis was performed at 600 °C [44], and the pyrolysed sample was focused at −180 °C with the help of a CryoTrap at the beginning of the column. Sample 431b was additionally analysed using the following multi-shot method: shot 1: 300 °C; shot 2: 680 °C. The multi-shot pyrolysis temperatures were selected based on previous EGA/MS analysis. 

*GC method:* The interface and the GC injector were kept at 320 °C and 320 °C, respectively. The injection was done in split mode (split ratio 1:20). The chromatographic separation was performed on a metal capillary column Ultra ALLOY^+^-5 (5% diphenyl-95% dimethyl-polysiloxane, 30 m × 0.25 mm i.d., 0.25 μm film thickness, Frontier Lab). The oven program was: 40 °C for 2 min, 10 °C/min to 340 °C for 5 min. The helium (purity 99.9992%) gas flow was set in constant flow mode at 1 mL/min. A Vent-Free GC/MS adapter (Frontier Lab) was connected at the MS detector for a quick column exchange between the separation column and a capillary tube for evolved gas analysis (EGA). For a better presentation of the chromatograms, they have been prepared using OriginPro 2019 9.6.0.172. 

*MS method*: the system was run in electron impact ionization (EI, 70 eV) in positive mode with a scan range of 10–600 *m*/*z*. The ion source temperature was set at 270 °C and interface temperature (MS transferline temperature) at 320 °C. Perfluorotributylamine (PFTBA) was used for mass spectrometer tuning. Chromeleon 7 (Thermo Fisher) and F-Search (Frontier Lab) software were used for data analysis; the assignment of the peaks was done by comparison with mass spectra libraries (NIST MS Search 2.3, F-Search 3.6.2) and literature data as referenced in the text. Deconvolution was done with AMDIS (version 2.70).

Prior to each analysis sequence, the pyrolysis cups were cleaned (first with cotton buds and acetone, then they were placed in a beaker with acetone and put in an ultrasonic bath for 10–15 min; heavily dirty cups were cleaned again with cotton buds and acetone. Finally, all cups are placed in a muffle oven at 800 °C for one hour to remove all organic residues). A blank was then run of each to make sure that there were no residues left. Between all runs with sample, a blank run was performed to verify that the system was clean. 

## 3. Results and Discussion

### 3.1. Description of Damage Phenomena

Table 1 presents an overview of the degradation phenomena proposed in this work and how they compare to those found in the literature (see Section 1.1).

**Table 1:** Classification of damage phenomena according to Keneghan, Shashoua and as used in the surveys carried out during the POPART project, along with the terms proposed by this work. The terms in *italics* are terms considered to be related to the corresponding phenomenon used in this work (last column). The hyphen (-) indicates a term is not present in the work/column where it appears; an ‘x’ indicates that the term is present in the corresponding museum’s damage list.

Keneghan (2001)	Shashoua (2008)	POPART Project (2012)			This Work
		(a)	(b)	(c)	
					DEPOSITS Solid deposits
x	x	x	x	x	Bloom. Crystalline
-	*Chalking*	*Powdery*	-	-	Other
-	-	-	-	-	Pox
-	-	x	x	x	Dust
x	x	x	x	x	Stain
-	-	x	x	x	Dirt
					Liquid deposits
-	-	-	Fat	-	Smeary
x, *Wax*	x	x, *Exudation,*	x, *Exudation,*	x, *Exudation,*	Sweating
		*Droplet*	*Droplet*	*Droplet*	
-	-	*Unidentified*		*Other deposits*	-
					CHROMATICITY/ TRANSPARENCY Colour change
x	x	x	x, *Darkening*	x	Yellowing
-	-	x	x	x	Fading
x	x	x	-	x	Discolouration
-	-	x	x	x	Loss of transparency
					DEFORMATION
-	-	x	x	*Distortion*	Warping
-	-	x	x	x	Fold
-	x	x	x	x	Dent
-	x	x	x	x	Shrinkage
-	-	-	-	-	Channeling
-	-	x	x	x	Blistering
-	-	-	-	-	Softening
-	-	-	*Deformation, loss of original shape*	-	-
					LOSS OF INTEGRITY Usually involving loss of material
x	x	-	-	-	Abrasion
x	x	x	x	x	Chip
-	-	-	-	-	Flaking
-	Missing	x	x	x	Loss of material
-	x	-	x	-	Crumbling
x	x	x	x	x	Scratch
-	*Pitting*	-	-	-	-
					Not usually or necessarily involving loss of material
x	x	x	-	x	Brittleness
x, *Torn*	x	x, *Tear*	x, *Tear*	x, *Tear*	Crack
-	-	-	-	-	Craquelure
x	x	x	x	x	Crazing
-	-	-	x	-	Hardening
-	x	-	-	-	Loose
x	-	x	x	x	Peeling
x	x	x	x, *Split of parts*	x	Break
					SMELL
-	-	-	x	-	Vinegar
-	-	-	x	-	Acrid
-	-	-	-	-	Rubber
-	-	-	x	-	Camphor
-	-	-	-	-	Naphthalene
-	-	-	*Paraffin*	-	-
					OTHER/MISCEL-LANEOUS FEATURES
-	-	-	-	-	Overpaint
-	x	x	x	x	Corrosion (metal part)
-	-	-	-	-	Adhesive tape
-	-	-	x	-	Sticky
-	x	x, *Insect, Mould*	x, *Insect, Mould*	x, *Insect (‘Pest’), Moulding*	Biological attack
-	*Surface damage*	*Inclusion*	*Growing tree*	-	-

(a) Victoria and Albert Museum; (b) Stedelijk Museum/RCE; (c) French museums.

For a better discussion of the different degradation phenomena, in the following section the descriptions of the terms are arranged in categories according to the type of degradation to which they belong: (a) deposits, (b) changes in chromaticity and transparency, (c) deformations, (d) loss of integrity, (e) smell and (f) other/miscellaneous features. The discussion text after each table elucidates the rationale behind the decision to use the different terms as based on the definitions from literature and own experience. Descriptions needing more in-depth discussion, particularly those which may seem ambiguous in describing a given damage sign, are treated in detail. As has been mentioned above, the definitions avoid making a reference to the possible cause or origin of the damage (this is true save for a few exceptions where it was unavoidable: <dust>, <biological attack>, <sweating>/<smeary>). However, in the discussions below each table, possible causes are mentioned, where the information is available in the literature. For the discussions, further useful resources were consulted, such as the Art and Architecture Thesaurus^®^ Online [45] of the Getty Research Institute, the Conservation and Art Materials Encyclopedia Online (CAMEO) of the Museum of Fine Arts Boston [46], which also includes definitions of degradation terms, as well as the illustrated glossary of terms in the Lexicon of the American Institute of Conservation (AIC) Conservation Wiki [47,48]. In some cases, definitions of damages from academia and the industry have been taken into account. To this respect, the IUPAC definitions [49] and the German guideline VDI 3822 ‘Failure analysis’ in thermoplasts and elastomers has been particularly useful; in the case of the IUPAC, causes are often part of the definition, which is less aimed at describing the damage visually.

For each individual visual degradation phenomenon, a representative photo is available. In the Supplementary Materials, an alphabetical list of the damage phenomena has been included, along with their descriptions and a selection of further representative pictures to aid classification.

#### 3.1.1. Deposits. Table and Discussion

The category ‘deposits’ groups phenomena that involve accretions lying on the surface of the material, regardless of their external or internal (as coming from within the material) origin. Although the term <sticky> may well appear simultaneously to certain deposits and is probably in most cases a consequence of these (a surface showing sweating may appear sticky), this damage has been allocated in the category ‘other/miscellaneous features’, since it describes the *behaviour* of a surface, which at the moment of the visual assessment cannot be with certainty linked with the presence of a deposit.

The terms in this category have been grouped according to its aggregate state. Under the subcategory ‘solid deposits’, six different kinds of damages could be identified in the course of this work. 

Regarding ‘blooming’, the IUPAC has described it as the ‘process in which one component of a polymer mixture, usually not a polymer, undergoes phase separation and migration to an external surface of the mixture’ [49]. In the conservation community, a more descriptive approach has been proposed; in fact, it has been described that it can appear in several different forms [48,50]. In this work, two different kinds have been distinguished. Firstly, blooming recognisable as crystalline to the naked eye, usually shiny (as usually observed where additives or degradation products have migrated to the surface) has been named here as <blooming (crystalline)>. Secondly, blooming not recognisable as crystalline to the naked eye, is named here as <blooming (other)>. 

Keneghan [8] and the POPART partners include blooming (Keneghan: “powdery deposit on the surface of objects […] usually caused by the migration of additives”, POPART: “whitish deposit with crystalline aspect”). In this work, the term <blooming (crystalline)> with the meaning provided by the latter example, is favoured. 

According to the definition proposed here, <blooming (other)> accommodates deposits in general which are powdery, such as is the case of <chalking>, with the precondition that they are not crystalline to the naked eye. <Chalking> is used by the AICCM [11] to describe plastics turning powdery due to oxidation, with reduction of gloss. Shashoua [12] describes <chalking> as a white powder on surfaces, caused by separation of pigment or fillers, while she also describes <bloom> as a “white or grey matte film covering surfaces” as due to migration of antioxidants which happens mainly in synthetic rubbers. <Blooming (other)> corresponds thus to phenomena falling into the AICCM and Shashoua definitions of <chalking> and the Keneghan definition for <blooming>. 

<Blooming (other)> may not be easily distinguishable from mould [48]; here, the nature of the surface (whether the plastic on which it is found is likely to develop mould) and the ambient conditions (object having been exposed to high relative humidity at some point) should be considered (see below photographic documentation for biological attack).

It should be noted, however, that not all blooming deposits are damage phenomena, but that it may build a protective coating that is intended (see Section 3.2.2).

We propose the term <pox> to describe the kind of damage that is usually found in certain polyester urethane objects, including shoe soles (see Table 2). Usually of a constant size and fairly regularly distributed, the protrusions seem to be like <sweating>, an inside-out damage phenomenon. In the case of PU, they have been proven to consist of adipic acid arising from the degradation of polyester urethane [51].

**Table 2:** Definitions of the category ‘deposits’.

Deposits			
Solid deposits	Blooming	*Blooming (crystalline)* Crystalline efflorescence, where the crystalline character is identifiable macroscopically, e.g., through the identification of small crystalline particles and/or, depending on light incidence, a shiny reflection; typically white.	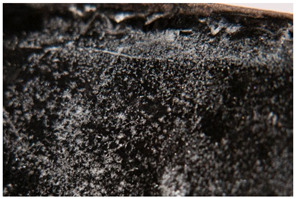
		*Blooming (other)* Amorphous deposit, macros-copically non-crystalline, of matte, powdery or waxy aspect, or (cloudy) film affecting large surface areas; typically whitish-greyish.	* 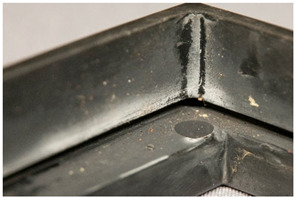 *
	Dried fluid	Solid material left behind as a residue after the drying of a liquid deposit. It usually appears in the form of a coloured, highly glossy semitransparent substance that sometimes shows some visual feature hinting to the previous liquid state of the substance (e.g., drop form).	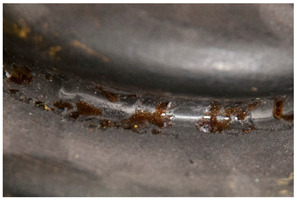
	Pox	Small protrusions or elevations with the form of a truncated pyramid with round edges, which may show a different form, colour or texture from the surrounding material.	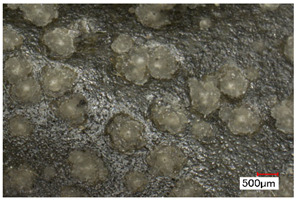
	Dust	Fine air-borne particles that deposit more or less extensively, and typically uniformly, in surfaces. Dust can be found either simply lying or adhered to these in combination with other substances (e.g., dirt deposition such as fat).	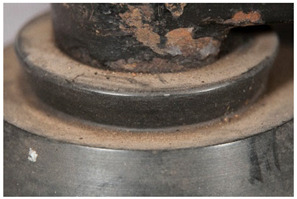
	Dirt	Solid soiling other than dust or stains, regardless of whether it can be found localised or extended in a wide area.	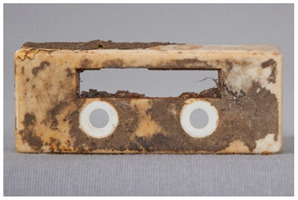
	Stain	Transfer of foreign material from an external object to the surface of study through rubbing off, spill or other process, causing changes in colour, texture and/or glossiness in a localised area, e.g., dash of colour.	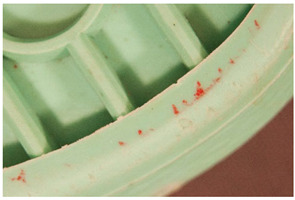
Liquid deposits	Sweating	Liquid phase occurring either in the form of drops (moisture) or of a film, of origin unknown; increased glossiness possible. Any liquid deposit that does not definitely have an external origin.	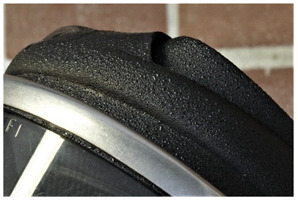
	Smeary	Oily or fatty residues, typically as a greasy film but not necessarily, coming from a known, external source (e.g., lamp oil); increased glossiness possible.	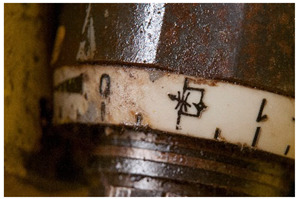

The term <dried fluid> is proposed here to account for observations made during the course of the collections’ survey (see Supplementary Materials). Depending on the ratio between volatile/non-volatile fractions of a liquid capable of drying, different phenomena can appear. In the case of liquids with (almost) no volatile fraction, the material may appear almost of liquid appearance, but reveal itself to be solid upon touch. In the case the volatile fraction is high, a mark appears in areas where the dried liquid has been lost (see Supplementary Materials). In the course of our research, certain dried fluids have been identified as arising from degradation processes, e.g., in the case of the reaction between acids and metals.

<Stain> can refer both to extraneous material lying on the surface (e.g., a dash of colour from rubbing off against another surface [14] (see also “Mark“ in the AICCM Visual Glossary [11]) as well as having diffused in it or impregnated it (e.g., a stain in a cloth). The second case requires the distinction from <discolouration> (a more thorough discussion can be found in the category ‘changes in chromaticity and transparency’), and from <dried fluid>, which has just been explained. A particular kind of stain is <tidemark> [14], where a liquid with a substantial volatile fraction dries, such as found in paper after water damage, leading to a colour gradient where the colour is usually darker on the edges. Another particular case is offsetting [14], for example, where the ink of printed documents is transferred to certain plastic materials. 

The definitions of <dust> and <dirt> are straightforward and do not require further detailing. It is interesting to note, however, that, when regarded from the causal point of view, they form, together with <stain>, a subcategory within the solid deposits since they are all different kinds of soiling and thus of external origin.

The second subgroup within deposits describes liquid residues on the surface of study, which, unlike dried fluids, remain liquid to the touch at the moment of study. This category includes high viscous deposits that can be found under ‘smeary’.

Several terms, among them <exudation>, <sweating>, <moisture> and <droplets>, have been used to describe phenomena said to arise from the migration or leaching out of components of the plastic formulation (plasticisers, stabilisers and fire retardants have been named here) or products of degradation. The available definitions agree in naming the build-up of droplets on the surface as the main characteristic feature, so that <droplet> seems to be the most correct term (moist, for example, limits the size of the drops). This would avoid inconsistency by using several names for the same phenomenon. The museums surveyed in the frame of the POPART project list both <droplet> (“small quantity of liquid”) and <sweating> (“liquid seeping out slowly onto the surface”); based on these definitions, it seems possible to have just one single term for this phenomenon that is then quantitatively documented by the aid of a rating scale. Furthermore, <exudation> and <sweating>, the terms mostly used in the literature and which seem to be synonyms, refer to the origin of the damage, which may not be easy to confirm upon visual examination. In contrast, the term <droplet> would allow for a more objective description of the damage. As an exception in this work, since in some cases this phenomenon can lead to formation of a glossy film, and due to its widespread use in the conservation literature, we propose the use of the term <sweating>. It should be noted that, in some cases, this phenomenon can lead to stickiness. 

In other contexts [36,52], the term bleeding is used for “oily films formed on polymer surfaces”, thereby having a similar meaning to sweating. However, in the conservation community, bleeding has been consistently used for the migration of colour-providing particles into surrounding areas [11,45,48]. 

Although indistinguishable in aspect from <sweating>, we propose the term <smeary> to designate liquid residues with a definite external origin (e.g., grease from fuels or lubricants like lamp or engine oil). This phenomenon is quite common in industrial heritage. The difference between <smeary> and <sweating> is thus solely the origin of the deposit: a smeary, greasy film mainly having an extraneous origin, while <sweating>, such as the related terms <droplets> and <exudation> indicating a degradation process in the material as its origin. Unfortunately, from a purely visual point of view and without further analyses, a differentiation is not always possible. Here it is proposed to use <smeary> for those cases where the external origin is distinct from the context of the phenomenon, and <sweating> for every other liquid deposit without a known external origin.

#### 3.1.2. Changes in Chromaticity and Transparency. Table and Discussion

The group of phenomena within ‘changes in chromaticity and transparency’ includes colour changes such as <yellowing>, <fading> and <discolouration>, and <loss of transparency> (Table 3). In the colour changes, an intrinsic, chemical change in the material mostly plays a role, as opposed to e.g., <stain>, where an extraneous material is involved per definition, and which usually involves physical processes (e.g., dye migration).

Deciding if there is an actual change implies a precise knowledge of the original colour or transparency level, e.g., from pictures or protected or covered areas, which represents a challenge and usually depends on the quality of the original documentation, if available.

Both <yellowing> and <fading> appear usually in areas exposed to the light and/or the surroundings. The <yellowing> phenomenon is mainly visible in light-coloured materials, whereas <fading> can be best distinguished in darker ones. Both of them can appear as a gradient or as a uniform change along a surface, which further complicates the documentation of the extent of the damage. According to the offered definition, <yellowing> is also used to document a darkening of the material [1] (p. 263, polyethylene terephthalate; p. 289, polyamide), for which other authors use the alternative term of darkening [14].

While <yellowing> and <fading> are relatively accurately described, there exists a number of possible colour changes which do not fall into any of both terms. In this work, any colour change phenomenon not taking the form of one of these well-defined damages has been classified as a general <discolouration>.

It has been described that some of the phenomena in this group can be both caused by the effect of light or pollutants (see, for example, the definition for <discolouration> in [11]). This is probably also true for the whole group of colour change.

As mentioned above, it is necessary to clearly distinguish between <discolouration> and <stain>. The main difference is whether the colour change has happened in the surface material itself or whether there is material lying on the surface. According to this, while colour changes in general can be mostly ascribed to a chemical change of the original material (also possible is a washing out of dye during cleaning procedures in textiles, a physical process), <stain> is here considered to be essentially a colouring that occurs through the action of an external substance e.g., through fluid dye. 

In this work, transparency changes, as proposed by POPART, have been considered as belonging thematically to the chromaticity changes. The subcategory and term <loss of transparency> is only visible in transparent or sometimes also translucent plastics, as may be caused by surface texture change. It can be a result of other damages such as <abrasion>, <crazing> or <yellowing>. 

**Table 3:** Definitions of the category ‘changes in chromaticity and transparency’.

Changes in Chromaticity and Transparency			
Colour change	Fading	Reduced colour brightness or intensity.	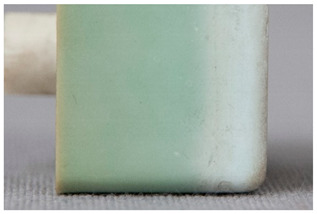
	Yellowing	Colour change to a yellow tinge/hue, which can sometimes be darker (red/brown). Easily visible in clear or lightly coloured objects.	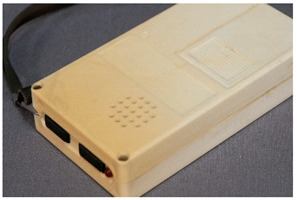
	Discolouration	Any change in colour of the original material other than fading or yellowing.	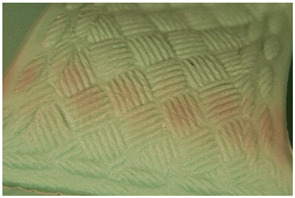
Loss of transparency		Increase of the opacity of a transparent or translucent object.	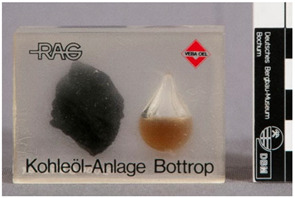

#### 3.1.3. Deformations. Table and Discussion

Deformation phenomena are described here as those in which any change in the form, i.e., in any of the object dimensions, takes place without involving a loss of material (Table 4). Among the causes for these changes can be an external mechanical force, as it happens in <fold> or <dent>; heat, such as it can happen in <warping> and <softening>; a loss of adhesion between layers (as it may happen in <blistering> and <channelling>) or deeper changes in the material (<shrinkage>, <brittleness>). In fact, an existing definition of ‘deformation’ as damage phenomenon has already been described by considering its causes (“…occurs when a material loses its strength and creates an area of collapse. Can also occur where an external impact or pressure causes a depression or indentation.” [11]). Although rather permanent in nature, some of them may be, at least initially, reversible (particularly <fold>; <dent>. According to Shashoua [12], <dent> is a “deformation, either permanent or temporary”, with the cause being a “physical damage–perhaps due to use; thermal damage from overheating”.

The category ‘deformation’ could well be brought within the category ‘loss of integrity’, since a deformation can imply a certain loss of integrity of the object or of the material (see below). For the sake of clarity, however, this category forms a group in itself in the present work. Ultimately, the structure that is chosen to organise the different damages is only relevant in so far that it aims chiefly at organising the different damages in related fields of meaning in order to make the atlas a practical tool. 

Typical deformation phenomena are <dent> and <fold>. <Dent> is described here either as a hollow mark or as an indentation in a material, and is usually a consequence of an external, mechanical force. Related terms are <buckling> (“Distortion caused by shrinkage or compression” [11]) and <distortion> (“A concave, convex or twisting change of form, used to describe stiff organic materials that have become misshapen, such as paper, card, plant fibres and wood”, [11]), particularly the latter. In the case of <buckling>, the authors also consider shrinkage as a possible cause, but the term remains otherwise somewhat unspecific and could also be used to describe damages, such as <warping>. In the present work, <dent> was favoured over <buckling> and <distortion> since it is used by damage atlas specific of plastics. Shashoua mentions the effect of heat as a further possible origin for <dent>. In the present work, due to the accompanying changes, e.g., in the texture, a deformation caused by the effect of heat would fall within the term <softening>.

Additionally, due to an external mechanical force, <fold> may lead to irreversible changes due to the ability of plastics to creep (cold flow). The term <crease> has also been defined [9], the main difference being that <fold> takes place intentionally, whereas crease does not. For the sake of clarity, and because a further distinction may be both unnecessary or lack a further benefit as well as be difficult to point out, in the present work only fold has been included. Similarly, the term <rolled> has been used by the AICCM to describe materials that retain the shape after being tightly curled. This phenomenon is related to both <fold> and <warping>; regarding the intention this kind of damage is probably best included under <fold>.

<Warping> usually affects films, foils or thin materials of a few millimetres, and, contrary to <dent>, usually affects the whole of it. For the term <warping>, both Keneghan and AICCM describe an object which is out of shape or whose original dimensions have changed, respectively; these could be considered general definitions of the category term ‘deformation’. Keneghan refers to possible causes such as loss of additives, water, and built-in stresses; the latter are also mentioned by AICCM (Shashoua [12] uses the term <dent> to define this degradation phenomenon). 

Different types of deformation are described by the terms <channeling> (e.g., as caused by shrinkage that leads to separation of layers) and <blistering>. For the latter, the first part of the definition from the AICCM glossary has been adopted. These phenomena do not involve the detachment of a large part (the totality in extreme cases) of the layer but, as per definition, a channel-like and bubble-like delamination, respectively. 

The phenomenon <softening> can be perceived visually through a loss of form or certain changes in the surface, contrary to the related term <hardening>, which is here classified under damages involving a ‘loss of integrity’ without loss of material (see below; it should be noted that, in this context, <softening> is not meant to refer to ‘smooth’, i.e., the surface characteristic that is the opposite of rough). Due to chemical changes in the polymer, such as hydrolysis in polyester urethane, or heat exposure, a liquefaction may take place and, in a worst case scenario, <softening> may lead to a total loss of shape and total collapse. As in <hardening>, <softening> causes an irreversible change in the state of the plastic material and its original shape cannot be restored. Stickiness can also accompany this phenomenon, as well as a change in gloss. The term <softening> is also used after a recognizable re-solidification following a softening process, in order to acknowledge the fact that a softening has happened at some point in the life of the object.

**Table 4:** Definitions of the category ‘deformations’.

Deformations		
Blistering	Raised area, bulge or bubble on an objects’ surface, often between adjoining layers of different materials [9].	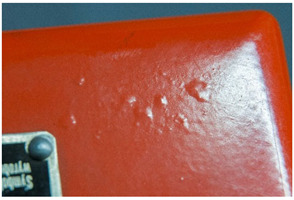
Channeling	Channel-like detachment between a foil or coating and their undercoat.	
Dent	Three-dimensional form change appearing as a hollow mark, buckle or bump; indentation.	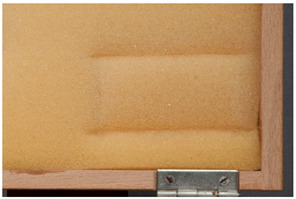
Fold	A bend, crease or kink of an object on itself, causing after a certain time stress in the material, eventually leading to partially irreversible damage.	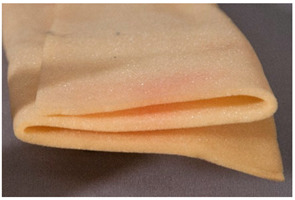
Shrinkage	Dimensional reduction of the volume of a material; it may appear together with cracks and breakage.	
Softening	Loss of hardness or firmness of the material, in extreme cases up to the point of liquefaction, which may have happened at some point in the material life. A (re)solidification may have taken place meanwhile, but the loss of original form, surface texture, glossiness or other changes hint towards a previous softening.	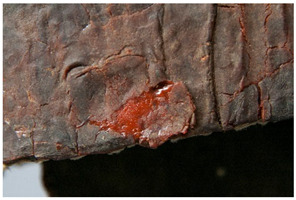
Warping	Three-dimensional form change appearing as concave and/or convex distortion or twisting, sometimes in the form of a wave, so that the material/object surface is no longer flat.	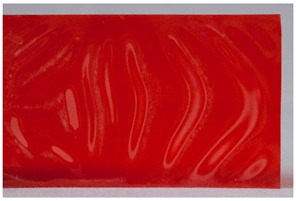

#### 3.1.4. Loss of Integrity. Table and Discussions

This category describes damages that may endanger the integrity of the object. The word ‘integrity’ is used here in the sense of ‘the quality of being whole and complete’ [53]. This type of damage may or may not lead to a loss of material; therefore, the division into two subcategories, namely, damages that intrinsically involve detachment and/or loss of material (Table 5); and damages that do not usually or necessarily involve a loss of material (Table 6). Here, loss of material is meant as the ultimate loss of the detached material, which is therefore missing. 

In some cases, the terms used refer to a behaviour of the material rather than a visual damage (e.g., <hardening>, <softening>, <brittleness>). In most cases, the behaviour is noticed indirectly through the damages it causes (e.g., <crumbling>). If possible, it may be useful to perform a small, minimally invasive test in order to better assess the behaviour of the material. It should be assessed on a case to case basis whether the test and the minimally invasive damage caused is justified or not, as opposed to the use of the information that may be drawn from the test results.

Within the first subcategory, <abrasion> describes a damage that results from the process during which a superficial fraction of the object’s material is removed. Wear is usually the main cause of these types of marks due to a repeated friction or contact in a particular direction with other surfaces [11,14]. This definition shares a commonality with that of <scratch>. In contrast, the latter describes a usually single or isolated mark, which may be deeper than abrasion. Other authors [11,12,18] consider <scratch> not to necessarily involve material loss, and use in their definition the words <cut> or <indentation>, which do not necessarily involve loss of material but rather a damage or deformation in a surface. Intuitively, however, it is difficult to think about a scratch not causing at least a small material loss. In this work, <scratch> has been thus classified under the present subcategory. 

<Abrasion> groups two slightly different but related phenomena with a common origin (wear, friction) but different manifestation, depending on the materials involved and/or the kind of wear responsible for the damage. Firstly, a local accumulation of marks causing roughening of the surface and concomitant gloss reduction, and, secondly, the smoothening of a surface due to the polishing effect of the repeated friction against a second surface, and which is accompanied by gloss increase [50] (p. 7).

Keneghan [8] uses the term <chip> together with <break> (“Break/chip: Both definitions may be used to define a fault running through the sample dividing it into two or more pieces. The difference is in the size of the damage, with a chip being the smaller.”) and differentiates them mainly based on the size of the pieces that are formed. The definitions given by the AICCM (which uses <spalling>: “Where small pieces of stone flake off or split into chips; usually caused by frost damage.” as a term, [11]) and POPART (“Small part missing from an object surface or edge; the fragment broken off from the whole.”, [14]) are more precise, even if in the first case the authors refer to a different material than plastics. A punctual loss of surface material (usually in the proximity of edges) best describes the visual damage phenomenon <chip>. 

<Crumbling> leads in its final stages to extreme detachment of material and/or complete loss of integrity of the object. Both size and shape of the material detaching from the object, as well as the specific plastic where the degradation is taking place, are important for the classification of the damage. While foams crumble in their final stages in the form of powder [14] or dust, rubber may crumble showing cube break loss. Contrarily, <flaking> [45] applies to coatings [46] (“two dimensional objects”) or bulk materials [19,48] (“three dimensional objects”) in which the substance being detached separates itself in the form of flat platelets. It has been stated [46] that flaking is generally due to a combination of adhesion loss and cracking. 

<Crumbling> and <flaking> should not be confused with <craquelure>, which does not involve a detachment from the underlying layer or support and is consequently classified under ‘loss of integrity’ without necessarily involving loss of material. 

<Loss of material (other)> is proposed in this work for any other kind of damage involving loss of material which does not fall into any of the definitions outlined above. Sometimes, due to the fact that the detached material is missing, it is not easy to trace back the kind of damage that has taken place.

<Pitting> has been so far only defined by Shashoua, to the best of our knowledge. There, pitting is defined as “craters or pits on surfaces not caused by mechanical damage”. The evaporation of plasticizers and solvents during curing or shaping is mentioned as its cause. Pitting is a typical damage phenomenon found in metals and glass, and it has also been defined in plastics as a damage which is due to manufacture [54]; for this reason, it has not been included in this list. 

This subcategory of damage does not necessarily involve a loss of constituting material (Table 6). It groups phenomena that imply either partial or complete separation of material, which is not lost. Changes in elasticity, most typically exemplified by hardening, are also included here to account for the fact that such changes also affect the integrity of the object, without involving loss of material.

<Break> is used for a complete separation of a part of the object [14], which has not resulted in <loss of material>, the resulting pieces being still available. It can be defined as a crack that has been completed or carried out to its extreme [55] (p. 83). For Keneghan, only the size distinguishes a break from a chip, the latter being smaller. In this work, since <chip> involves losing a fraction of original material, it has been classified differently than <break>. As defined by Keneghan [8], tear may be considered as a special case of break happening in a sheet “(tear) is used to define damage to a thin sheet of plastic where it is pulled apart or into pieces by force”), and POPART further describes it as “an opening made by pulling apart”; for the sake of clearness, it has not been included as an extra term. We propose that tears are documented under the term <break>. The term <loose> can be used to describe material at risk of being broken or lost. Moreover, stickers or labels may be in a loose state when adhesives begin to fail. It may therefore be important to document loose parts in an object so that action may be taken before further damage in form of actual loss can happen. 

<Cracks> appear in a variety of materials in different forms. They can be considered to appear in more specific forms in phenomena, such as <crazing> and <craquelure>. While cracks do not involve material loss, a scratch always involves some removal of material, as has been said above. <Cut> could be defined as a different kind of damage, since it implies intentionality; however, for the sake of practicality and since there is not much added benefit from distinguishing between <crack> and <cut>, it has not been included as an extra phenomenon in this work. If necessary, and since cuts do not involve loss of material, they could be classified under cracks (preferably than under the term <scratch>). 

<Crazing> may refer to two different but related phenomena. The first describes the fine internal micro-cracks (crazes) which may occur particularly in transparent material, typically amorphous, glassy polymers, as a result of certain kinds of stress and which feature zones with highly oriented polymer chains, also called stress whitening. This is the established definition of the phenomenon <crazing> in the field of damage analysis [55] (p. 84ss) and which is referred to in the IUPAC definition (“crack-like cavity, formed when a polymer is stressed in tension, that contains load-bearing fibrils spanning the gap between the surfaces of the cavity”, [49]; “the crazed areas are composed of polymeric material of lower density than the surrounding matrix” [56]) and which probably is at play in previously published reports [57]. The second describes the typical degradation process (“Development of a thin network of cracks“ [14]) taking place in cellulose nitrate and visible in transparent or translucent objects made of this material: cracks spreading throughout the material, forming a network which leads to concomitant whitening. Other authors define it as a network of cracks in the surface, e.g., Keneghan and AICCM, where it is described as a purely superficial event typical of varnishes and coatings. Meanwhile, Shashoua speaks of interconnecting cracks that may also be present deeper inside the material, in accordance with specialised literature on cellulose nitrate [58] (pp. 35–41). 

**Figure 1:**
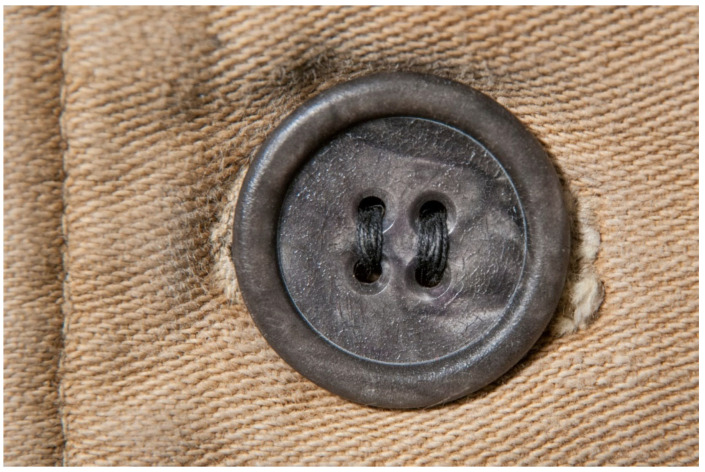
Button from a miner’s jacket with craqueled surface.

In this work, and in order to delimit it clearly from <crazing>, a network of cracks only in the surface is referred to as <craquelure>, for which Figure 1 is a good example, and as typically found in paintings. As it happens in paintings, craquelure in plastic objects is also an interface phenomenon that is due to a different behaviour of adjacent layers. As such, it can also arise in an object between an outer, more aged area, which builds up a layer with different (physical) properties as a result of ageing or exposure to the environment, and the underlying bulk material (see Supplementary Materials, Figure S28). Ordinary cracks without typical characteristics of <crazing> and <craquelure> are designated as <cracks>.

The term <brittleness> (“Material became weaker hence easily cracked or fractured” [14]) describes a certain behaviour or property of a material, as <hardening> or <sticky>. Brittle materials typically show cracks and breaks, constituting material detaching itself as small particles. In materials science, brittle materials show a lack of plastic range and a very small modulus of elasticity, directly breaking when submitted to stress. It should be noted that not all materials with cracks are brittle. Brittleness may be the consequence of <hardening>, and thus occur simultaneously, but not necessarily. All four consulted authors who provide a definition for this phenomenon agree in defining a material as brittle if it breaks or cracks when subjected to pressure or bending [8,11,12,14]. Contrary to Shashoua, it is not defined as a purely superficial phenomenon but as typically involving all its thickness. Among the cited causes, degradation processes, such as loss of additives, oxidation, reduction or increase in molecular weight, the latter caused by cross-linking, are possible.

The term <peeling> describes the detachment of a layer, which peels off as a large piece and usually curls. The AICCM describes this phenomenon also with the term <delamination>. However, the IUPAC describes delamination as a “process that separates the layers of a laminate by breaking their structure in planes parallel to those layers” [49] thus not referring to a general case but to those of laminated materials. Differently from <flaking>, it does not intrinsically imply loss of material. Another difference between both terms is the size of the material detaching itself; while in <peeling> large areas detach, in <flaking> small, flat platelets are separated from the object. The definition of <peeling> offered by POPART (“Outer layer coming off in flakes or small pieces” [14]) corresponds to the definition of <flaking> in this work (see <flaking> above).

A behaviour typically fitting to ‘loss of integrity’ without necessarily involving a loss of material is the phenomenon <hardening>, which has also been described as the stiffening of a material [14]. Not a visually perceptible damage *per se*, a minimally invasive pressure test, as well as some visual characteristics, can help to identify hardened plastics. Due to the resulting loss of elasticity (ability of a deformed material to return to its original shape when the stress is removed) and plasticity (ability to deform under stress), there is a risk of fracturing [59]. 

<Hardening> and <brittleness> do not overlap per se–as mentioned above, some materials may be brittle as a consequence of hardening, but not necessarily. An example that helps visualise a material that is brittle but not due to hardening is the case of certain polyester urethane materials, such as typically found in shoe soles. Upon ageing, the material becomes soft and cannot stand pressure without breaking. 

#### 3.1.5. Smell. Table and Discussions

Although not falling into the category of visual damages, documenting the smell of certain plastics can at times be useful (Table 7), both as a tool for the identification of certain plastics, as has been proposed [60,61], as well as a possible signal for the emission of degradation products. The best-known case of a degrading plastic identifiable through its smell is cellulose acetate. Suffering from the so-called ‘vinegar syndrome’, the acetic acid released upon hydrolysis of its acetate groups is responsible for the distinctive smell. 

Generally, an acrid smell can be due to the release of acids upon degradation of the so called ‘malignant plastics’, such as nitric acid from degrading cellulose nitrate.

Describing smells that are not so commonly known as that of acetic acid involves a certain degree of subjectivity. Training with material identified by other means (e.g., FTIR), or ordered from a reliable source, will increase the reliability of the confidence of the assignment, as will training with an experienced colleague (see also reference above). After using these resources, rubber, camphor, naphthalene or phthalate smells will be easily recognisable. 

It should be noticed that, in composite objects, particularly present in industrial heritage, it may be difficult to identify the origin of a given smell. 

There are several articles [62,63], describing the emissions coming from historical plastics. The analysis of volatile organic compounds (VOCs) provides potentially an opportunity to further enlarge the present list of identifiable odours by making a link between the substances found in emissions from the degrading plastics, their smell, and the smell of the degrading plastics. This may help pin down the ‘meaning’ of typical smells in museum collections and archives such as paraffin or phenolic smells. This information, together with the chemical nature of the plastics in which they are noticed, can be useful in the future for the interpretation of different smells.

#### 3.1.6. Other/Miscellaneous Features. Table and Discussions

This category groups phenomena not fitting elsewhere or not necessarily being a damage per se (Table 8). 

The phenomenon designated as <overpaint> distinguishes itself from <stain> in the intended purpose it implies. As can be seen above, an overpainting is described as the intended coating of a wide area–often applied in a museum context, particularly in an Industrial Heritage context, for example, with a conservation or pedagogic purpose. While <overpaint> is not necessarily a damage, it is worth documenting and evaluating because of the possible influence on the appearance of the object, e.g., ageing lacquers originally applied as protective coating. The same applies to <adhesive tape>, which may currently or in the future be the origin of damage.

Similarly, there can be advantages in documenting <corrosion (metal part)>, in itself not directly a plastics damage but which may offer potential evidence of the presence of a malignant plastic in the surrounding of the metal part. Corrosion in metals is the result of an electrochemical process [45]. Although not always necessarily connected, an evaluation of this phenomenon may be of use when identifying and detecting plastics emitting corrosive gases.

<Biological attack> groups any kind of damage caused by living organisms. Especially in the open air, there exists the possibility for e.g., lichens and moss to grow on museum objects, causing long term damage. Animal excrements can also be chemically aggressive against plastic materials. Larval cases and gossamer may not be typical signs for plastics damage, but an infestation of harmful insects in the environment of a plastic part can be a later reason for damages, e.g., loss of static stability or chemical reactions caused by excrements. The occurrence of mould is particularly relevant, since it requires taking particular steps such as control of relative humidity. Mould is not always easily identifiable to the naked eye; it is possible that mould appears as a blooming or stain that may be classified under those particular damages.

The term <sticky> [53] has been included here for lack of a better place–neither ‘deposits’ nor ‘loss of integrity’ seemed to be able to allocate it. <Sticky> is related to the viscidity or viscousness of the surface. The documentation of sticky surfaces is crucial for the conservation of objects affected by this phenomenon, since the accumulation of dust on them will require special conservation treatments. Also, the anomalous accumulation of dust may be indicating an underlying sticky surface. In the course of the survey (see Supplementary Materials, Section S1), stickiness was usually present simultaneously with <softening> and different deposits. 

The term ‘change in gloss’ has been previously described by the POPART project and by the AICCM glossary as a change in the shiny quality of a surface. A number of phenomena are indeed accompanied by changes in the glossiness of the surface, and changes in glossiness are included in the corresponding descriptions (e.g., <abrasion>, <softening>). However, it has not been included in this work as a unique phenomenon, since it seems difficult to locate an example in which the change in gloss alone appears as an independent phenomenon (the term ‘ferrotyping’ is also related to a change in gloss, see https://aiccm.org.au/visual-glossary/ferrotyping/ and https://www.nfsa.gov.au/preservation/preservation-glossary/ferrotyping, last accessed on 18 February 2021).

### 3.2. Analytical Challenges of Surveying Industrial Heritage Collections

In the course of the survey that motivated this work (see Supplementary Materials, Section S1) and the analytical study that followed, several challenges characteristic of the analysis of plastics in museum context appeared. In this section, some examples are presented focusing in (a) the identification of rubbers, one of the most important groups of materials in technical museums, and (b) blooming, one of the most common kinds of deposits that can be found on plastics and rubbers. In the case of blooming, the analysis of both deposit and plastic or rubber on which they have appeared is essential, since they are not always indicative of a damage phenomenon. 

#### 3.2.1. Identification of Rubbers

In this section, the authors will be presenting practical examples of the challenges faced when attempting to identify the main components of elastomeric materials by FTIR. During the survey that prompted this article, the ATR-FTIR analysis of parts with elastomeric properties often posed analytical challenges while trying to identify the chemical structure of their main components, the high content on inorganic components and ageing playing an important role. This is the case of the objects and plastic parts detailed in Table 9. In this section, some examples of aged elastomeric materials from the survey at the Deutsches Bergbau-Museum Bochum are presented along with their spectroscopic and pyrolysis-GCMS (py-GCMS) data, with the purpose of identifying their main components.

**Figure 2:**
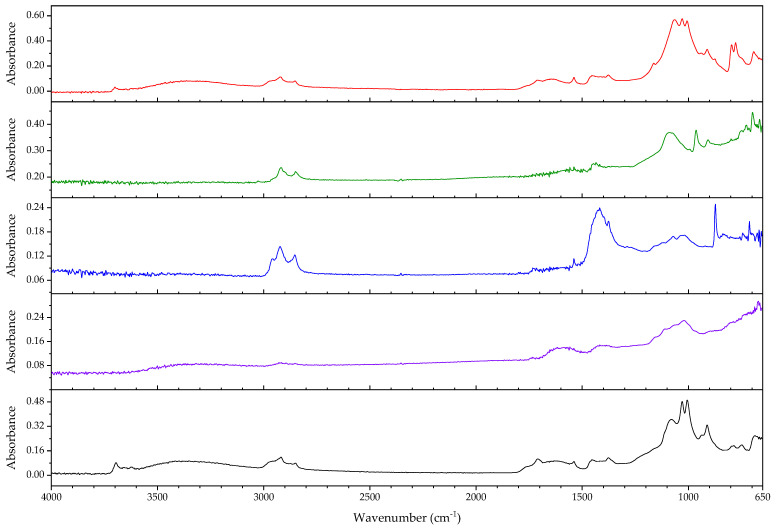
FTIR spectra of the samples detailed in Table 9. From top to bottom: 194, 383, 431, 530 and 598.

In Figure 2, the spectra of the samples described in Table 9 are shown. These spectra were acquired during the survey with a portable FTIR spectrometer, and its quality reflects the challenges of measurements in situ, which are dependent on factors such as the use of portable instrumentation, a suboptimal contact between sample and ATR crystal, and the analysis of strongly aged materials. In this respect, they also reflect the challenges of surveys, where analysis should happen without sampling due to output needs (number of objects/time). During the survey, two ATR crystals were available for analysis, diamond and germanium. Due to the presence of strongly absorbing carbon black in many samples, the germanium crystal usually gave better results [64] (p. 21). 

**Figure 3:**
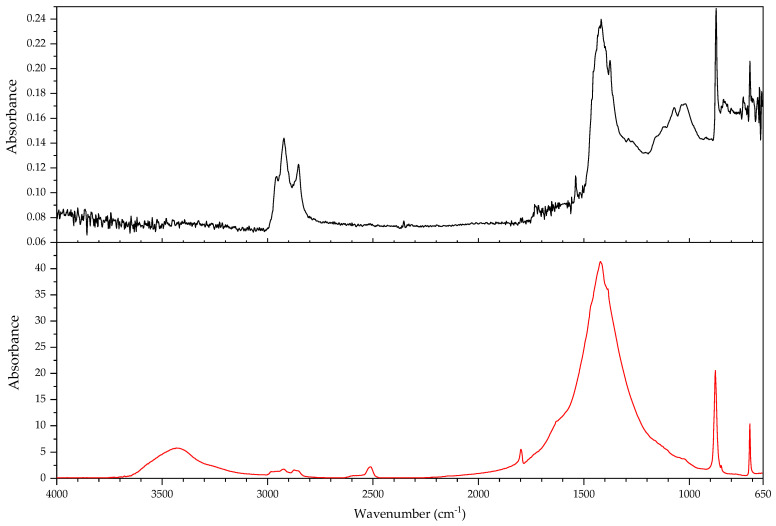
FTIR spectrum of sample 431 (black line) compared to reference spectrum of calcium carbonate (red line) from a commercial database.

**Figure 4:**
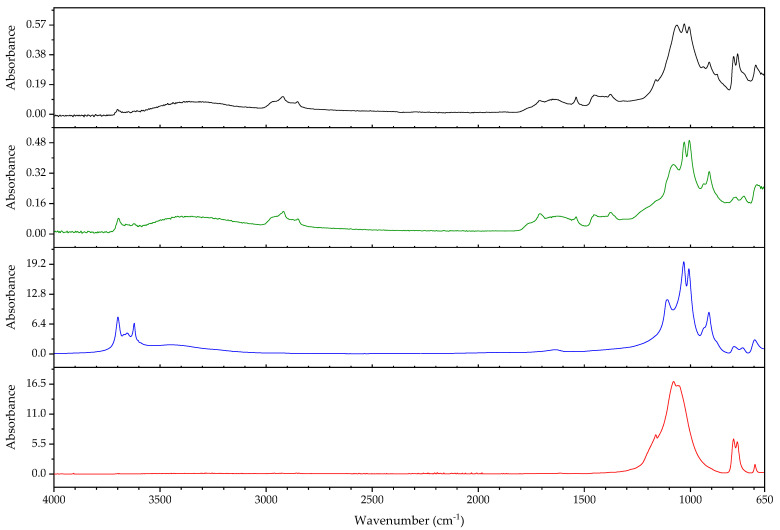
FTIR spectra of samples 194 (black line) and 598 (green line) compared to reference spectra of clay (blue line) and quartz (red line) from a commercial database.

**Figure 5:**
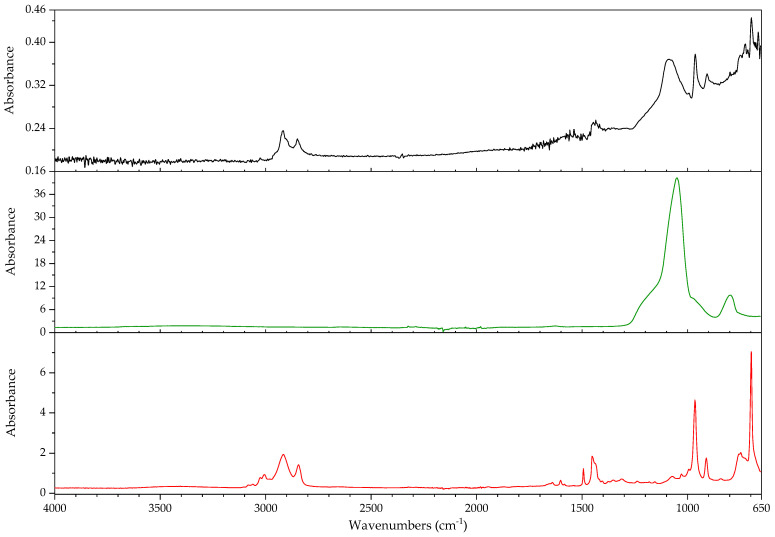
FTIR spectrum of sample 383 (black line) compared to reference spectra of silica gel (green line) and styrene/butadiene block copolymer (red line) from a commercial database.

In all cases, an inorganic component is responsible for the main bands; in fact, most vulcanisates contain a high proportion of a strengthening filler [64]. The sample 431 (Figure 3) shows calcium carbonate, with bands at 1421 (asym st CO_3_), 873 (asym def CO_3_) and 713 cm^−1^ [64], a typical filler [23]. Samples 194 and 598 (Figure 4) show as main component a clay mineral, with characteristic bands at 3700 (sample 194) and 3696 cm^−1^ (sample 598), 3624 (598), a shoulder at 1112–1113 cm^−1^ and sharp bands at 1029, 1005 and 912 cm^−1^. Sample 194 also shows the presence of crystalline quartz, with bands at 1163, 1064 (st –O–Si–), 796 and 778 cm^−1^ (def –O–Si–). Finally, sample 383 (Figure 5) shows some bands characteristic of SBR; particularly clear are the trans –CH=CH– group vibrations of butadiene at 963 cm^−1^), 907 cm^−1^ (=C–H out of plane bending of vinyl groups) and 697 cm^−1^ (aromatic C=C oop bending of polystyrol) [34], and a broad band at 1088 cm^−1^ that could be due to the presence of amorphous SiO_2_. 

The main features in these spectra are thus an inorganic component which sometimes can barely be recognised, such as in the case of sample 530, bands in the C–H stretching region (3000–2800 cm^−1^) and a badly resolved area between 1800–1500 cm^−1^, where some of the spectra show similarities. It is difficult to identify the main organic component based on the features present in this area, and because the inorganic components are likely obscuring other bands belonging to the organic moiety.

In order to study the main polymeric component of the samples in Table 9, py-GCMS analysis was performed on small samples of the relevant plastic parts. Table 10 gathers the main markers found in these analyses. To aid the chromatographic separation of the low molecular weight pyrolysis products that are typical of polyene based rubbers, the pyrolysed sample was focused at −180 °C at the beginning of the chromatographic column.

**Figure 6:**
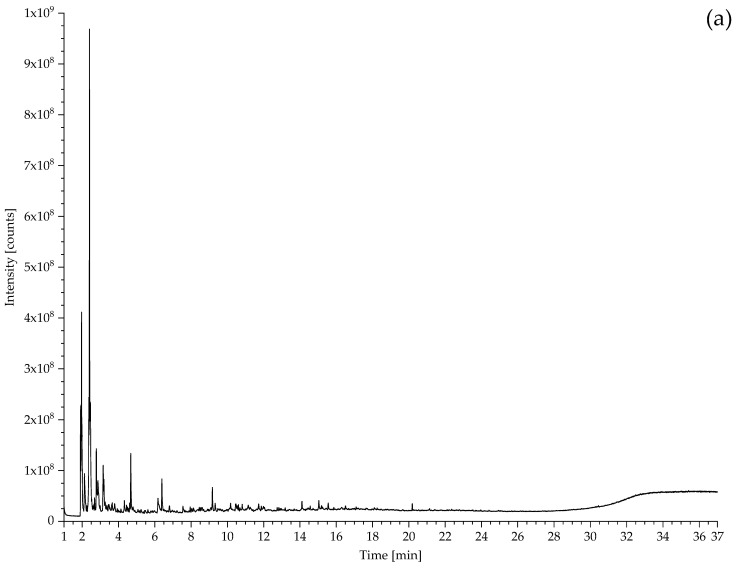

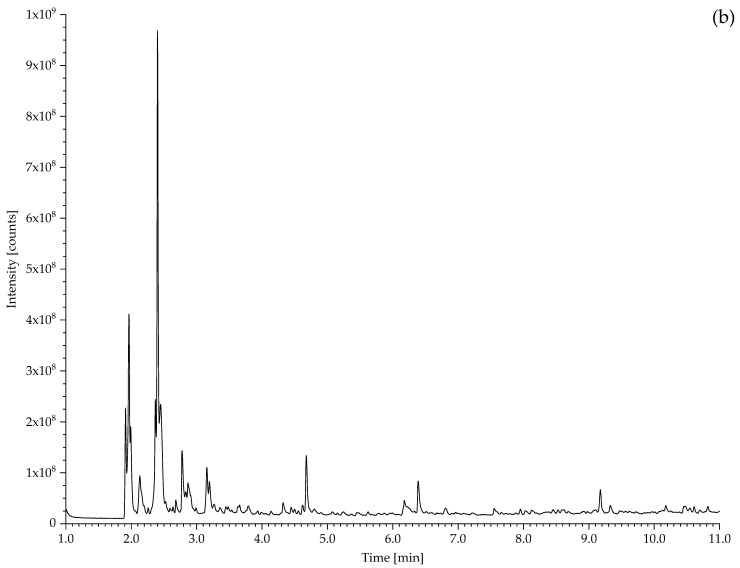
Pyrogram of sample 194 (**a**) and a zoomed-in view of the same pyrogram (**b**).

**Figure 7:**
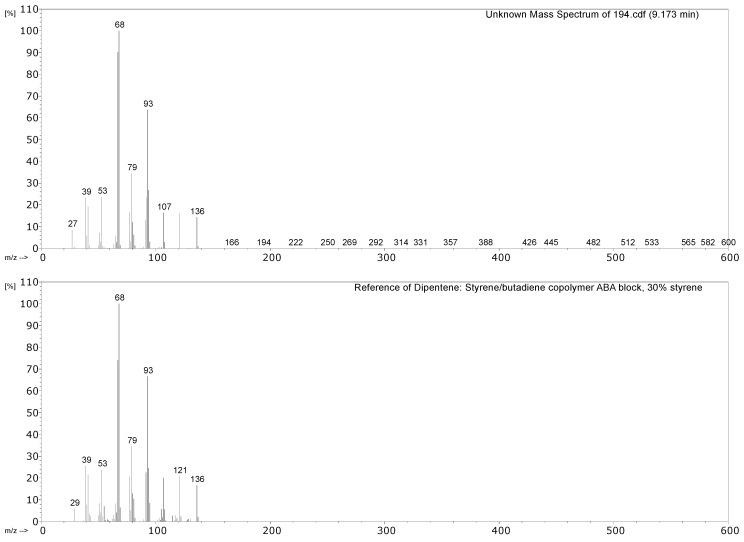
Mass spectrum of the substance eluting at 9.17 min, together with a reference spectrum from F-Search.

Sample 194 shows in its pyrogram (Figure 6) as main peak isoprene (2.40 min), accompanied by ethylene (1.91 min), toluene (4.68 min), a xylene (6.39 min) and dipentene (the racemate of limonene [65]) at 9.17 min, along with an isomer of the latter at 7.95 min, likely 2,4-dimethyl-4-vinylcyclohexene ([44], F-Search). A phthalate is present at 20.18 min. Particularly the presence of isoprene, dipentene (Figure 7) and its isomer 2,4-dimethyl-4-vinylcyclohexene, dimers of the monomer isoprene, are helpful for the interpretation, since they are characteristic of polyisoprene and are not present in the pyrograms of other rubbers at the used pyrolysis conditions. However, it was not possible to find the described trimers and tetramers typical of rubber [44]. 

In sample 194, the presence of further markers and monomers in smaller quantities cannot be ruled out. Other intense peaks are present at a lower retention time than isoprene; they may be ascribed to ethene, CO_2_ and propene. Particularly, the presence of propene may be indicating a more complex sample with at least a further polymeric component. 

The complexity of the pyrograms, both in the number of secondary peaks and their frequent coelution, makes it difficult to completely characterise all possible components of a rubber. Moreover, it is sometimes difficult to detect the presence of diagnostic, but similar monomers, simultaneously, which is the case of 1-butene and 1,3-butadiene. While their mass spectra are readily distinguishable, their close retention indexes (382 and 395, respectively) and the fact that their mass spectra share some peaks, makes it challenging to unequivocally detect the presence of the other, if one of them is present. A peak at 2.13 min in the pyrogram of sample 194, which may be assigned to butene, coelutes with a peak at 2.16 min which may correspond to 1,3-butadiene, as deconvolution analysis with AMDIS seems to indicate. The peak also shows masses at 64 and 48 *m*/*z* which could be indicating a small amount of SO_2_, hinting perhaps at a certain vulcanisation degree [66]. However, if 1,3-butadiene was present, 4-vinylcyclohexene, the dimer of butadiene, should appear in significant amounts in the used experimental conditions. This is nevertheless not the case, seemingly allowing to rule out the presence of BR in large quantities, as well as other polymers displaying 1,3-butadiene in its pyrogram, such as SBR. 

Propene and butene might be indicating the presence of further candidates, such as EPDM or polynorbornene (PN) [44], as well as a cyclohexadiene and methyldipentene at 3.16/3.19 min. However, an interpretation of these markers, as has been suggested, as PN for sample 194 is problematic. First, further known markers for PN could not be found (e.g., indene); second and most importantly, the first PN appeared at least two decades later in the market [67]. However, further markers for EPDM, particularly the alkene series typical of EPDM, could also not be found. Other polymers are also possible; a more detailed analysis goes beyond the scope of the present work section.

**Figure 8:**
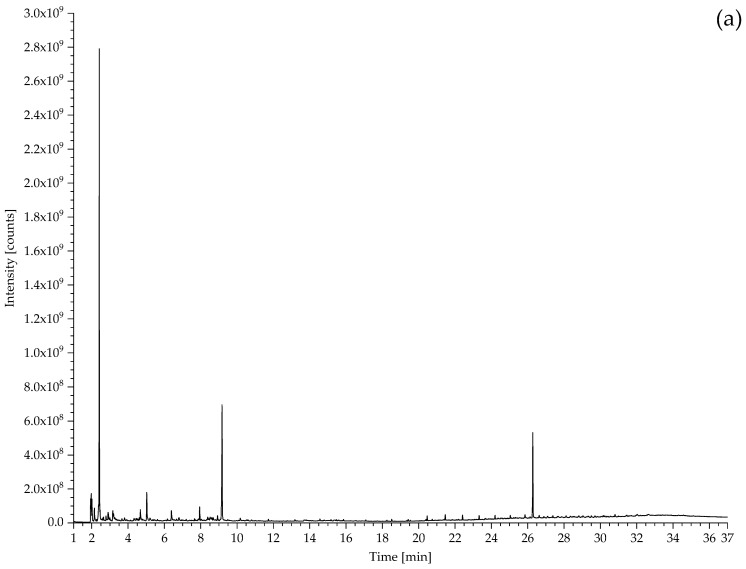

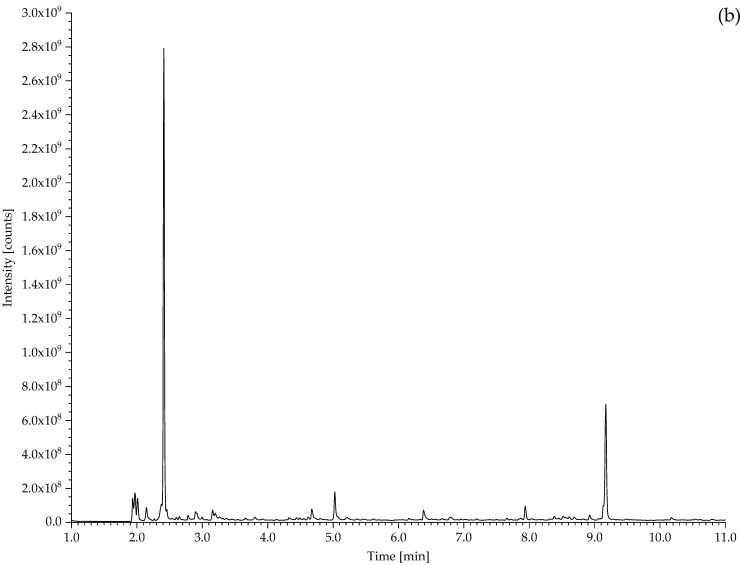
Pyrogram of sample 431 (**a**) and a zoomed-in view of the same pyrogram (**b**).

**Figure 9:**
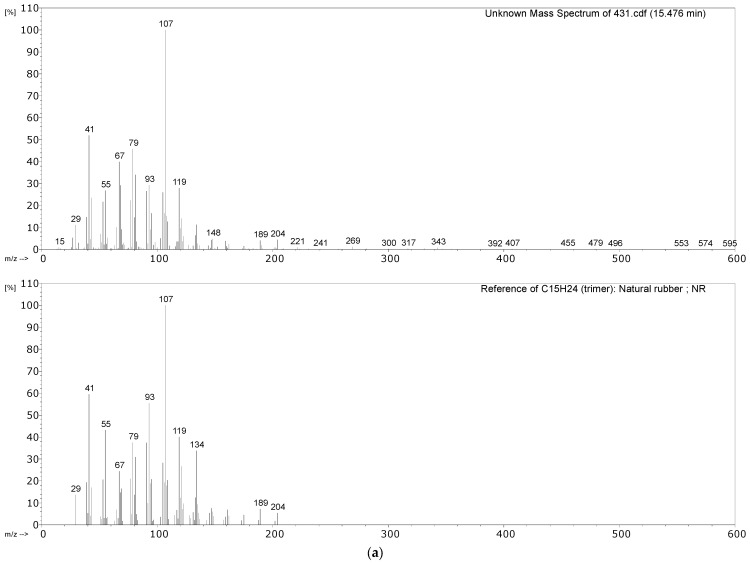

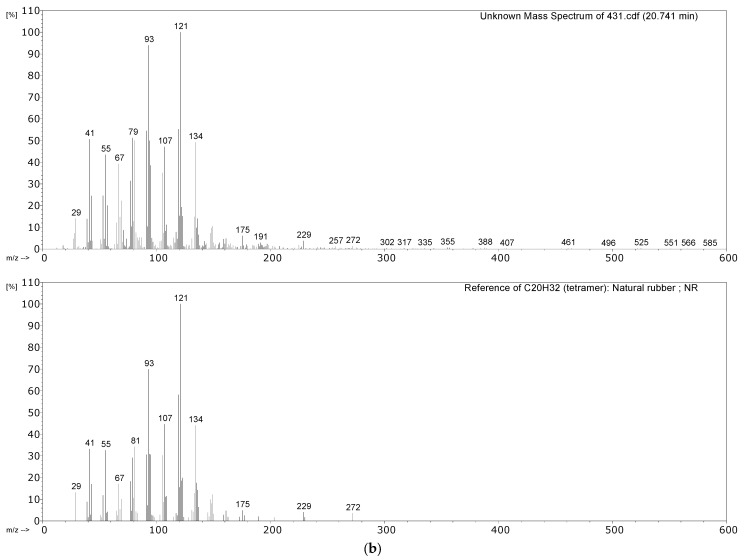
Mass spectra of some of (**a**) the trimers (15.48 min) and (**b**) tetramers (20.74 min) found, along with reference spectra (F-Search database).

In the case of object 431, the pyrogram showed peaks, which can be attributed to polyisoprene (Figure 8, isoprene at 2.41 min, toluene at 4.68 min, 2,4-dimethyl-4-vinylcyclohexene at 7.94 min, a xylene and dipentene at 9.17 min) as the main polymeric component, as well as bis(2-ethylhexyl) phthalate at 26.28 min. Further relevant peaks detected are 2-ethyl-1-hexene at 5.03 min, which can be attributed to the degradation of the plasticiser under the pyrolysis conditions used (F-Search). It could also be due to 1-polybutene; however, further markers would need to be present (e.g., 2,4-diethyl-6-methyl-1-octene), which could not be found in the pyrogram. The analysis of smaller peaks allowed the detection of a range of long chain alkenes (1.76 min: 1-hexene, 3.65 min: 1-heptene, 10.17 min: 1-undecene, also 11.72 min, 13.18 min, 14.56 min, 15.83 min). These peaks were not present in a separate thermodesorption analysis performed on the same sample, pointing to the macromolecular character of the material from which they originate. Together with the peak of propene at 2.01 min, they could be hinting to EPDM as a further minor polymeric component [44]. At high retention times, trimers (14.80, 15.17 and 15.48 min) and tetramers (20.74 min) of isoprene, characteristic of the pyrolysis of polyisoprene, could also be tentatively found in this case [44] and opposed to sample 194; their mass spectra can be consulted in Figure 9. 

**Figure 10:**
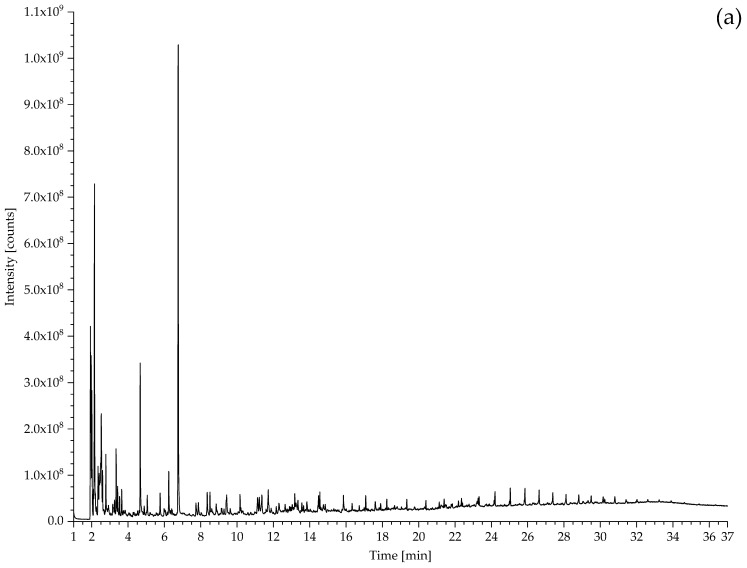

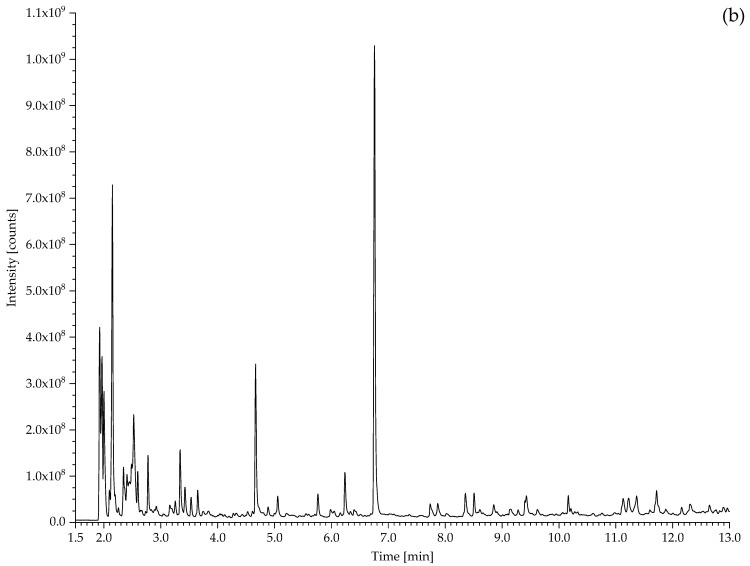
Pyrogram of sample 383 (**a**) and a zoomed-in view of the same pyrogram (**b**).

**Figure 11:**
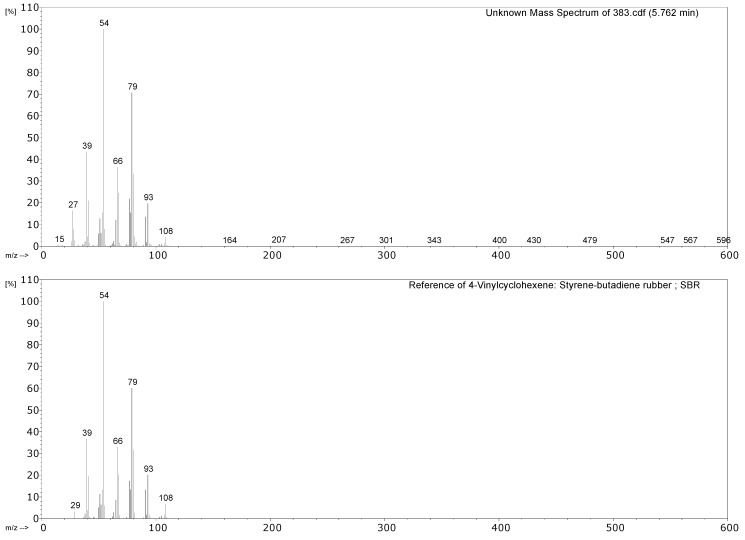
Mass spectrum of peak eluting at 5.76 min in the pyrogram of sample 383, along with a reference spectrum (F-Search database).

**Figure 12:**
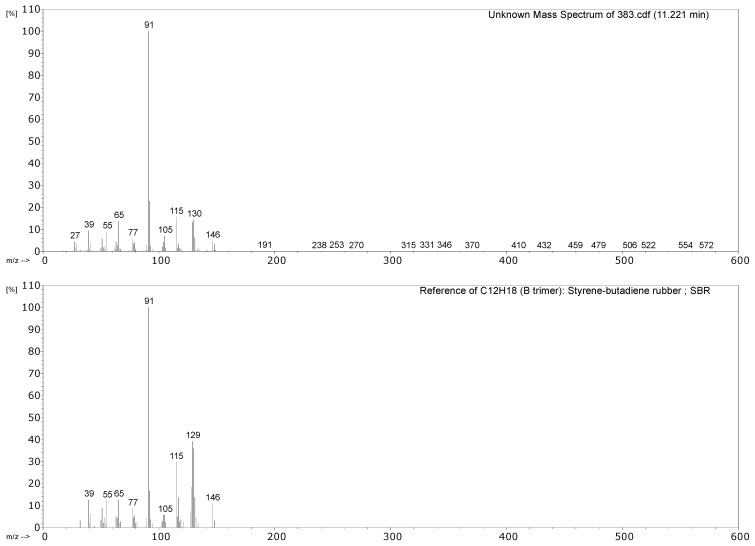
Mass spectrum of peak eluting at 11.22 min in the pyrogram of sample 383, along with a reference spectrum (F-Search database).

**Figure 13:**
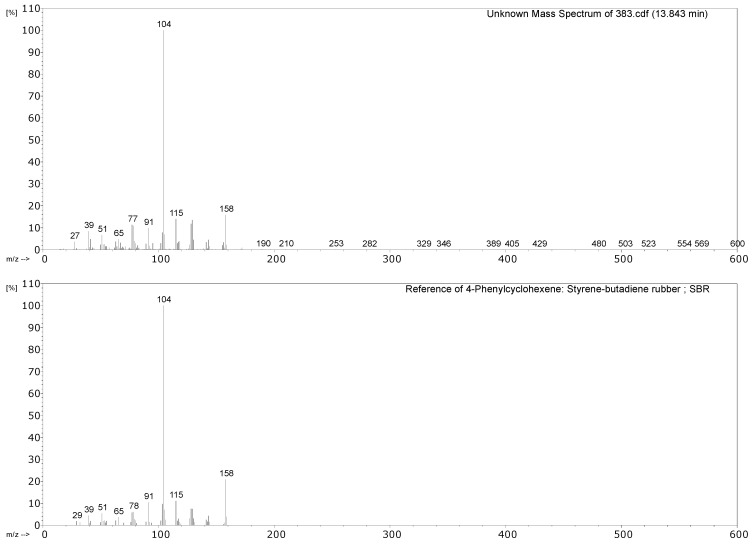
Mass spectrum of peak eluting at 13.84 min in the pyrogram of sample 530, along with a reference spectrum (F-Search database).

The py-GCMS analysis of sample 383 (Figure 10) allowed to confirm SBR as the main polymeric component, based on the main peaks at 6.76 min (styrene), 2.15 min (1,3-butadiene; probably coeluting with butene), 2.00 (propene) and 4.66 min (toluene). Further diagnostic pyrolysis products are 4-vinylcyclohexene at 5.76 min (Figure 11), xylene at 6.24 min, propylbenzene at 7.87 min, α-methylstyrene at 8.35 min, cyclopentadiene at 2.52 min, benzene at 3.34 min, biphenyl at 14.49 min and, particularly, the compound at 11.22 min, which has been described as a butadiene trimer (see mass spectrum in Figure 12) and a peak at 13.84 min, which coud be adscribed to 4-phenylcyclohexene, an SB (styrene-butadiene) hybrid dimer (Figure 13) (Tsuge book; Kusch, 2017). A peak corresponding to sulphur dioxide could be well recognised at 2.10 min. Further, smaller peaks could be attributed to a long chain series of alkenes and alkanes. Benzothiazole, which can be found at 12.30 min, probably appears as a pyrolysis product of a sulphur-containing additive such as the vulcanisation accelerator benzothiazole-2-thiol (2-MBT) [68].

**Figure 14:**
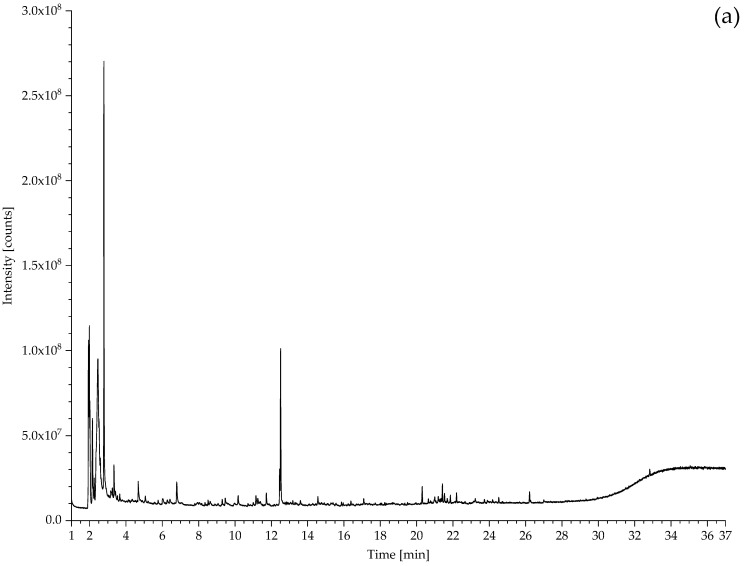

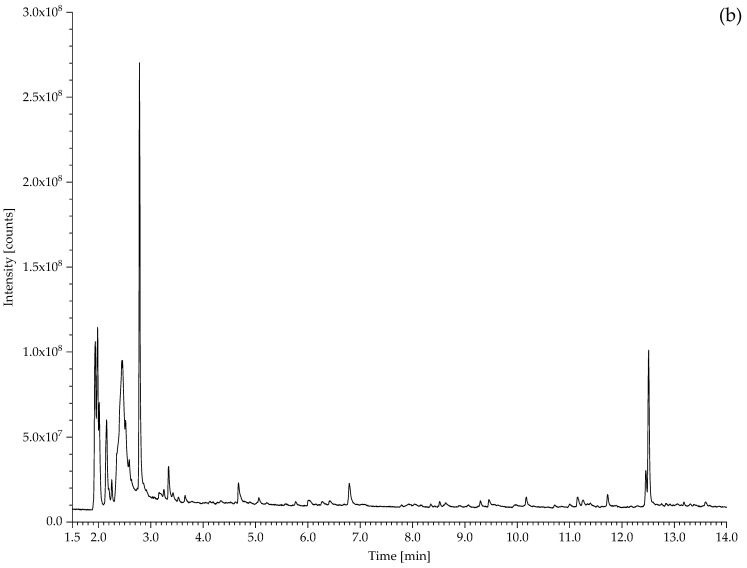
Pyrogram of sample 530 (**a**) and a zoomed-in view of the same pyrogram (**b**).

**Figure 15:**
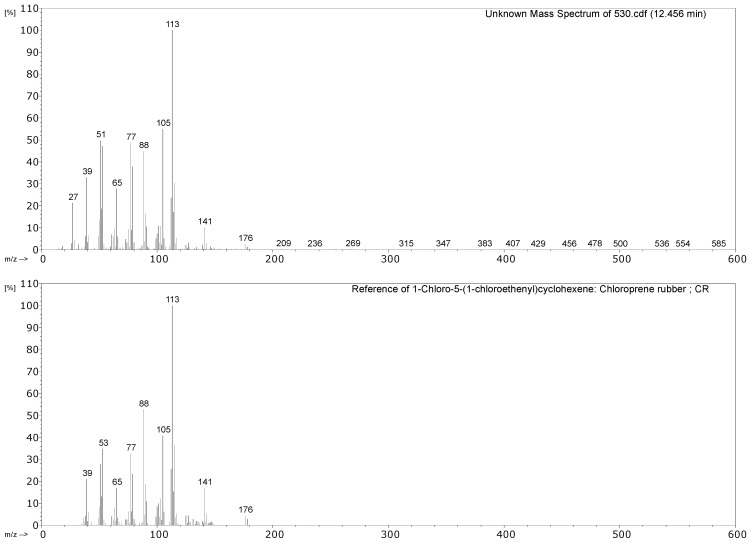
Mass spectrum of peak eluting at 12.45 min in the pyrogram of sample 530, along with a reference spectrum (F-Search database).

**Figure 16:**
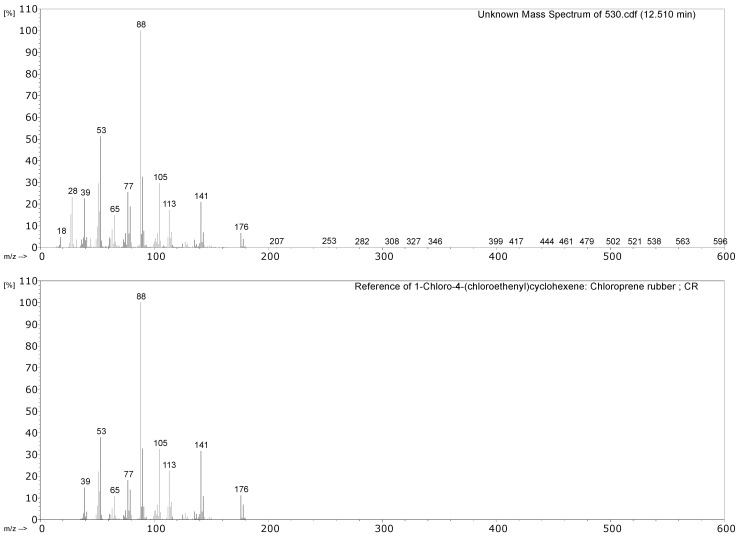
Mass spectrum of peak eluting at 12.51 min in the pyrogram of sample 530, along with a reference spectrum (F-Search database).

**Figure 17:**
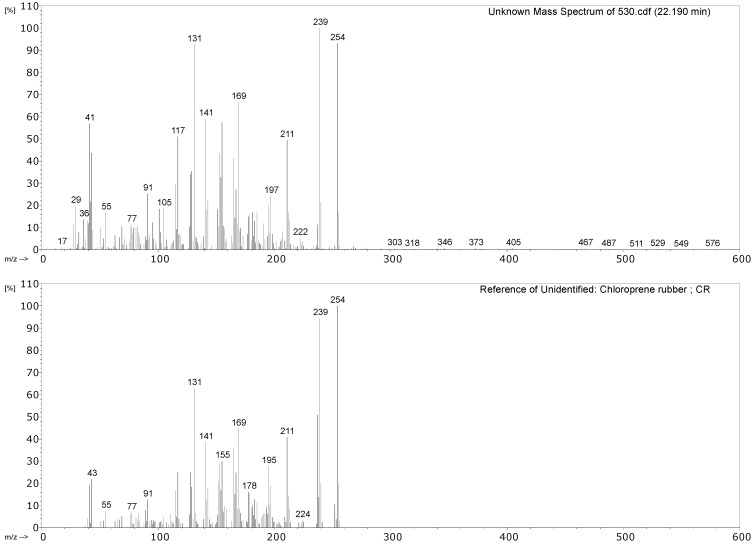
Mass spectrum of peak eluting at 22.19 min in the pyrogram of sample 530, along with a reference spectrum (F-Search database).

The pyrogram of sample 530 (Figure 14) is dominated by a large peak at 2.78 min, corresponding to 2-chloro-1,3-butadiene (chloroprene), a broad, unresolved region around 2.50 min which contains HCl and several (cyclo)pentadienes, a peak corresponding to 1,3-butadiene (and coeluting butene) at 2.15 min, and peaks for ethene (1.94 min), CO2 (1.98 min) and propene (2.01 min). Chloroprene, HCl and 1,3-butadiene, together with the peaks at 12.46 min [1-chloro-5-(1-chlorovinyl)cyclohexene), Figure 15] and 12.51 min [1-chloro-4-(1-chlorovinyl)cyclohexene, Figure 16], both dimers of chloroprene, are characteristic of the pyrolysis of CR. Further confirming for CR is the peak at 22.19 min (Figure 17), whose structure is not known but has been already described as characteristic of CR [44]. The presence of 4-vinylcyclohexene at 5.77 min, together with propene, 1,3-butadiene, pentadiene, and xylenes at 6.41 and 6.79 min indicate the presence of a further polymeric component related to poly(1,2-butadiene). Indane (9.29 min), indene (9.46 min), two methylindenes (at 11.15 and 11.25 min) and methylnaphthalene (13.58 min) also indicate further polymeric components, probably CM or CSM. This sample does not contain SO_2_. SO_2_ has been described as appearing in the pyrolysis of (unaged) vulcanised rubbers [66]; in fact, it could be found in most of the samples analysed here. This sample, and a further object which could also be identified as a neoprene (not described here), are the exception. Literature contemporaneous of this object describes that neoprene was not usually vulcanised with sulphur, instead zinc or magnesium oxides are used as curing agents [22] (p. 61); presently it is possible to vulcanise CR with sulphur [69]. At 21.68 and 23.22 min, the database F-Search allows to identify two components, which could be hinting towards the use of rosin or a derivative (metal soap of rosin acid) as an additive in the sample; these are known as stabilisers [25] (p. 289) or tackifiers [70] (p. 47), [71]. This additive, used since the beginning of rubber technology, has lost importance with time, because as a product of natural origin it is difficult to obtain with consistent quality.

**Figure 18:**
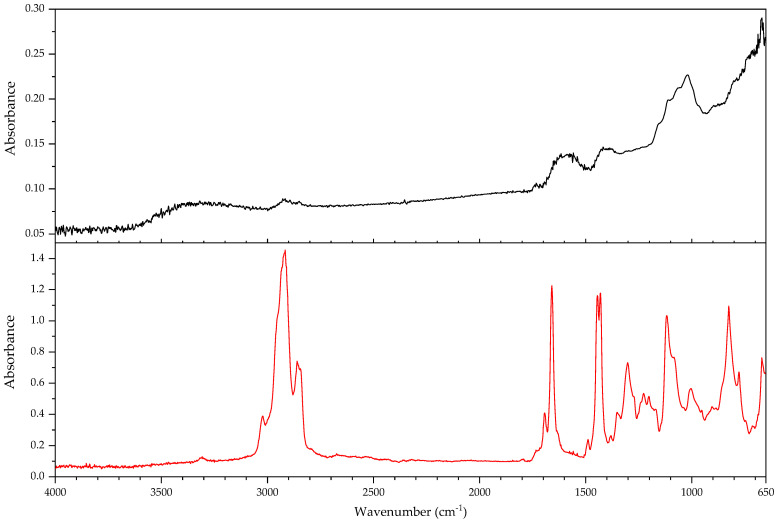
FTIR spectrum of sample 530 (black line) compared to reference spectrum of polychloroprene (red line) from a commercial database.

**Figure 19:**
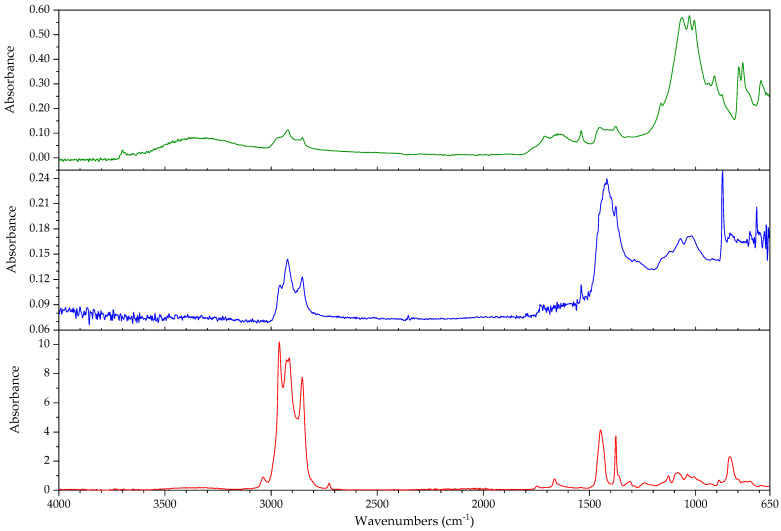
FTIR spectra of samples 194 (green line) and 431 (blue line) compared to reference spectrum of polyisoprene (red line) from a commercial database.

A comparison of the infrared spectra with reference data for the compounds identified in py-GCMS does not show a favourable correspondence. In the infrared spectrum of sample 530 (Figure 18) it was not possible to distinguish any of the bands characteristic of CR. In the spectra of samples 194 and 431 (Figure 19), it is possible to distinguish the asymmetric deformation band of the methyl group (–CH_3_) at 1374 cm^−1^ [72,73]. It should be mentioned that the spectra in databases are acquired from unaged reference material which, most importantly, is not compounded. While the above discussed factors (high filler content, presence of carbon black) surely play a major role in the differences between the spectra of the reference and of the analysed objects, the effects of ageing should also be taken into account.

These results show that the py-GCMS technique is almost indispensable for an in-depth understanding of the composition of the main polymeric components in elastomeric samples. Not only does it provide information on main and secondary monomers, but also of further polymeric components that may be present as a blend, and about additives, which may also help dating a given object. In turn, they also allow a better interpretation of the FTIR spectra acquired, also potentially contributing to the creation of a better spectral database for cases in which a sample cannot be taken. The fact that a very small sample size is enough to give enough information means that, even if not for all objects, it can be a practical solution for a significant amount of objects. 

However, the analysis of real, aged samples of rubbers by means of py-GCMS also presents a number of challenges. The pyrograms contain a fantastically high number of peaks, some of them minor but very diagnostic, many of them coeluting. Often, markers are missing, or not appearing in the expected intensity, raising the question of the role of ageing. The sheer amount of possible formulations, taking into account all possible copolymers, blends and additives throughout time, since the first rubbers were used, makes it essential to gather experience, not only from reference materials of pure compounds, but also from real samples, both aged and unaged. Here, artificial ageing may be of some help, if not a definitive solution. Further research is needed.

#### 3.2.2. Case Study: Blooming

In this work, blooming has been classified depending on whether its crystalline character is noticeable to the naked eye (‘crystalline blooming’) or not (‘blooming (other)’), in which case it presents itself as an amorphous deposit of matte, powdery or waxy aspect.

**Figure 20:**
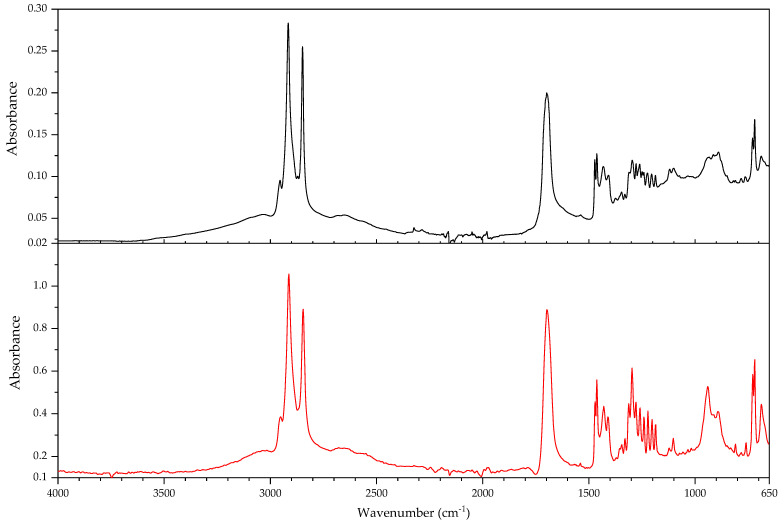
FTIR spectrum of sample 63 (black line) compared to reference spectrum of stearic acid (red line) from a commercial database.

In Table 11, some examples of blooming found during the survey are presented (see Supplementary Materials, Section S1), along with their classification as crystalline or not. Table 12 sums up the results obtained from the infrared analysis of the blooming samples and of the infrared and py-GCMS analysis of the plastics and rubbers on which they were found. For example, the blooming observed on the surface of sample 63, based on the absorption bands, can be ascribed to a fatty acid (probably stearic acid) (Figure 20). The bands at 2954 and 2870 cm^−1^ can be assigned to the asymmetric and symmetric stretching of the methyl group (CH_3_) respectively. While the peaks at 2918 and 2850 cm^−1^ can be attributed to the asymmetric and symmetric stretching of the methylene group (–CH_2_) respectively. Deformation peaks of the methylene group can be further observed at 1472 and 1462 cm^−1^. The stretching peak of the carbonyl group, –C=O, appears at 1698 cm^−1^. The band at 1431 cm^−1^ is due to the combination of the C–O stretching and O-H deformation vibrations. In the region between 1150 and 1350 cm^−1^ a series of peaks arise from the wagging and twisting vibrations of the methylene group (–CH_2_). Fatty acids are known to give rise to blooming in historical paints [74]; typically, azelaic acid has been found. The pyrogram of a sample of the coating on which the blooming was found, shows peaks that, due to the small sample and coelution, in some cases could only be tentatively assigned to propene, butene, butadiene and pentene. Due to the extremely low amounts available of the sample for py-GCMS analysis, it was difficult to detect minor but diagnostic components. Among these, cyclopentadiene, cyclopentene, benzene, cyclohexene, cycloheptanone, several methylated alkenes, a phthalate, a range of long-chain alkenes, a marker for propylene glycol monostearate (F-Search) and, interestingly, stearic acid (peak at 23.07 min) could be found.

**Figure 21:**
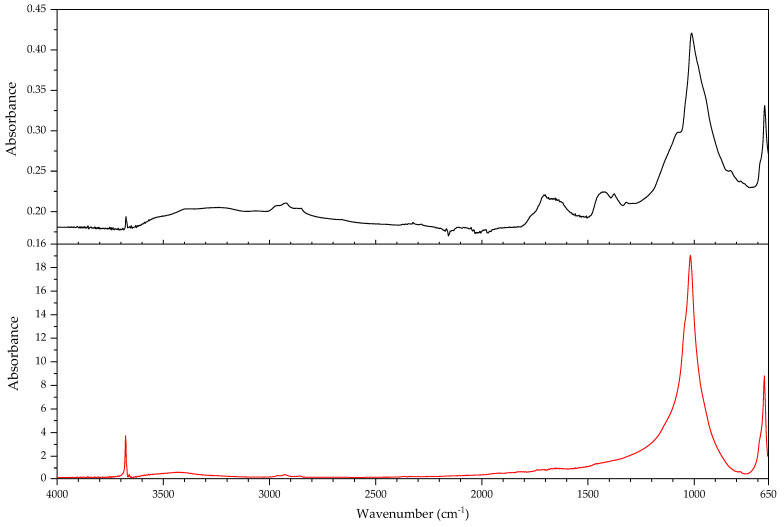
FTIR spectrum of the blooming of object 579 (black line) compared to reference spectrum of talc (red line) from a commercial database.

**Figure 22:**
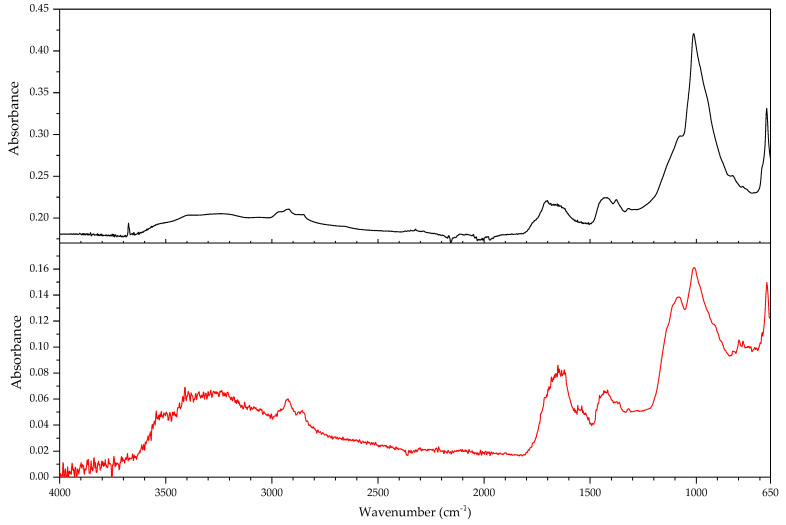
FTIR spectrum of the blooming of object 579 (black line) compared to the FTIR spectrum of the rubber material on which it was found (red line).

Talc (hydrated magnesium silicate), a common inorganic filler, was identified on the surface of sample 579 by ATR-FTIR analysis (Figure 21). Characteristic bands can be observed at 3675, 1012, 667, 464, 449, 441 and 421 cm^−1^ [72,73]. In the pyrogram of the sample, only butene can be noticed, due to the small sample size. However, the object function allows to suspect an originally elastomeric material (now brittle). Talc is well known as an additive in rubbers, as a reinforcement agent [75]. In fact, Figure 22 shows that the rubber in which it was found also contains talc (the measured area was black and did not contain the blooming).

**Figure 23:**
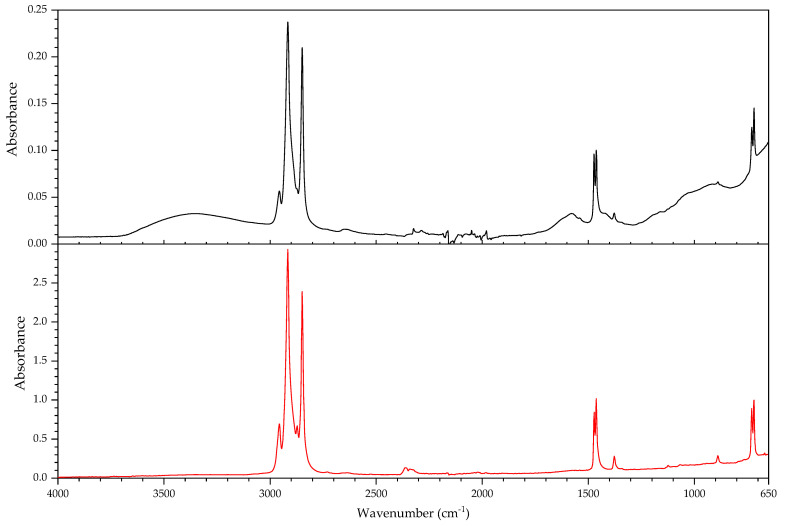
FTIR spectrum of the blooming of object 594 (black line) compared to reference spectrum of a mineral wax (paraffin, red line) from a commercial database.

**Figure 24:**
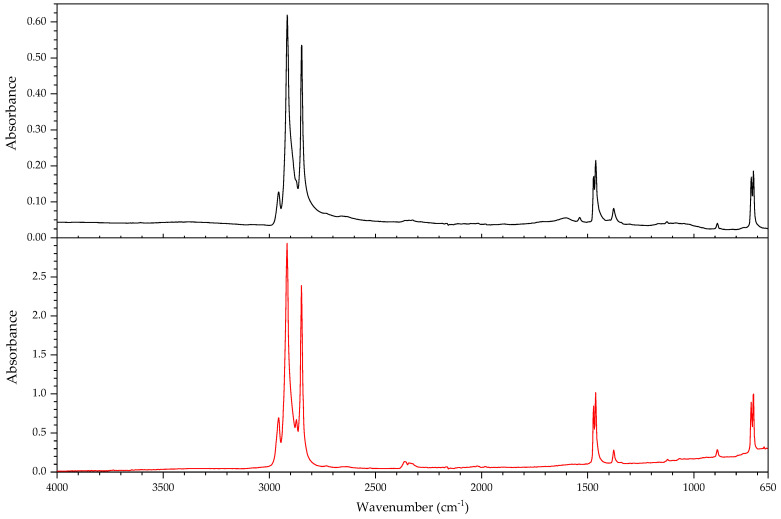
FTIR spectrum of the blooming of sample 2223-2 (black line) compared to reference spectrum of a mineral wax (paraffin, red line) from a commercial database.

For sample 594 and 2223-2 the same type of blooming can be determined, a mineral wax. The spectra exhibit typical adsorption bands of a paraffin or microcrystalline wax (Figure 23 and Figure 24). The two strong and sharp bands at 2917 and 2849 cm^−1^ can be assigned to the asymmetric and symmetric stretching vibration of the methylene group (–CH_2_–) respectively. While the asymmetric and symmetric stretching vibration of the methyl group (–CH_3_) can be observed at 2954 and 2870 cm^−1^. The peaks at 1463 and 1377 cm^−1^ may correspond to the asymmetric and symmetric bending vibrations of the methyl group (–CH_3_) respectively. The peak at 1473 cm^−1^ can be attributed to the bending vibration of the C–H bond in the methylene group (–CH_2_–). The sharp doublets at 730 and 720 cm^−1^ are characteristic for the in-plane rocking vibration of the methylene group (–CH_2_–) in a semi-crystalline structure and indicate the presence of at least four methylene groups in the chain (–(CH_2_)_n_–CH_3_, n ≥ 4) [72,73,76,77,78]. The GCMS analysis of a sample of the blooming showed a series of non-branched alkanes, leading to the conclusion that the wax is a paraffin [79]. Waxes are typically used in rubber materials as a protective agent against ozone [79]; a wax protects by migrating to the surface and causing an intended blooming. In fact, in the analysed rubber parts (see pictures in Table 11), the blooming appears as a rather homogeneous, widespread layer. In this sense, this phenomenon is not a degradation and its removal would have a deleterious effect in the ageing of the object. 

**Figure 25:**
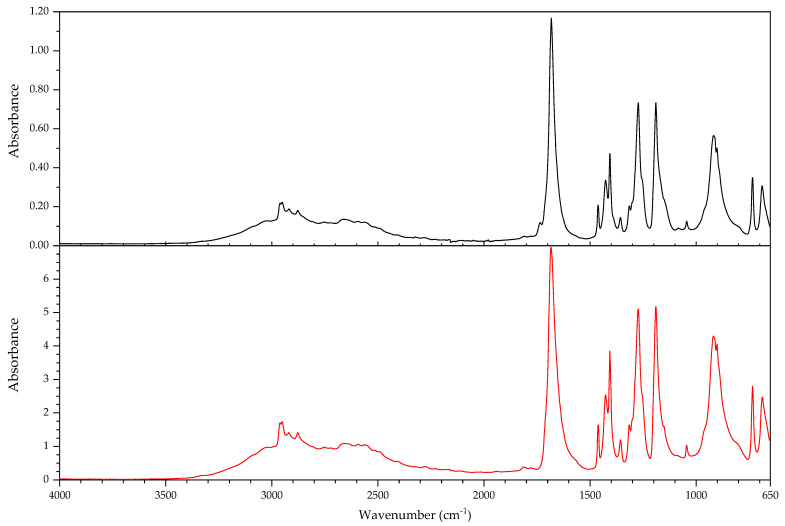
FTIR spectrum of the blooming of object 610 (black line) compared to reference spectrum of adipic acid (red line) from a commercial database.

The spectrum of sample 610 shows the presence of adipic acid (hexanedioic acid), a typical degradation product of adipate ester-based polyurethane (Figure 25). The main absorption bands at 1683, 1273, 1190, 918 and 734 cm^−1^ confirm this [80,81]. According to Suzuki and Schimanouchi’s article [82], the bands can be assigned as follows. The absorption bands at 2961, 2952, 2919 and 2878 cm^−1^ can be attributed to asymmetrical and symmetrical stretching of the C–H bond. The carbonyl bond can be observed in the peak at 1683 cm^−1^. The bending vibration of the C-H bond in the methylene group (–CH_2_–) can be assigned through the bands at 1462 and 1407 cm^−1^. The peak at 1273 cm^−1^ can be ascribed to the stretching vibration of the C–O bond while the deformation vibration of the C–C–O and O–C–O bond can be observed at 688 and 513 cm^−1^ respectively. Together with the peak at 1356 cm^−1^, the strong band at 1190 cm^−1^ can be assigned to the wagging vibration of the methylene (–CH_2_–) group. The peak at 734 cm^−1^ can be ascribed to the rocking vibration of the –CH_2_– bond. Finally the peaks at 1083, 1044 and 918 cm^−1^ describe the stretching vibration of the C–C backbone.

## 4. Conclusions

In accordance with the first aim of this paper, this article presents a comprehensive, descriptive and commented atlas of unique degradation phenomena in plastics. The descriptions are supplemented with pictures so as to facilitate their identification. The atlas hopes to be particularly useful, but not exclusively, for scientific, technological and industrial collections. Often, the first use of a given polymer or plastic, or a significant development, was first carried out and applied in an industrial environment, and with a technical purpose. Science and technology museums, similarly to many design, arts and crafts museums, gather technically mature and established plastic formulations representative of the time in which they were produced, and in a way, housing the history of plastics and macromolecular science from their beginnings in the form of historical materials. In the case of modern art museums, for example, the use of one material or another depends rather on the needs and preferences of the artist, and does not necessarily obey the intended purpose of the manufacturer [16]. In contrast, technical and industrial heritage museums may offer the chance of gathering a more representative number of damage phenomena for a given plastic, for example, by systematically collecting related objects throughout time. In this sense, an atlas based on these materials can be a useful basis and complement the information of existing atlases. 

In the atlas, particular care has been subjected to the delimitation of related terms and to offering as broad as necessary a list of occurring damage phenomena, while at the same time keeping it as succinct as possible, so as to avoid making it unpractical to use. It was decided to prioritise an *economy of terms*: in museum surveys, scarce resources need to be optimised, and a long evaluation time per object means fewer objects can be inspected. In this respect, it aids museums to work sustainably. 

A strength of damage atlases lies at being able to provide the user with an intuitive set of terms that can be applied fast and precisely in the frame of a survey. In a survey, *consistency* is critical; if intended to deliver information about the progress of degradation state, the same set of descriptions or definitions must be kept throughout time. This work attempts to provide a consistent glossary of unique terms in order to avoid redundancy and aid consistency. 

The approach presented here focuses on a purely descriptive evaluation using what have been called “observational terms”, *independently of the causes* that may be behind the damage observed, with the purpose of allowing objectivity to prime over interpretation. As far as possible, in the description of the damage phenomena, references to their possible origins have been deliberately and consequently left out. Objectivity needs to prime over interpretation in order to ensure a consequent use of survey databases, today and in the future, if the information is to document the progress of degradation in collections.

For some or perhaps many of the terms described here, there is no right or wrong; rather, the attempt has been made to remain consequent and avoid the overlapping of terms as much as possible. For this, a comprehensive discussion of the chosen descriptions as based in the literature search is offered in this work. In this respect, the present atlas may be a starting point for a survey in a collection or museum context and be modified according to that collection’s requirements, as based on the discussions gathered above. In fact, it has been reported that when staff carrying out surveys use guides they feel comfortable with, the survey leads to more reliable condition assessment results [7]. Therefore, this work does not aim at substituting existing atlases of damage phenomena in plastics, but rather hopes to continue the scientific discussion started by previous authors. 

The documentation of damages is often the first step in the development of a preservation strategy. Additionally, it enables a more in-depth study of degradation phenomena [3] and their origin and mechanisms by documenting cases and therefore allowing for recognising correlations. Nevertheless, the description of a phenomenon may reach its limit when different processes are simultaneously involved. 

Regarding the second aim of this work, analytical techniques have been successfully implemented for the study of materials and ageing processes in historical synthetic polymers. On the one hand, ATR-FTIR is a well-known powerful technique for the identification of the main components of plastics. It allows a fast, user-friendly, non- or minimally invasive analysis leading to the identification of the main components (provided a good, reliable reference database and an experienced user is available) that is particularly suited for use in museum surveys. However, its effectiveness is sometimes limited when dealing with materials such as rubbers and phenol formaldehyde resins e.g., Bakelite^®^: highly compounded materials, often with highly absorbing carbon black, overlap diagnostic bands of the polymeric components, making it difficult to even be able to classify the kind of polymer present.

On the other hand, and particularly for the case of rubbers, py-GCMS has proved to be an extremely useful technique for the minimally invasive identification of the main monomers present in the bulk, providing often also detailed information about co-monomers and additives. Here, the complementary use of FTIR and py-GCMS can lead not only to a better identification of a given material, but also aid in building material and analytical databases of naturally aged polymeric materials. For this, a large number of relevant materials need to be unequivocally analysed and documented. On the downside, py-GCMS is a time-consuming and expensive technique regarding the maintenance of the instrument. The interpretation of the results requires experience with real, aged samples with complex formulations, and knowledge in polymer technology and the range of copolymers, blends and additives that have been used throughout time since the first rubbers were used.

Finally, the study of several cases of blooming has shown once more the diverse compositions that they may present, illustrating that the description of a phenomenon is only the first step. A detailed analysis is necessary in order to better understand the ongoing changes in the material, particularly when conservation treatments, such as the removal of a (potentially protecting) layer, are planned. For this purpose, ATR-FTIR as a surface technique is particularly useful and can provide an answer in many cases.

Overall, the documentation and analysis of damage phenomena in historical polymers and their changes with time can unlock a wealth of information on the natural ageing of polymers, which can in the long term enable a more sustainable relationship with these materials at an industrial and societal level. Further research is needed in the analysis of damage phenomena, in order to better understand their causes and be able to plan treatments and preventive conservation interventions.

## Figures and Tables

**Table 5:** Definitions of the subcategory ‘loss of integrity usually involving loss of material’.

Loss of Integrity Usually Involving Loss of Material		
Abrasion	(a) Concentration of thin, shallow cuts or small chips and scratches, which may show up as a roughening of the surface with concomitant gloss reduction, appearing usually in (a) particular direction(s)	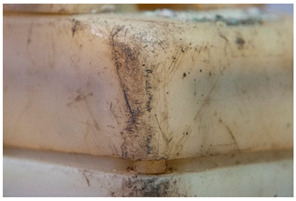
	or (b) smoothening of a surface caused by the repeated friction against another surface, accompanied with gloss increase.	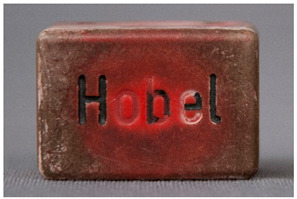
Chip	Punctual loss of surface material, typically a spalled rim. In material with glass-like behaviour, a conchoidal fracture may appear (also: spalling)	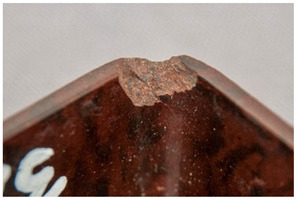
Crumbling	Detachment of material due to loss of coherence, characterised by extensive friability that usually starts in the surface and advances into the depth of the material. It can appear, e.g., in the form of cube break, or, in the case of foams, lead to powder in its final stages.	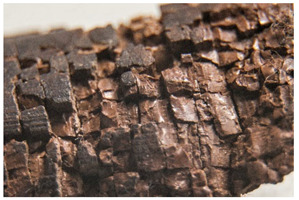
Flaking	Usually applied to the detachment of material in the form of small flat platelets.	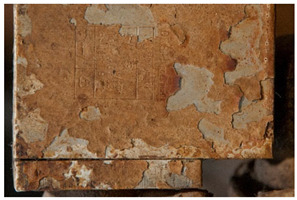
Loss of material (other)	Loss of a constituting part of an object, as caused by a different damage phenomenon than abrasion, chip, crumbling, flaking or scratch.	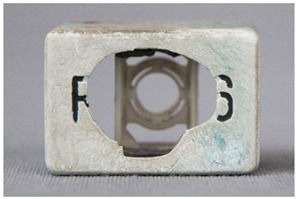
Scratch	Rather linear, occasional or isolated thin incision(s) or scrape(s) on the surface, regardless of its depth; extensive accumulation of scratches in a given direction is rather an <abrasion>.	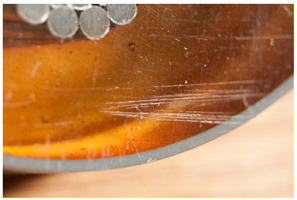

**Table 6:** Definitions of the subcategory ‘loss of integrity not necessarily involving loss of material’.

Loss of Integrity not Necessarily Involving Loss of Material		
Brittleness	Behaviour of materials through which they are likely to crack or break when subjected to pressure.	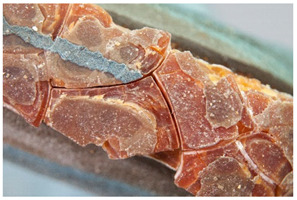
Break	Complete crack running through material, with separation of two or more pieces. For films and sheets, the term ‘tear’ is used; fracture is another descriptor.	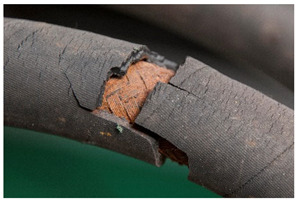
Crack	Small fissure or opening which does not cause separation of an object part (cf. break).	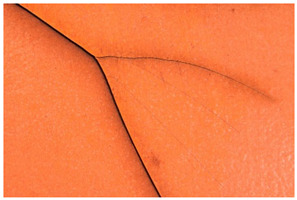
Craquelure	Superficial network of cracks or fissures affecting the outmost layers of a material, such as is typically found in varnishes.	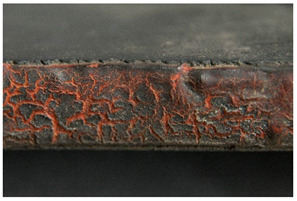
Crazing	Crazing may refer to (a) internal micro-crack region, macroscopically visible as stress whitening with possible loss of transparency.	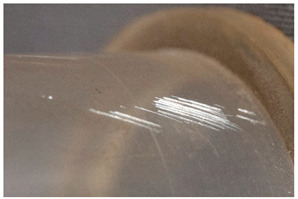
	or (b) network of fine cracks starting in the surface or within a material, which spreads throughout it. (Case b) typically affects cellulose nitrate and may be accompanied by concomitant whitening (loss of transparency).	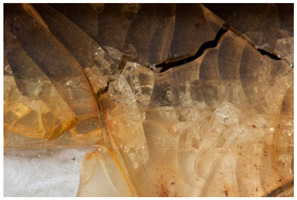
Hardening	Loss of elasticity and plasticity, (partial) loss of the ability to recover its form and of the ability to change it without break after being submitted to stress.	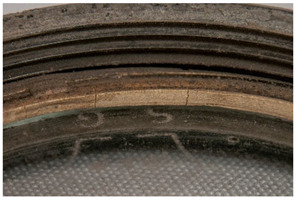
Loose	Partial detachment of a constituting part of an object, with risk of breakage or loss of material.	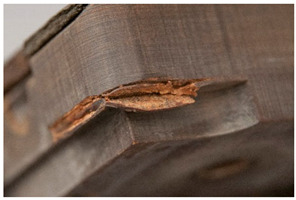
Peeling	Detachment of a relatively large coating, foil or other layer from the underlying surface, sometimes accompanied by curling of the detached part; delamination.	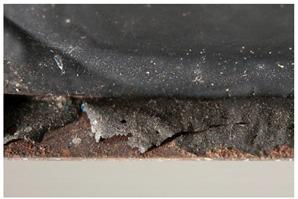

**Table 7:** Definitions of the category ‘smell’.

Smell	
Acrid smell	Acrid till pungent smell of certain substances (e.g., acid, ammonia, formaldehyde) and which cannot be described otherwise (e.g., as vinegar smell).
Camphor smell	Aromatic and fresh, menthol-like smell.
Naphthalene smell	Aromatic odour of the organic substance naphthalene, reminding of old, 20th century mothballs.
Rubber smell	A typical rubber smell can be noticed in tyres or cheap rubber products.
Vinegar smell	Typical smell of vinegar coming from acetic acid appearing as a degradation product.

**Table 8:** Definitions of the category ‘other/miscellaneous features’.

Other/Miscellaneous Features		
Adhesive tape	Repairs or fixations with self-adhesive or pressure-sensitive (‘sticky’) tape or similar products. Also meant by this term are residues or damage (e.g., stains, sticky areas) left and caused by these, and which are easily recognisable as such.	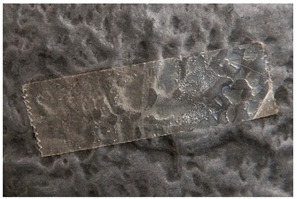
Biological attack	Any sign of mould or moss growth, or insect, bird or mammal attack, as evidenced by certain discolouration signs, fruiting bodies, excrements, feeding/scuff marks and galleries.	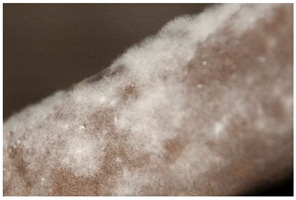
Corrosion (metal part)	Products of the degradation of metal elements near plastic parts.	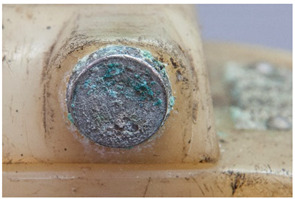
Overpaint	Colour layer that has subsequently been applied over an original surface.	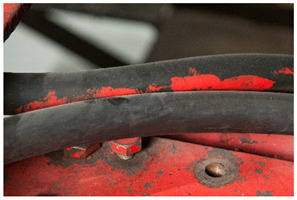
Sticky	Adhesive or gluey character of a surface; behaviour of a surface through which it stays attached to other surfaces that it touches.	

**Table 9:** Overview of some rubber objects found during the survey. The numbering used for the samples during the survey are also used here as the ‘sample number’ for orientation throughout the text.

Sample Number, Object, Part, Approximate Production Date		Photo of Object and Object Part
194 Object: miner‘s bathing slippers Part: belt 1950s	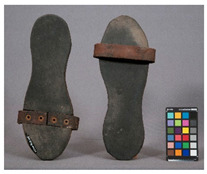	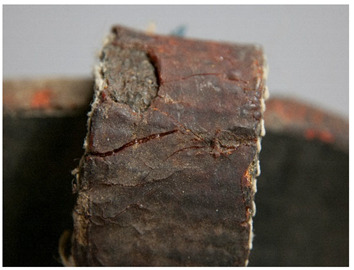
383 Object: Dosco Roadway Cutter Loader (roadheader) Part: supply hose for water nozzle 1960s	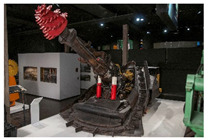	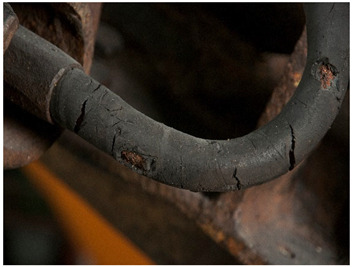
431 Object: diesel-hydraulic Trolley of a monorail system Part: hose sheath of centrifugal governor 1970s	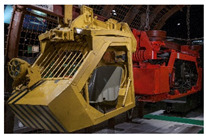	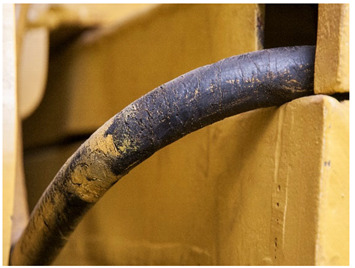
530 Object: air-powered monorail trolley Part: hoses around 1980	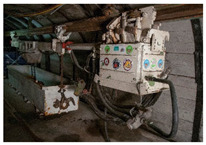	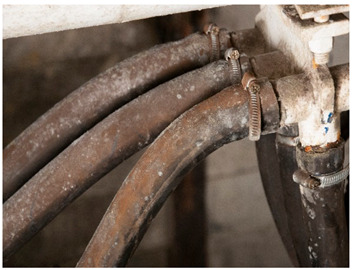
598 Object: methanometer Part: pillow-shaped air bulb/rubber bulb probably between 1950s–1980s	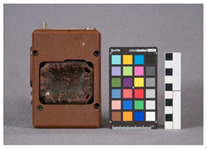	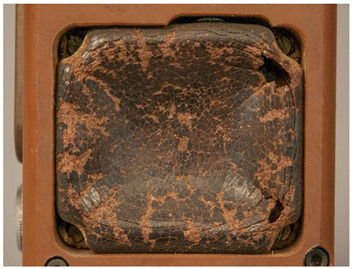

**Table 10:** Summary of the py-GCMS results.

Object/Part	Main py-GCMS Peaks (Major Peaks are Underlined)	Main Polymeric Component
194 Drive belt from the model of a coal cutting machine	CO_2_, propene, SO_2_, isoprene, toluene, xylene, dipentene, 2,4-dimethyl-4-vinylcyclohexene.	NR, vulcanised with sulphur.
383 Hose for water nozzle	CO_2_, propene, SO_2_, 1,3-butadiene, benzene, toluene, xylene, 4-vinylcyclohexene, styrene, α-methylstyrene, benzothiazole.	Styrene containing elastomer (probably SBR), vulcanised with sulphur.
431 Hose sheath	Propene, SO_2_, isoprene, xylene, dipentene, 2,4-dimethyl-4-vinylcyclohexene, long chain alkenes, bis(2-ethylhexyl) phthalate (plasticiser).	NR, vulcanised with sulphur. Minor EPDM component?
530 Hose of air-powered monorail trolley	1,3-butadiene, HCl, 2-chloro-1,3-butadiene (chloroprene), 4-vinylcyclohexene, indane, indene, 1-chloro-4-(1-chlorovinyl)cyclohexene.	Chloroprene as main component; markers of poly(1,2-butadiene), CM and CSM also present.

**Table 11:** Blooming samples. B(c): Blooming (crystalline), B(o): Blooming (other). The internal survey numbers for samples are used here as the ‘sample number’ for orientation throughout the text. Photos: Till Krieg, except gas mask: Florian Pohlmann.

Sample Number, Object, Part, Approximate Production Date and Sample Name	Photos of Object and Object Part	
63 Object: calculator Part: coating of casing 1920s Sample: LackGehäuseteile 63 B B(c)	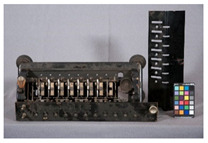	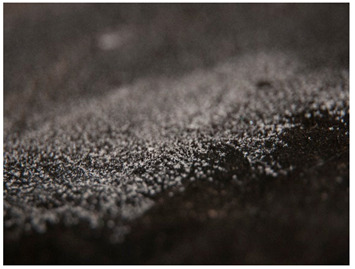
579 Object: smoke helmet Part: inner sealing band 1930s Samples: Innendichtband 579 A B(o)	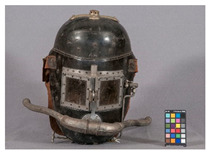	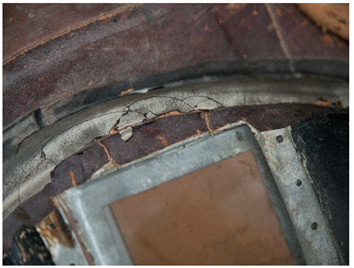
594 Object: inclined tube manometer Part: stopper 2nd half 20th century Sample: Stopfen 594 B B(c)	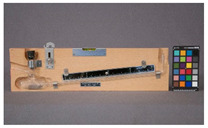	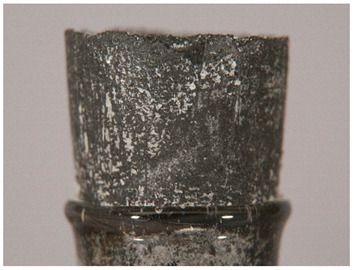
610 Object: mining hammer Part: pressure handle 1970s Sample: Ballendrücker 610 B B(o)	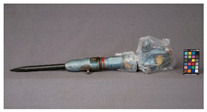	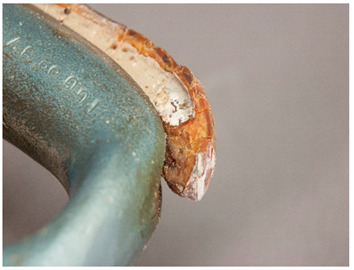
2223-2 Object: gas mask Part: headband 1980s Sample: Atemschutzmaske 2223-2 B(o)	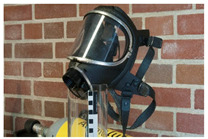	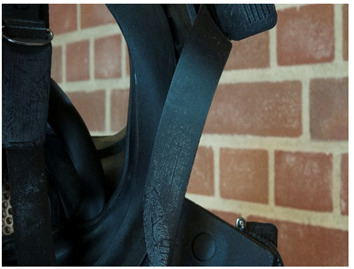

**Table 12:** Summary of the analytical results of the blooming samples and of the plastics on which they were found.

Object/Part	Chemical Nature of the Plastic on Which It Was Found	Nature of the Blooming
63 Coating of a calculator casing	Unknown	Fatty acid(s)
579 Inner sealing band of a smoke helmet	Rubber (butene-based?)	Talc
594 Stopper	Polyisoprene (main component), contains styrene as a secondary monomer	Mineral wax
610 Pressure handle of a mining hammer	Polyester urethane	Adipic acid
2223-2 Gas mask	Chloroprene-containing	Mineral wax

## Supplementary Materials

The following materials are available online at https://www.mdpi.com/article/10.3390/polym14010121/s1, Section S1: The starting point. Surveying plastic objects at the Deutsches Bergbau-Museum Bochum, Section S2: Visual atlas of damage phenomena, Table S1: List of degradation phenomena in alphabetical order, with photos of examples (Figures S1–S85) taken during the museum survey that motivated this work.
