# Supplementary material for: Material Analysis and a Visual Guide of Degradation Phenomena in Historical Synthetic Polymers as Tools to Follow Ageing Processes in Industrial Heritage Collections
Source: Polymers (Basel). 2021 Dec 29;14(1):121. doi: 10.3390/polym14010121 (PMC8747559; doi:10.3390/polym14010121)
Supplement: Supplementary file 1 [file polymers-14-00121-s001.zip › polymers-1185884-supplementary.pdf]

Supplementary Information

S1. The starting point. Surveying plastic objects at the Deutsches Bergbau-Museum Bochum

During 2019 and 2020, a survey of objects with plastic parts was carried out at the Deutsches Bergbau-Museum Bochum (DBM). The museum collection focuses on Technical and Industrial Heritage and contains, among others, technical appliances, machines and protective equipment that were used underground and above ground, in addition to art and cultural objects related to mining and metallurgy.

The survey was performed in a selection of objects with a materials science and conservation point of view. After inspection of the damages and documentation of the present conservation state, relevant materials were analysed by means of a handheld ATR-FTIR spectrometer. The collected information was documented into a dedicated database[1] that includes a comprehensive damage catalogue. The interpretation of the collected data about material and degradation phenomena reveals the conservation needs of modern materials in the collection. Based on this information, appropriate treatment can be designed and storage conditions reconsidered.

The project aimed at answering the following questions: What kind of plastics are present in the collection and how widespread are they? In which condition are they? Is their condition stable? Where are the malignant plastics? How many objects are definitively lost because of too far advanced damage processes?

To answer these questions, the surveying of as many different kinds of objects and plastics materials as possible was prioritised. It should be noted that the survey was not based on a statistical sampling method. Rather, the cross-section of objects chosen is *qualitatively* representative of the collection, as opposed to *quantitatively* representative, in so far that the chosen sample does not represent the distribution of objects nor of materials in frequency. To reach the goal of *qualitative* representativeness,

[1] The database is based on Access and presents different layers, from less to more specific. It starts with a list of objects, from which the information for each object can be viewed in the next layer. The third layer gathers the information of each plastic part including damage phenomena noticed; each damage is evaluated in a scale from 0 (damage phenomenon not present) to 4 (the presence of the damage phenomenon is widespread and jeopardises the understanding of the object by the museum visitors). The fourth layer presents information on the material analysis of each sample or analysis made on each plastic part.

objects from each thematic area of the museum were included in the survey (e.g. safety gear, miners' lamps, quarrying). Within each thematic area, where possible, at least one object of each different available decade was included in the survey. Both time and accessibility limited the amount of objects surveyed, the latter related to the recent renovation of the museum.

During the twelve months the survey lasted, 133 objects were assessed, amounting to a total 698 plastic parts. This makes an average of around five plastic parts per object. In total, around 4.800 photographs were taken for the documentation of the objects. On average, each plastic part was analysed twice non-invasively by means of the handheld ATR-FTIR. With the purpose of further studying degradation phenomena, as well as those parts where the lack of contact with the ATR crystal hindered the acquisition of a good spectrum, sampling was performed where possible.[2]

The infrared analysis has shed light on the material distribution of a representative sample of the collection. To date, 38 different types of plastics have been found.[3] Among the most common materials were rubbers of different composition, PVC (particularly plasticised), polystyrene, polyethylene, ABS, PMMA, polyamide and polyurethane, as well as cellulose nitrate as coating. In a significant number of spectra, the main organic material could not be directly identified based on the acquired infrared spectra. In this group, rubbers and resins such as the formaldehyde resin Bakelite® are likely present to a large extent. The difficulty in the interpretation of the infrared spectra of these materials can be both due to changes in the infrared spectrum appearing upon degradation, and/or to the fact that both rubbers and formaldehyde resins are typically compounded with a high amount of fillers, which in formaldehyde resins can make up to between 20-80% of the final material [6] (pp. 1079). Rubbers were the most challenging group to identify; some of the analytical challenges encountered with rubbers in general are discussed in the section *Identification of rubbers* (see main text). The fraction of malignant plastics was estimated to be around 50% of all plastic parts (excluding cellulose nitrate coatings).

Regarding their state of conservation, according to the current data, around 15% of the surveyed plastic parts were in a

[2] From an ethical point of view, museum objects should ideally not be sampled, and every intervention in this respect weighed the advantages of the information gained through it against the loss caused.
[3] The results of the material analyses carried out in the frame of the survey will be published elsewhere.

poor, very poor or 'unacceptable' (no longer deemed fit for exhibition) condition. Over 66% of the surveyed plastic parts were in a very good or good condition.[4] *Quantitatively*, this number does not necessarily describe the current overall conservation state of the plastic materials in the collection. Rather, this number is at least partially a consequence of the kind of *qualitatively* representative survey made, and may therefore be reflecting the relatively high stability of most existing plastics. This stability is the origin of the environmental issues these plastics arise, particularly regarding microplastic pollution.

In order to analyse damage processes and perform early interventions, a periodic monitoring is necessary. In this respect, the database can be understood as a tool for future condition assessment. By consulting the database, curators and conservators of other mining collections may also be able to gain better knowledge of their own objects.

S2. Visual atlas of damage phenomena

Table S1. List of degradation phenomena in alphabetical order, with photos of examples

**Abrasion**	a) Concentration of thin, shallow cuts or small chips and scratches, which may show up as a roughening of the surface with concomitant gloss reduction, appearing usually in (a) particular direction(s); or b) smoothening of a surface caused by the repeated friction against another surface, accompanied by gloss increase.

[4] The remaining 19% were in a ‚fair' condition. The different categories (from 'very good' to 'unacceptable') were defined and definitions documented in the database. This should allow a long term consistency.

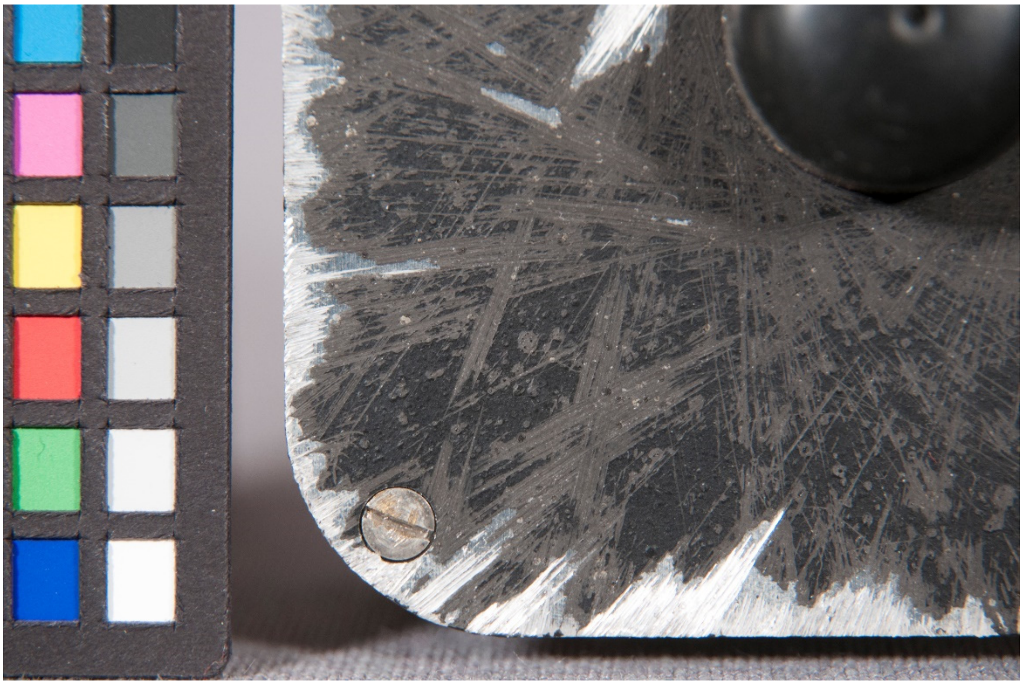

Figure S1. Radially abraded paint layer; locally concentrated scratches (abrasion, type a).

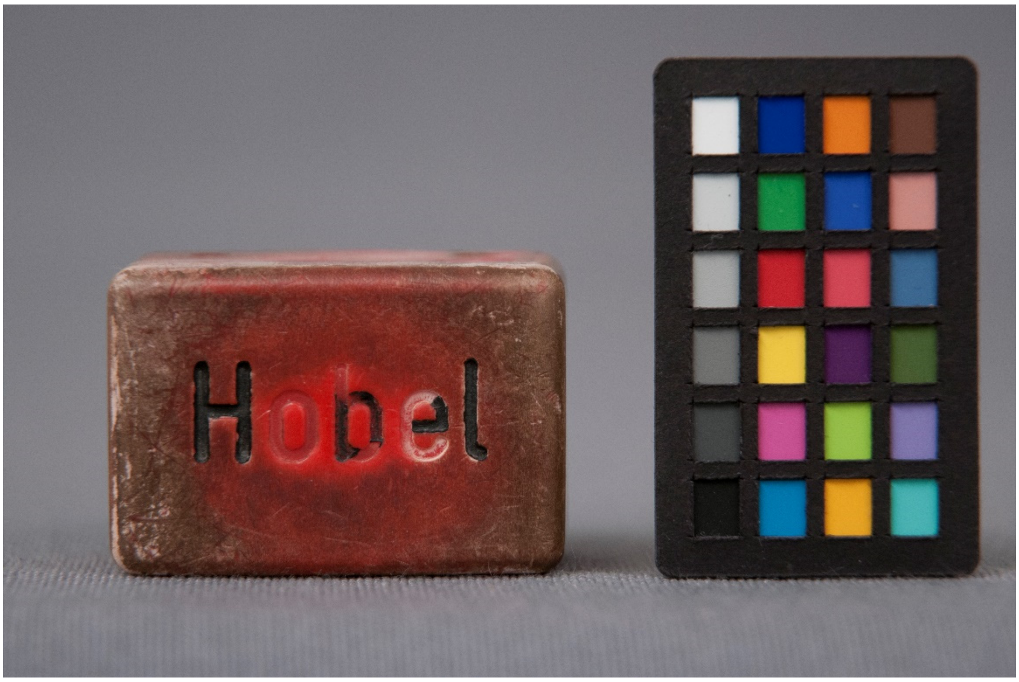

Figure S2. Button made of polyoxymethylene; increased gloss due to frequent and regular abrasion of the material in the contact area (abrasion, type b).

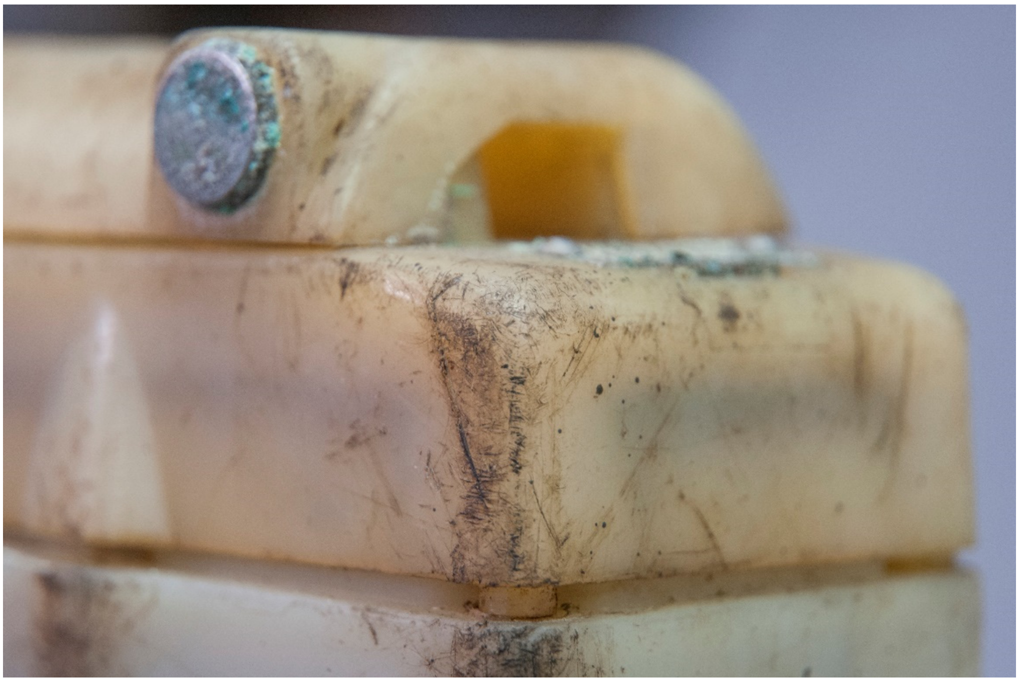

Figure S3. Scratched and bumped edge area of a miner's lamp case, abraded due to repeated use. Made of polyamide.

**Acrid smell**	Acrid till pungent smell of certain substances (e.g. acid, ammonia, formaldehyde) and which cannot be described otherwise (e.g. as vinegar smell).
**Adhesive tape**	Repairs or fixations with self-adhesive or pressure-sensitive ('sticky') tape or similar products. Also meant by this term are residues or damage (e.g. stains, sticky areas) left and caused by these, and which are easily recognisable as such.

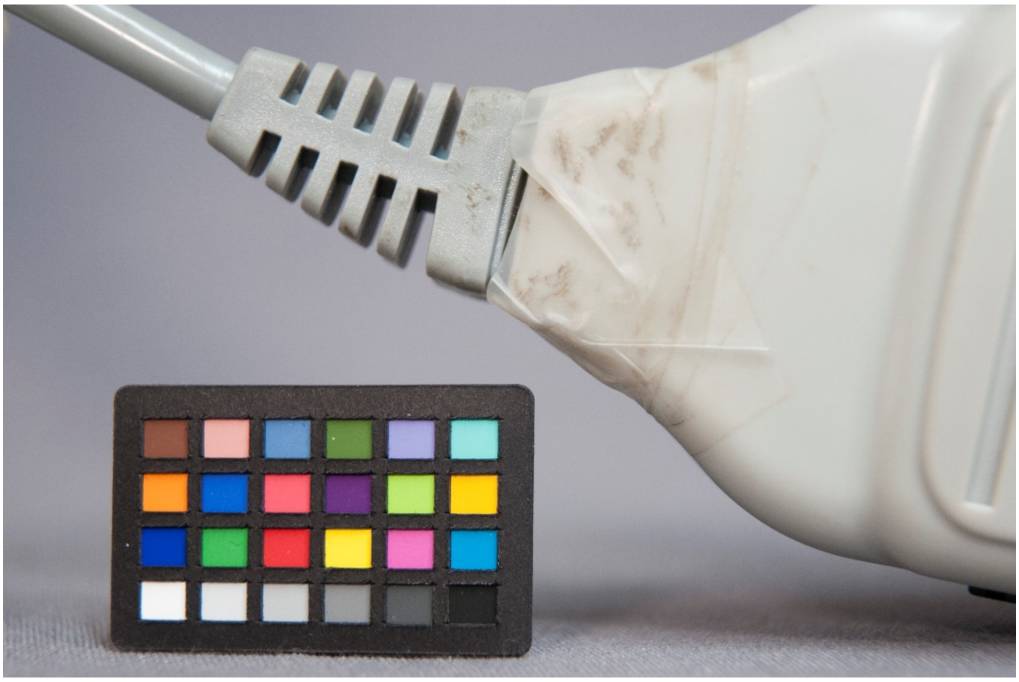

Figure S4. Defect microphone case made of acrylonitrile butadiene styrene, 'fixed' with pressure sensitive tape (matte finish tape).

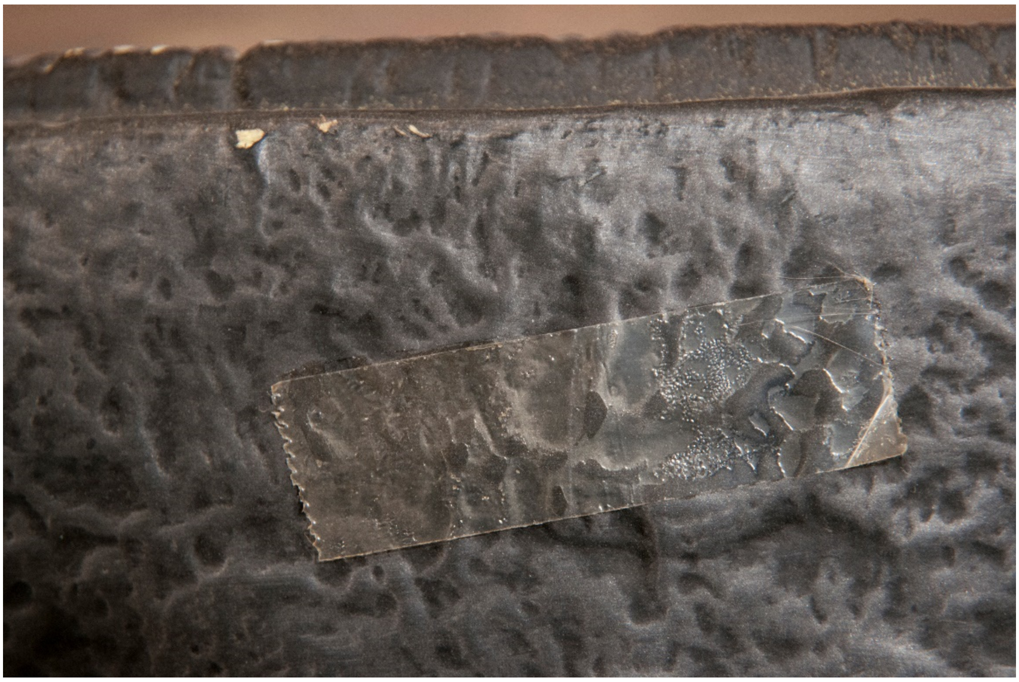

Figure S5. Strip of adhesive tape with visible 'wet' effect of the adhesive.

**Biological attack**	Any sign of mould or moss growth, or insect, bird or mammal attack, as evidenced by certain discolouration signs, fruiting bodies, excrements, feeding / scuff marks and galleries.

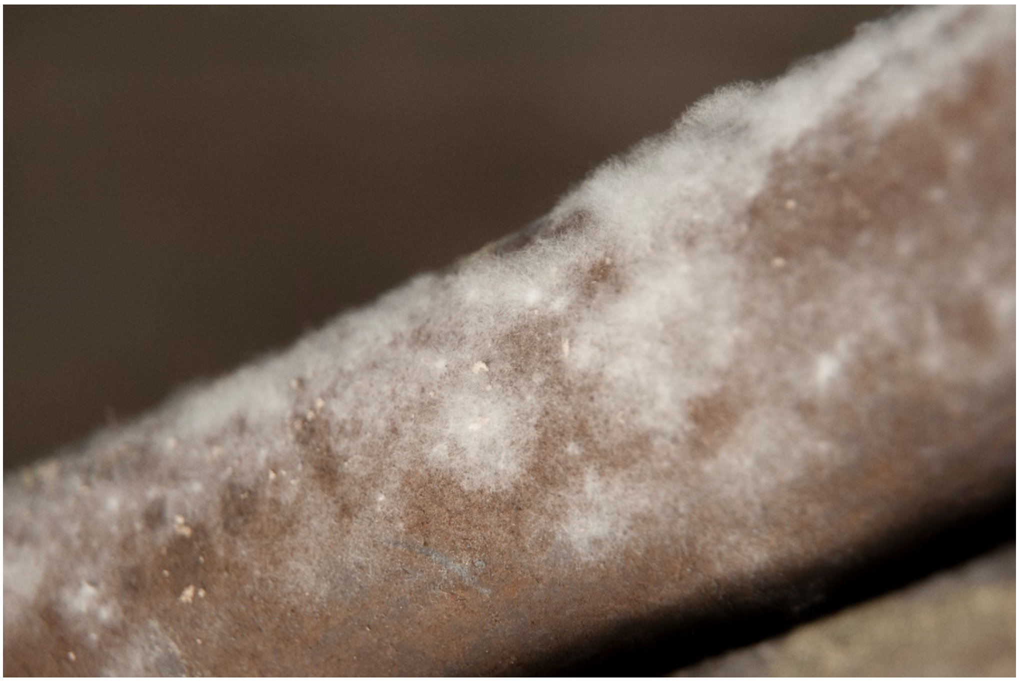

Figure S6. Mould on a pneumatic hose made of polychloroprene, kept in a very damp environment where humidity condensate on surfaces.

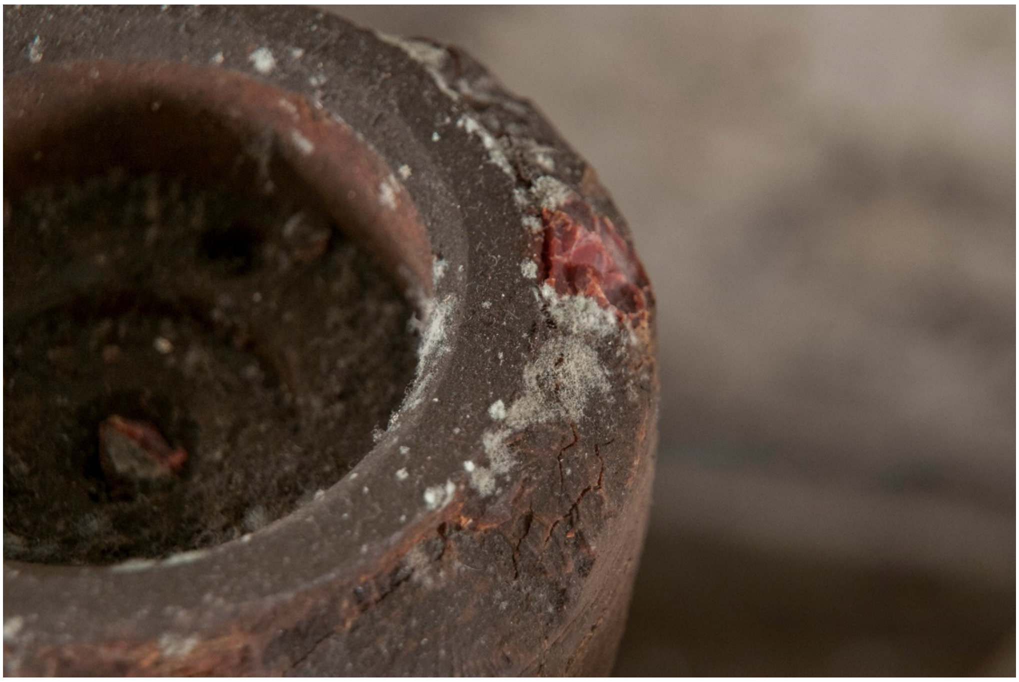

Figure S7. Mould on a friction drive (wheel) of a monorail system made of polyester urethane.

**Blistering**	Raised area, bulge or bubble on an objects' surface, often between adjoining layers of different materials.

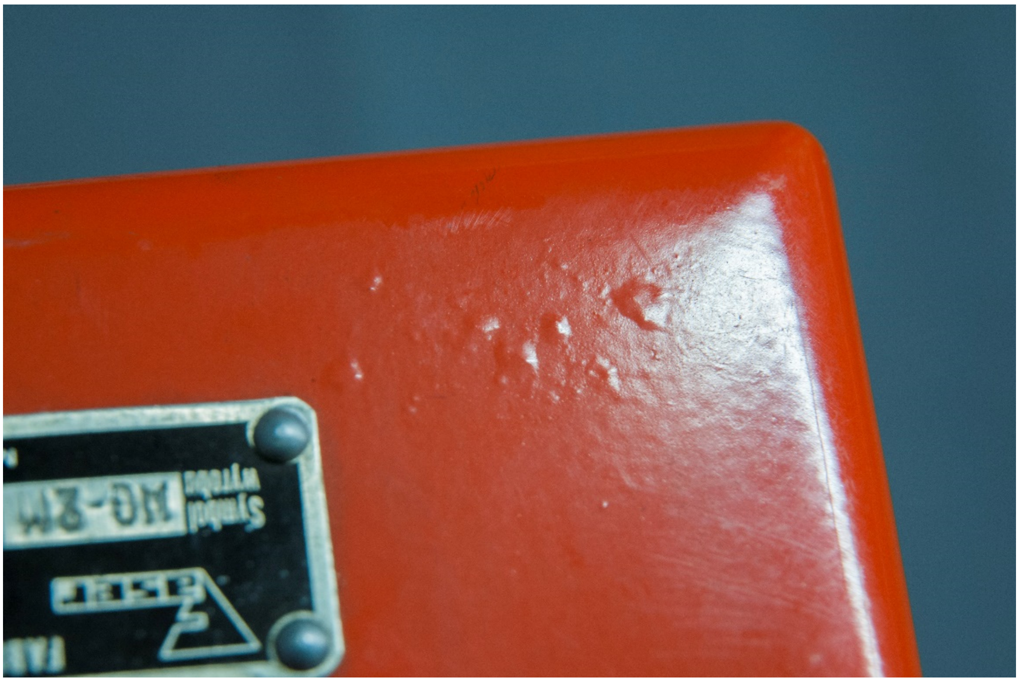

Figure S8. Blisters in an orange paint (epoxy resin based) of a metal box.

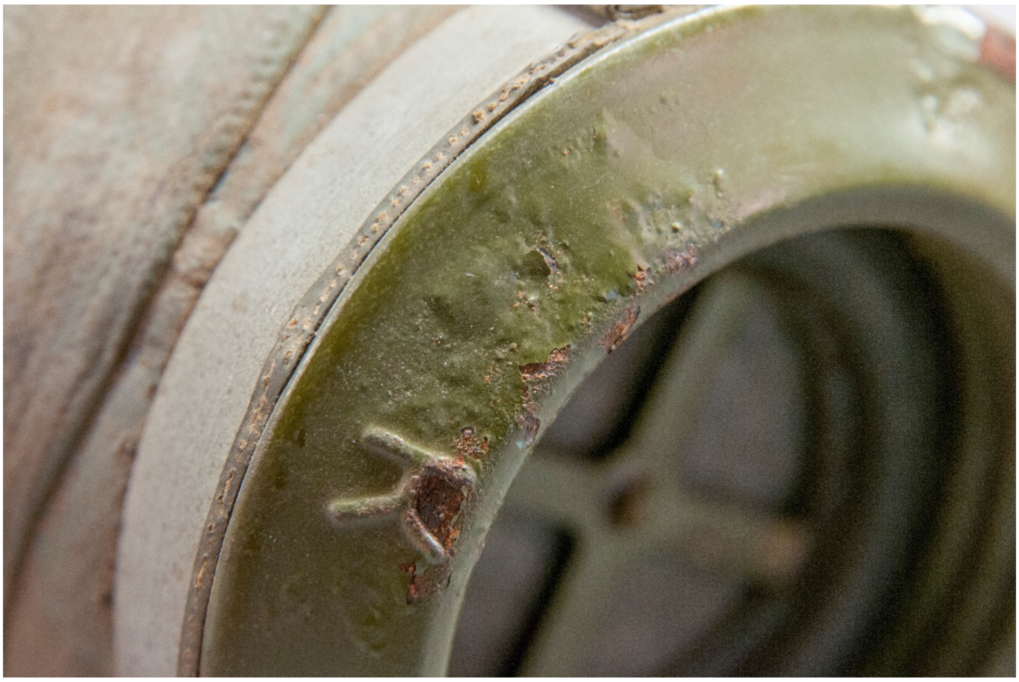

Figure S9. Blistering in the green paint layer of the filter connector of a gas mask from the 1930s.

**Blooming (crystalline)**	Crystalline efflorescence, where the crystalline character is identifiable macroscopically, e.g. through the identification of small crystalline particles and/or, depending on light incidence, a shiny reflection. Typically white.

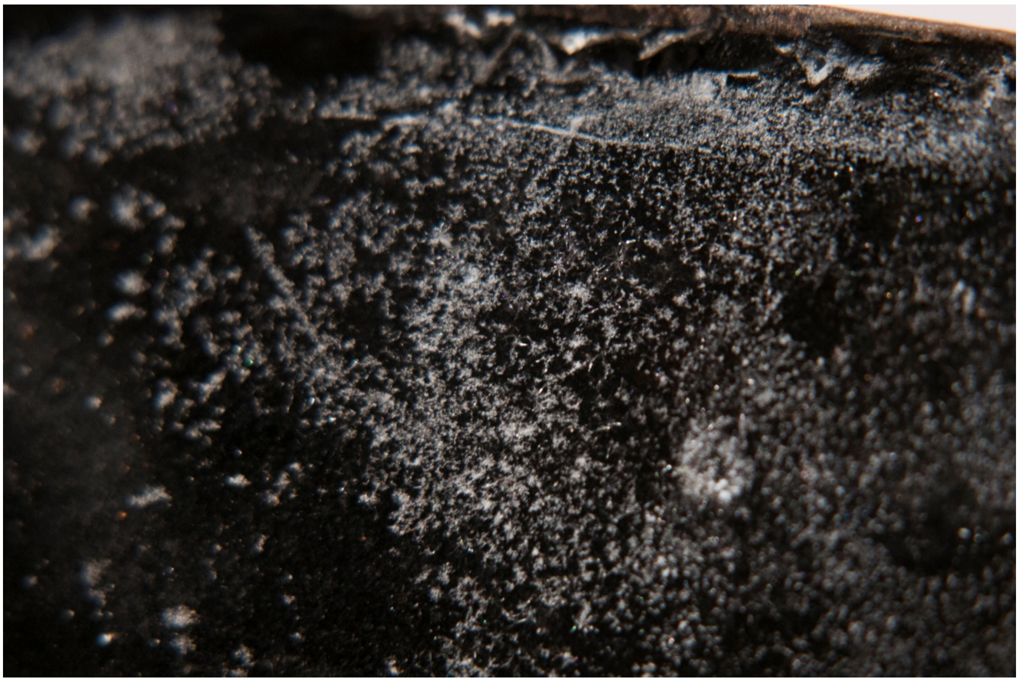

Figure S10. Fatty acids in form of white crystalline efflorescence (crystalline blooming) on the black coating of a calculator from the 1920s.

**Blooming (other)**	Amorphous deposit, macroscopically non-crystalline, of matte, powdery or waxy aspect, or (cloudy) film affecting large surface areas. Typically whitish-greyish.

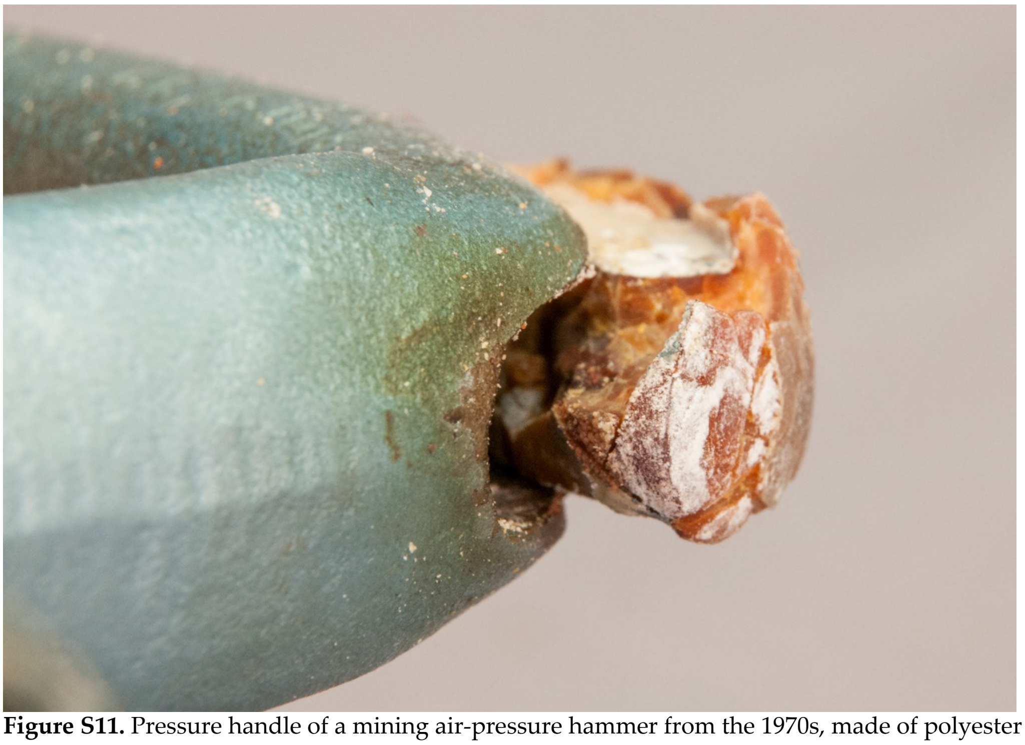

Figure S11. Pressure handle of a mining air-pressure hammer from the 1970s, made of polyester urethane, showing adipic acid as white blooming.

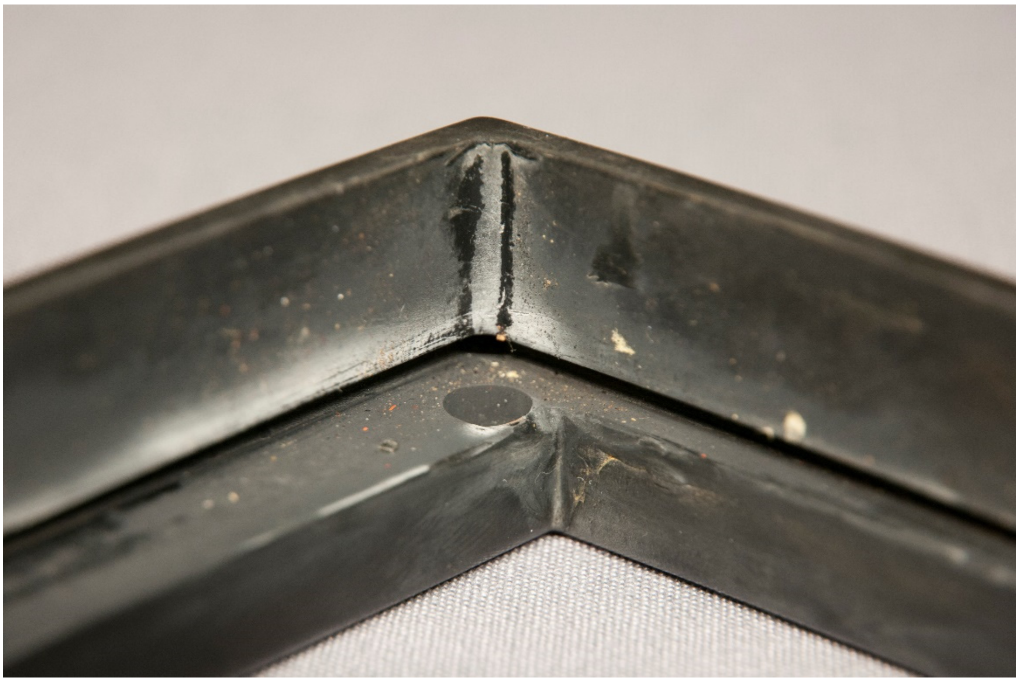

Figure S12. White powdery deposit [blooming (other)] inside a case cover of a gas warning system.

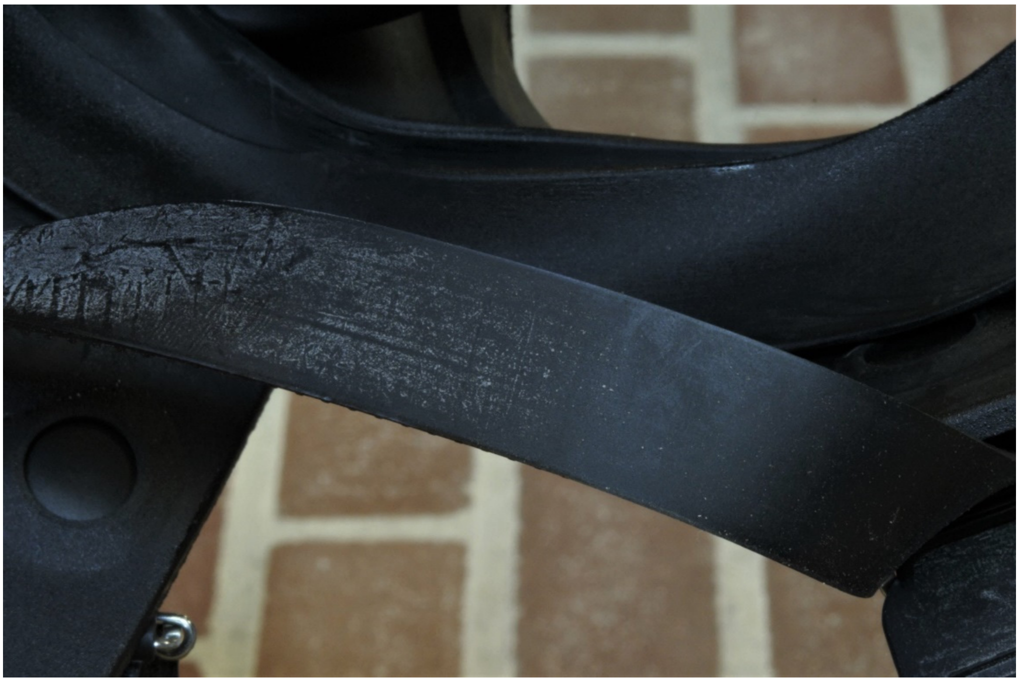

Figure S13. Whitish solid deposit on the surface of a polychloroprene-based headband from a gas mask (picture: F. Pohlmann).

**Break**	Complete crack running through material, with separation of two or more pieces.<br>For films and sheets the term 'tear' is used; fracture.

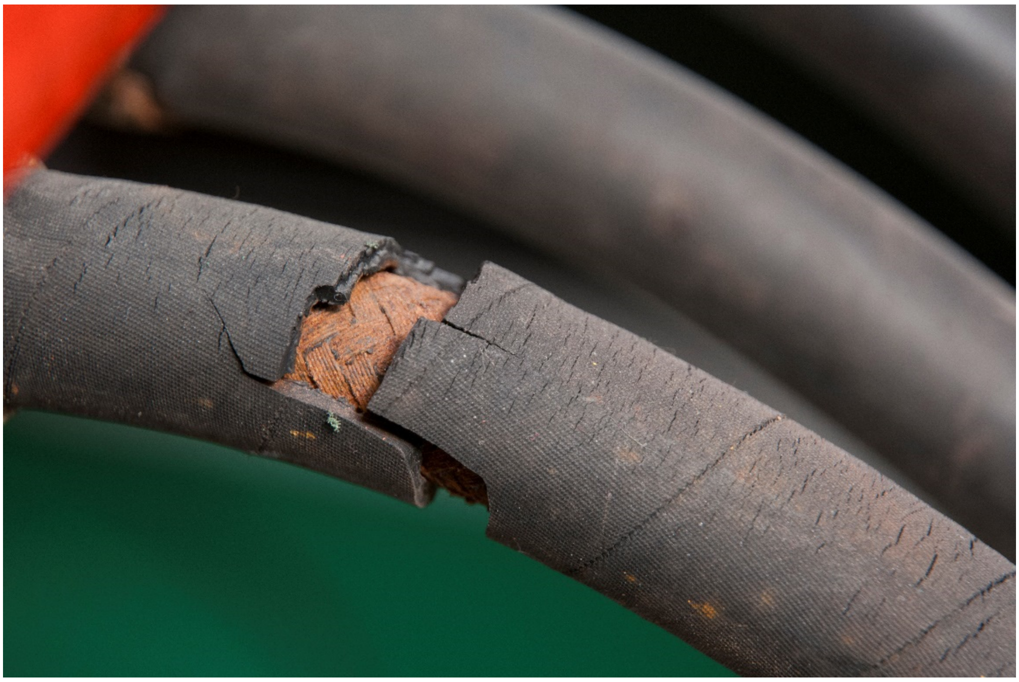

Figure S14. Broken sheath of a elastomeric pneumatic hose, divided in two parts.

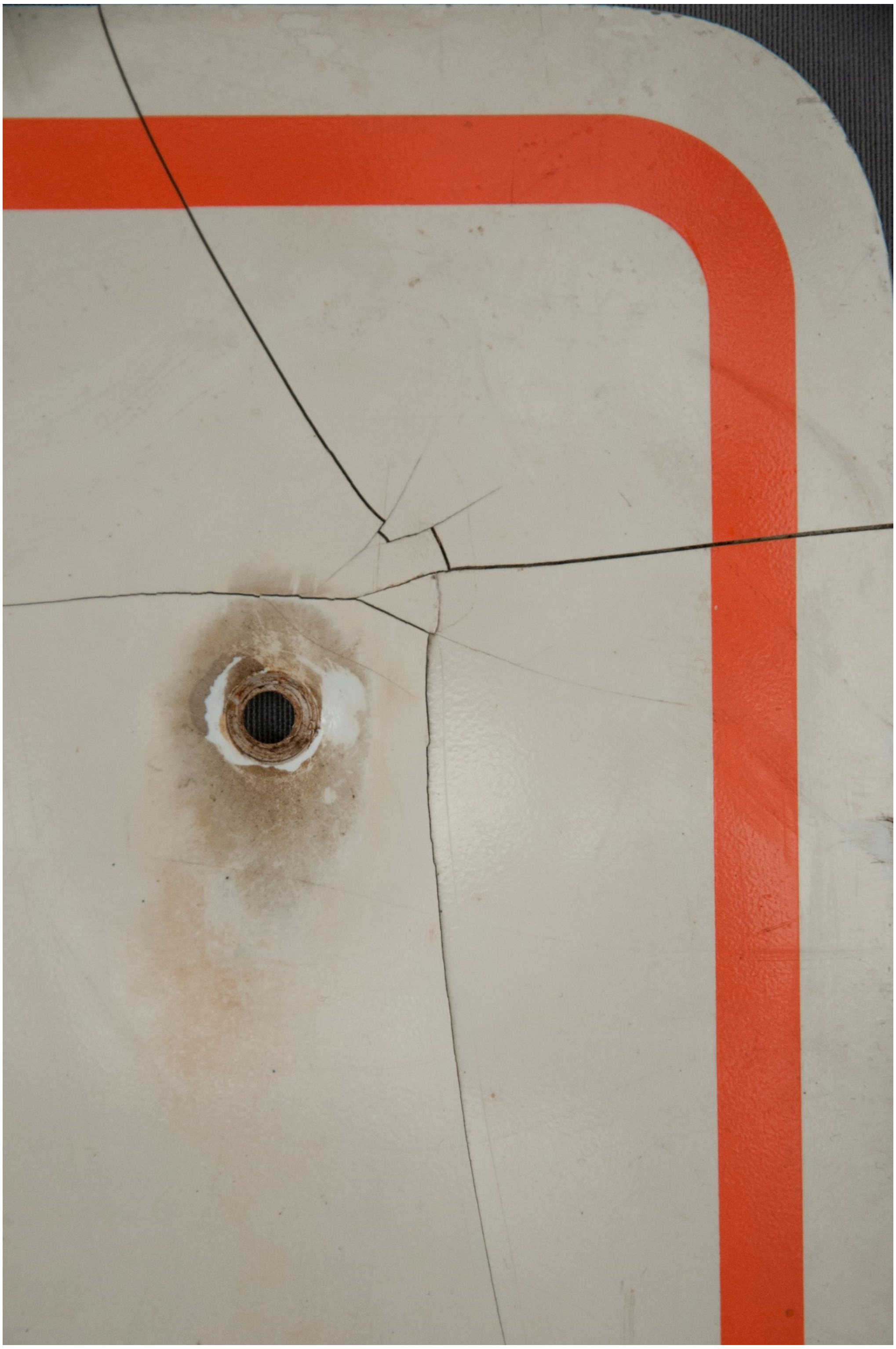

Figure S15. Crack running through the corner area of an information sign, now appearing as a fracture. Made of polymethyl methacrylate-coated unplasticised polyvinyl chloride.

**Brittleness**	Behaviour of materials through which they are likely to crack or break when subjected to pressure.

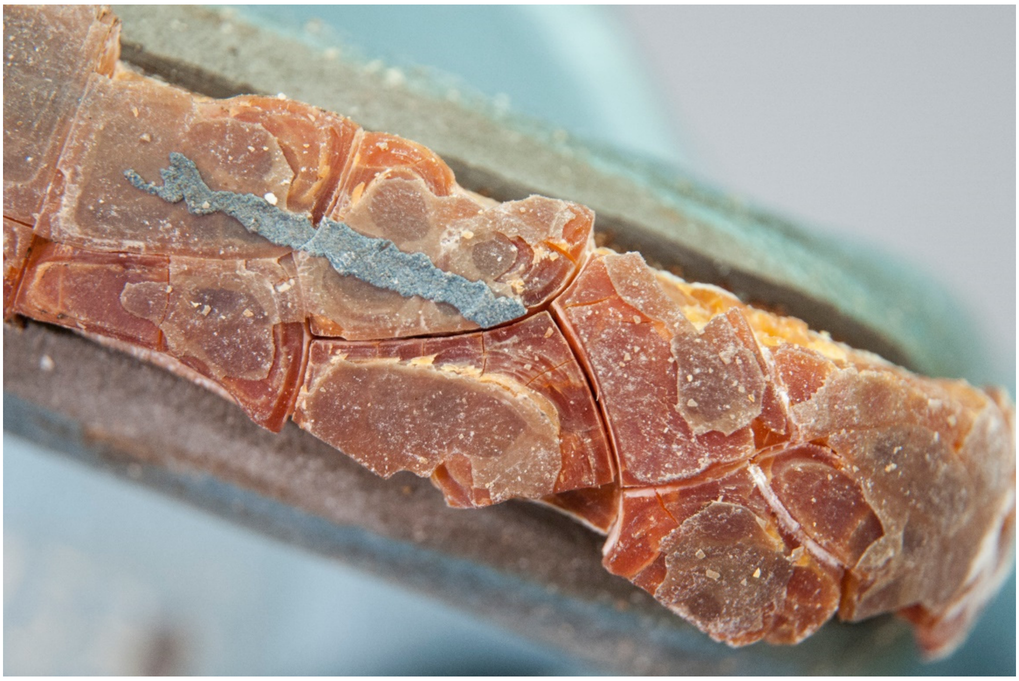

Figure S16. Brittle pressure handle (polyester urethane) of a mining air-pressure hammer showing, among other damages, cracks and flaking.

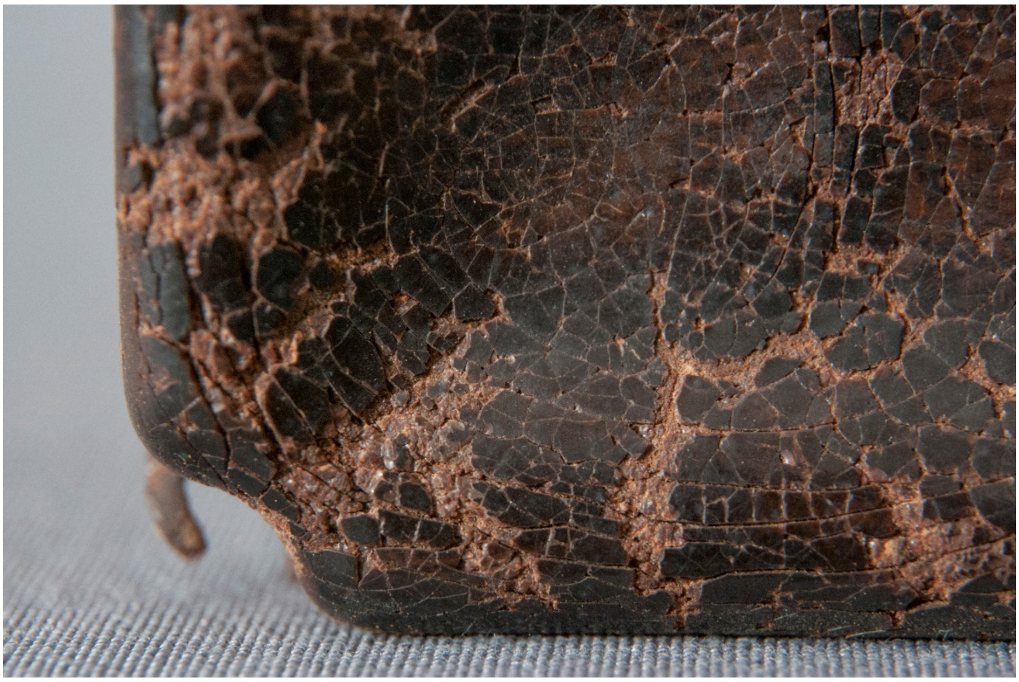

Figure S17. The brittleness of a pillow-shaped rubber bulb of a methanometer led to hardening and craquelure, and eventually flaking.

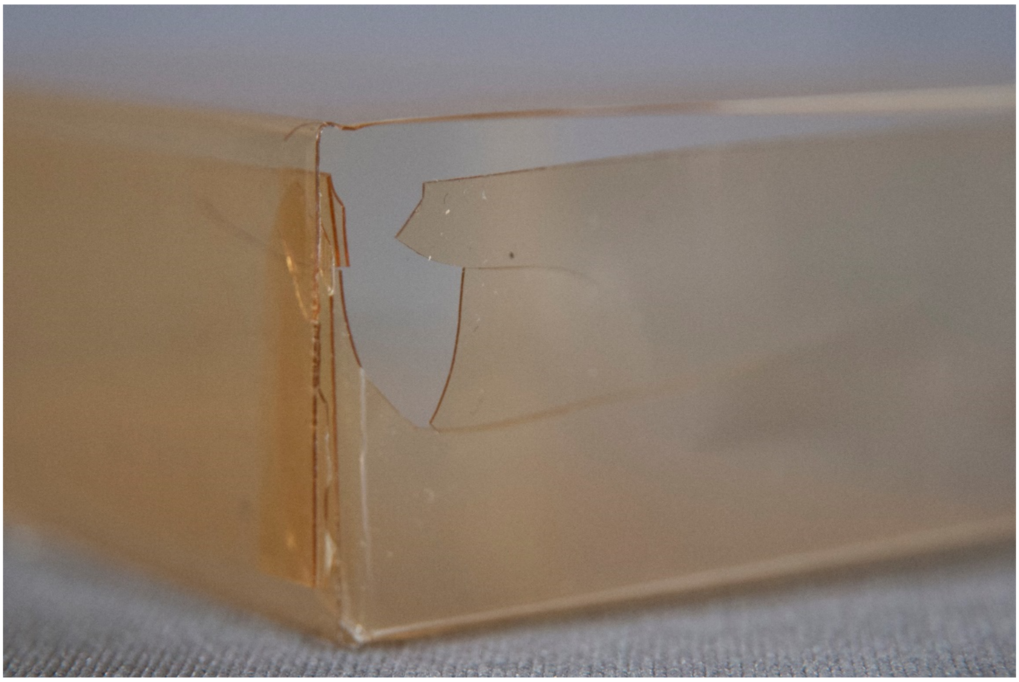

Figure S18. Brittleness led to splintering of the yellowed transparent wrapping of a souvenir (unplasticised polyvinyl chloride).

**Camphor smell**	Aromatic and fresh, menthol-like smell.
**Channeling**	Channel-like detachment between a foil or coating and their undercoat.

**Chip**	Punctual loss of surface material, typically a spalled rim. In material with glass-like behaviour, a conchoidal fracture may appear. (Also: spalling)

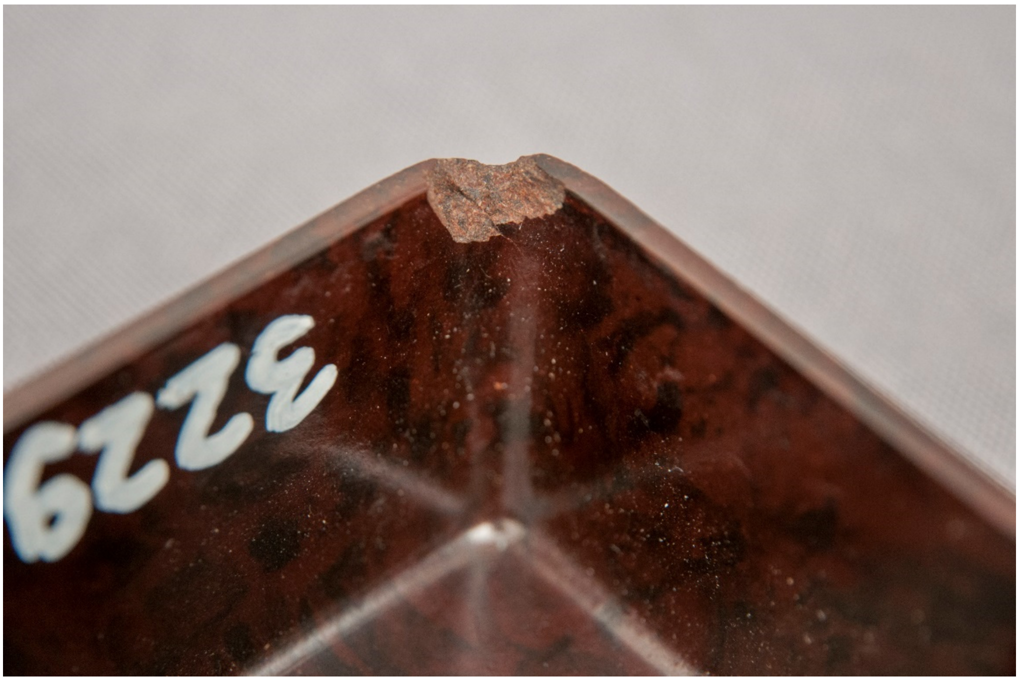

Figure S19. Chipped corner off the bottom part of an ashtray.

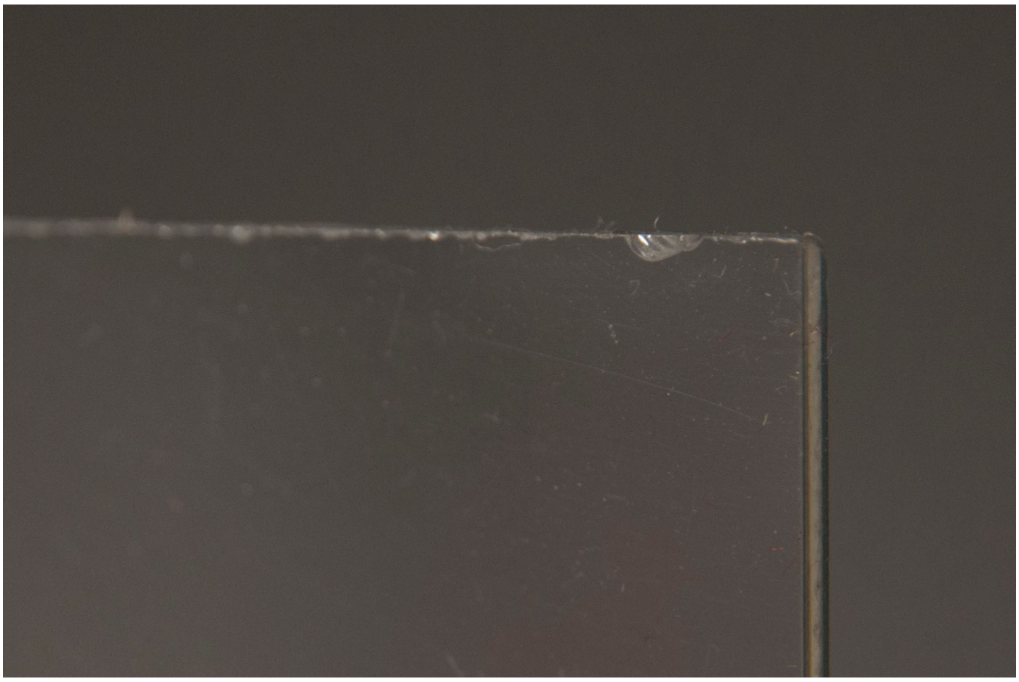

Figure S20. Spalled rim of a transparent display case cover made of unplasticised polyvinyl chloride.

**Corrosion (metal part)**	Products of the degradation of metal elements near plastic parts.

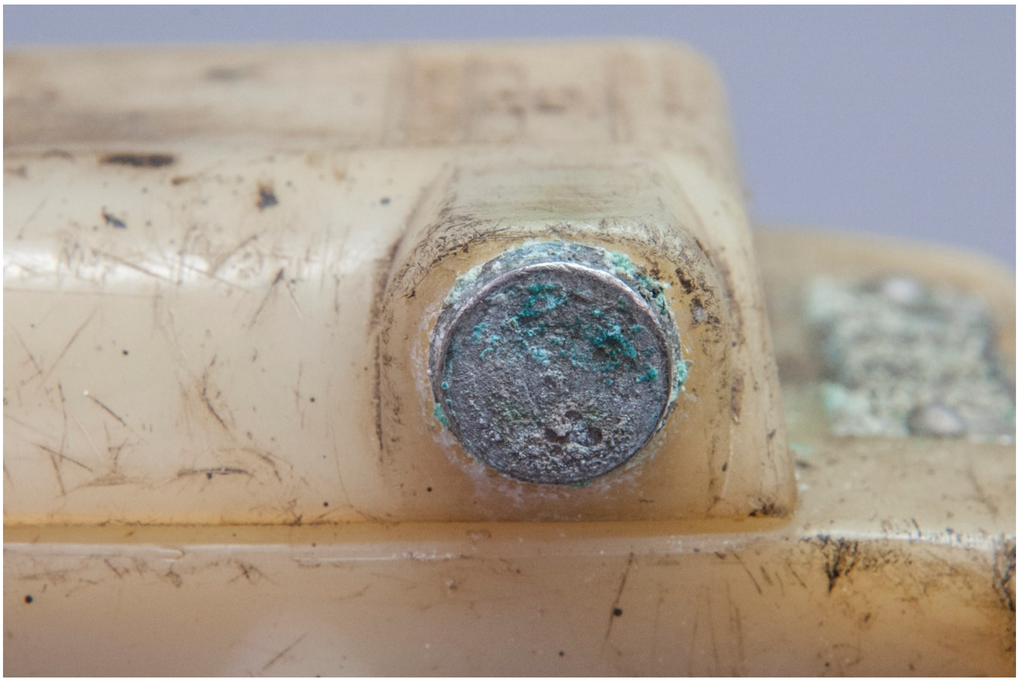

Figure S21. Corroded metal part (copper alloy) from the top of a miner's battery lamp, surrounded by polyamide plastic parts.

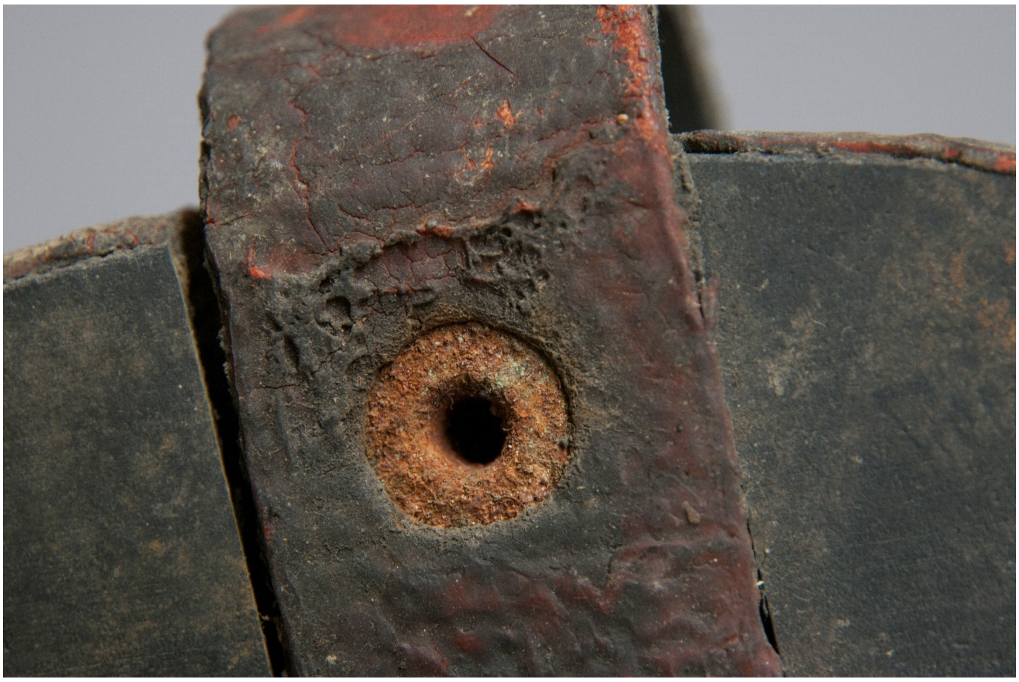

Figure S22. Corroded steel rivet surrounded by polyisoprene-based rubber in bathing slippers.

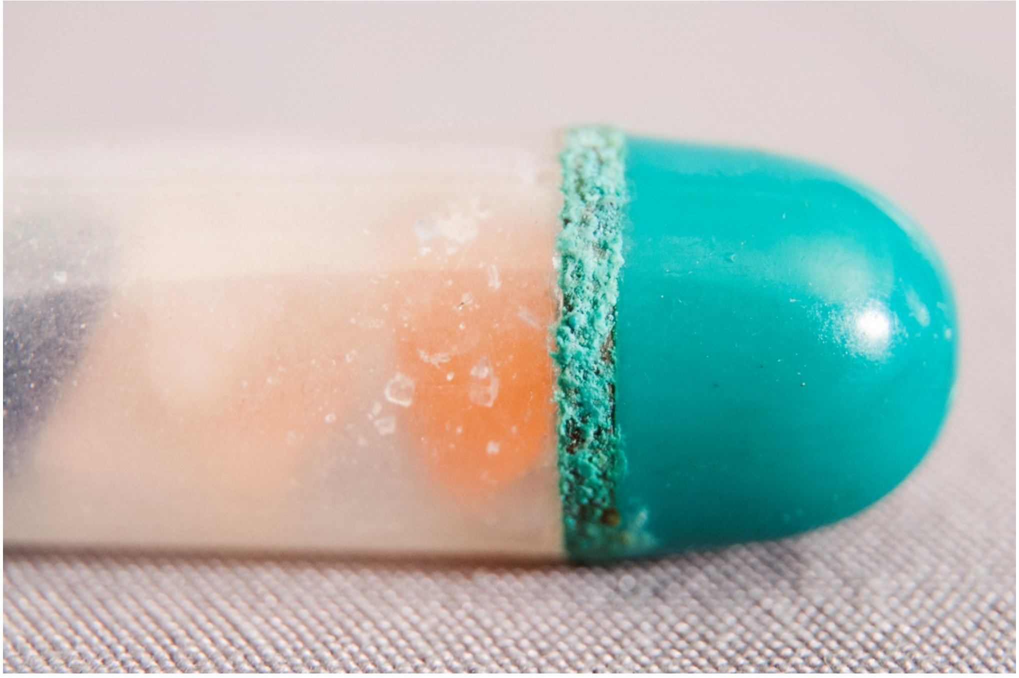

Figure S23. Green corrosion products of a copper alloy in a ballpoint pen made of transparent and teal polystyrene.

**Crack**	Small fissure or opening which does not cause separation of an object part (cf. break).

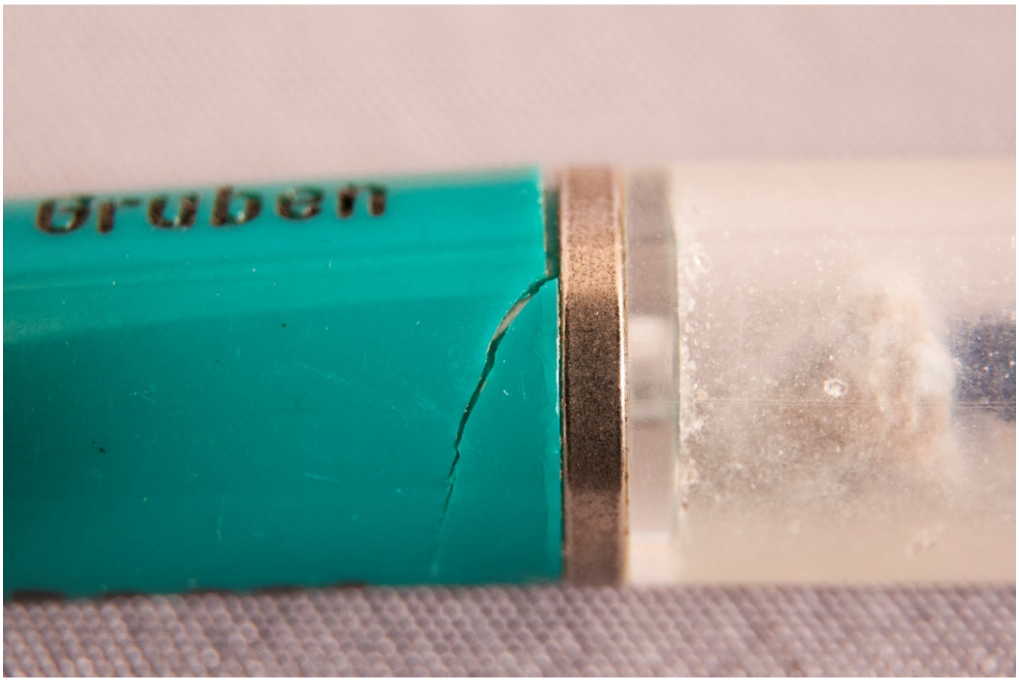

Figure S24. Cracked polystyrene ballpoint pen case.

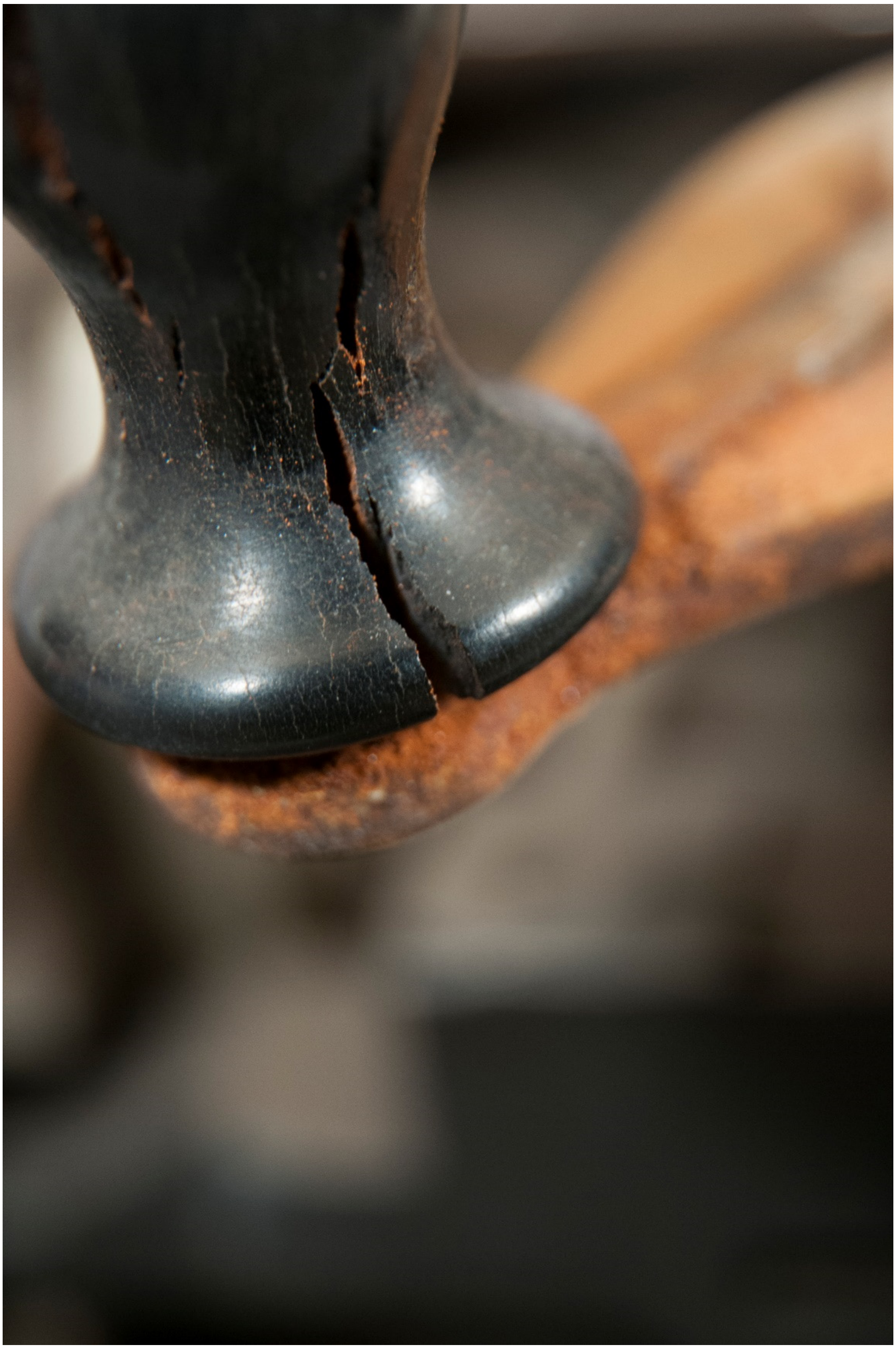

Figure S25. Gaping crack in a crank handle of a calculator (early 20th century)

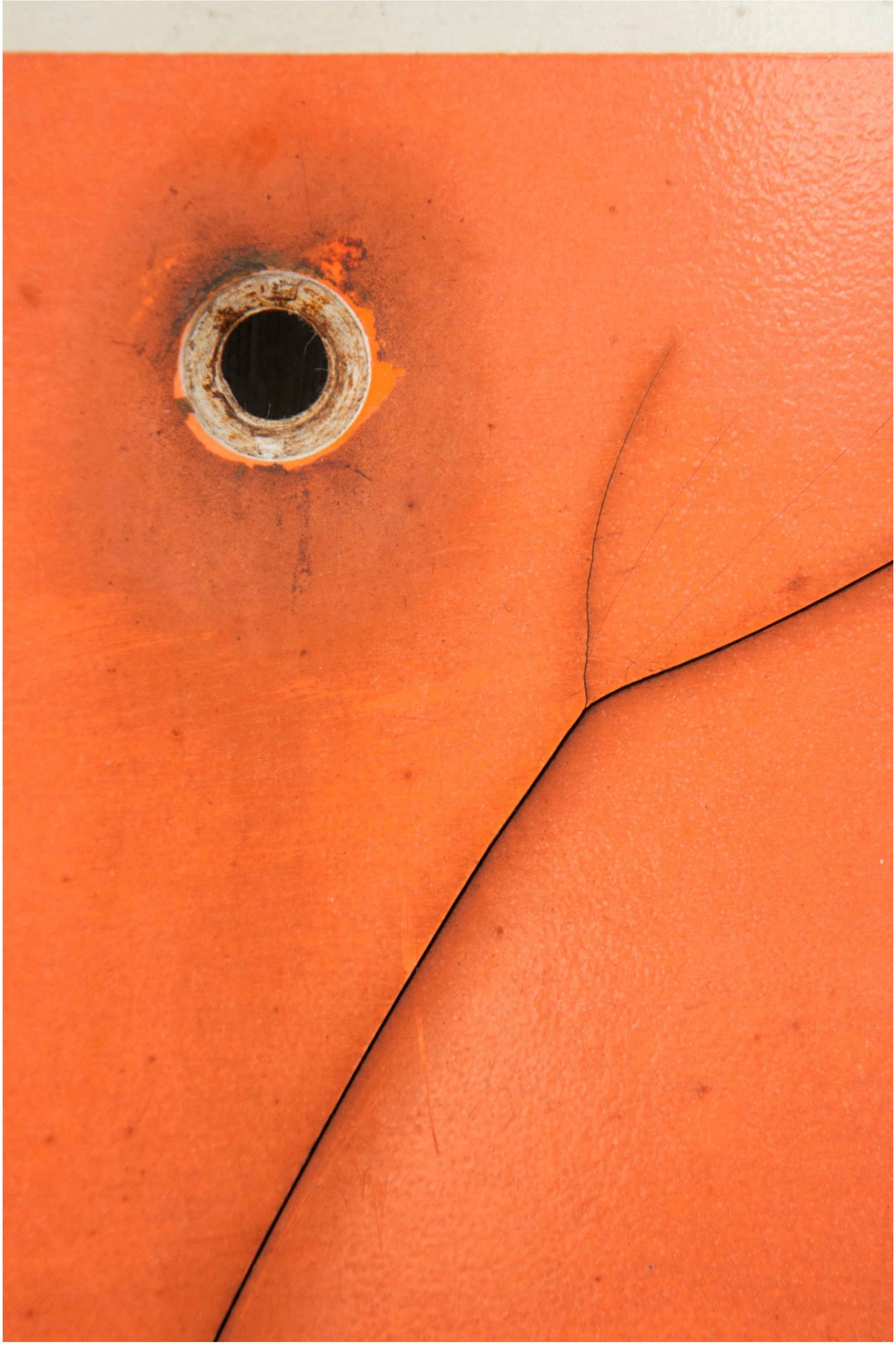

Figure S26. Fissure in the plate of an information sign (polymethyl methacrylate-coated unplasticised polyvinyl chloride).

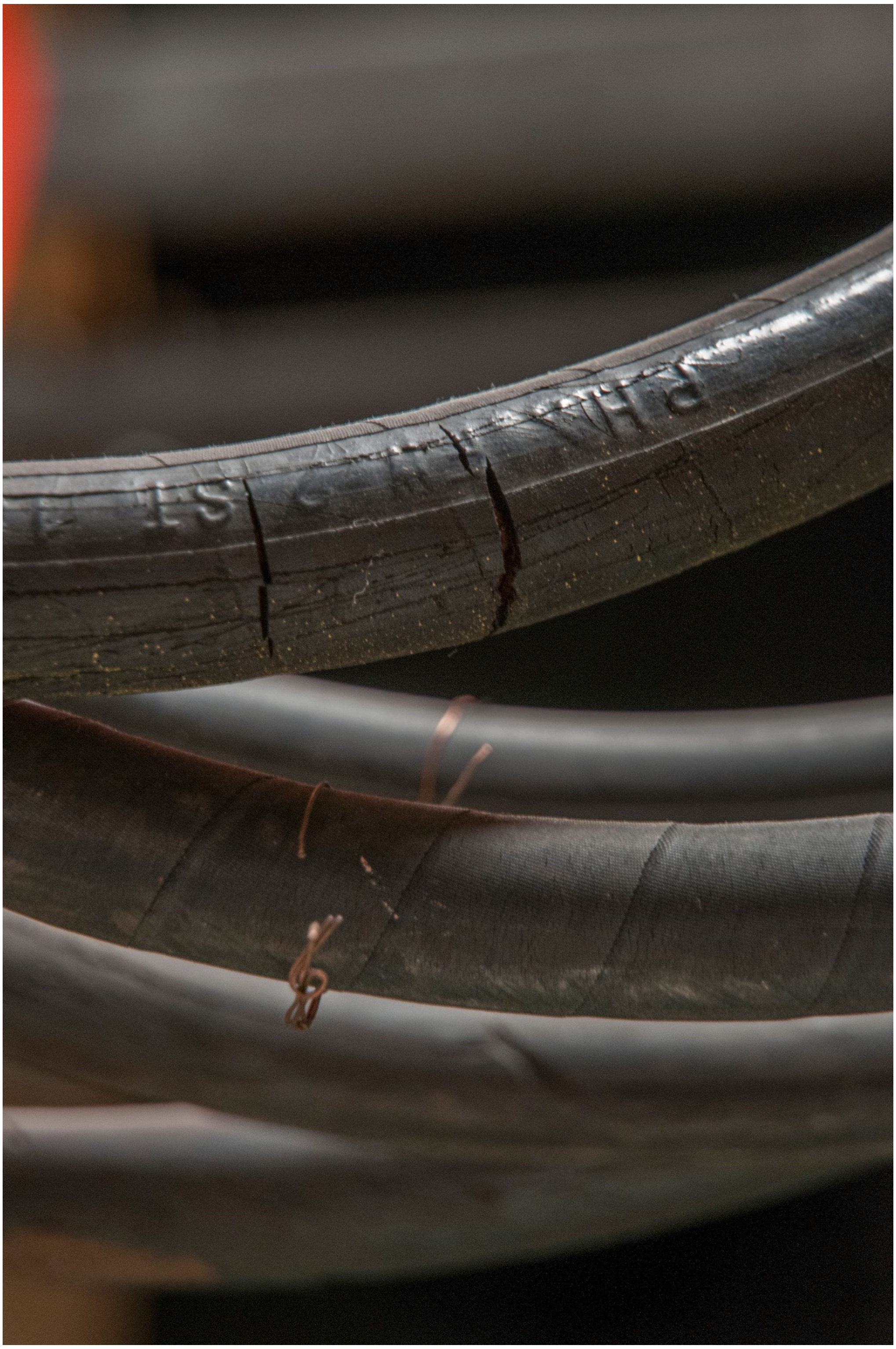

Figure S27. Transversal cracks in the sheath of an elastomeric pneumatic hose.

**Craquelure**	Superficial network of cracks or fissures affecting the outmost layers of a material, such as is typically found in varnishes.

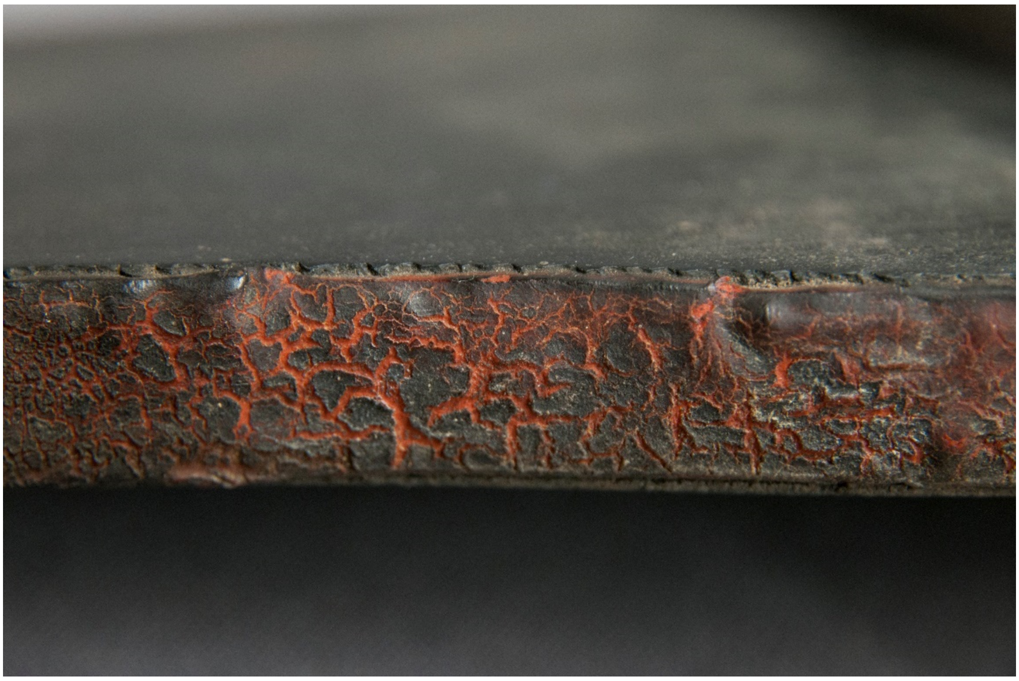

Figure S28. Craquelure in the form of interconnecting cracks in a rubber strip (polyisoprene-based).

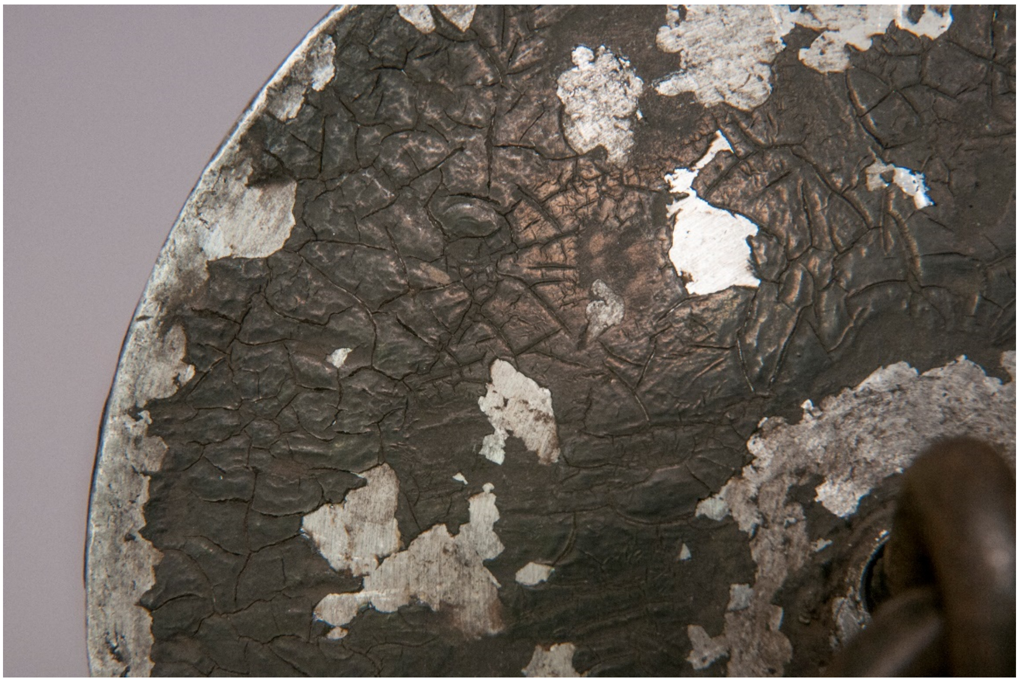

Figure S29. Craquelure in the paintwork on metal parts of a miner's lamp, leading to flaking.

**Crazing**	Crazing may refer to a) internal micro-crack region, macroscopically visible as stress whitening with possible loss of transparency, or b) network of fine cracks starting in the surface or within a material, which spreads throughout it. Case b) typically affects cellulose nitrate and may be accompanied by concomitant whitening (loss of transparency).

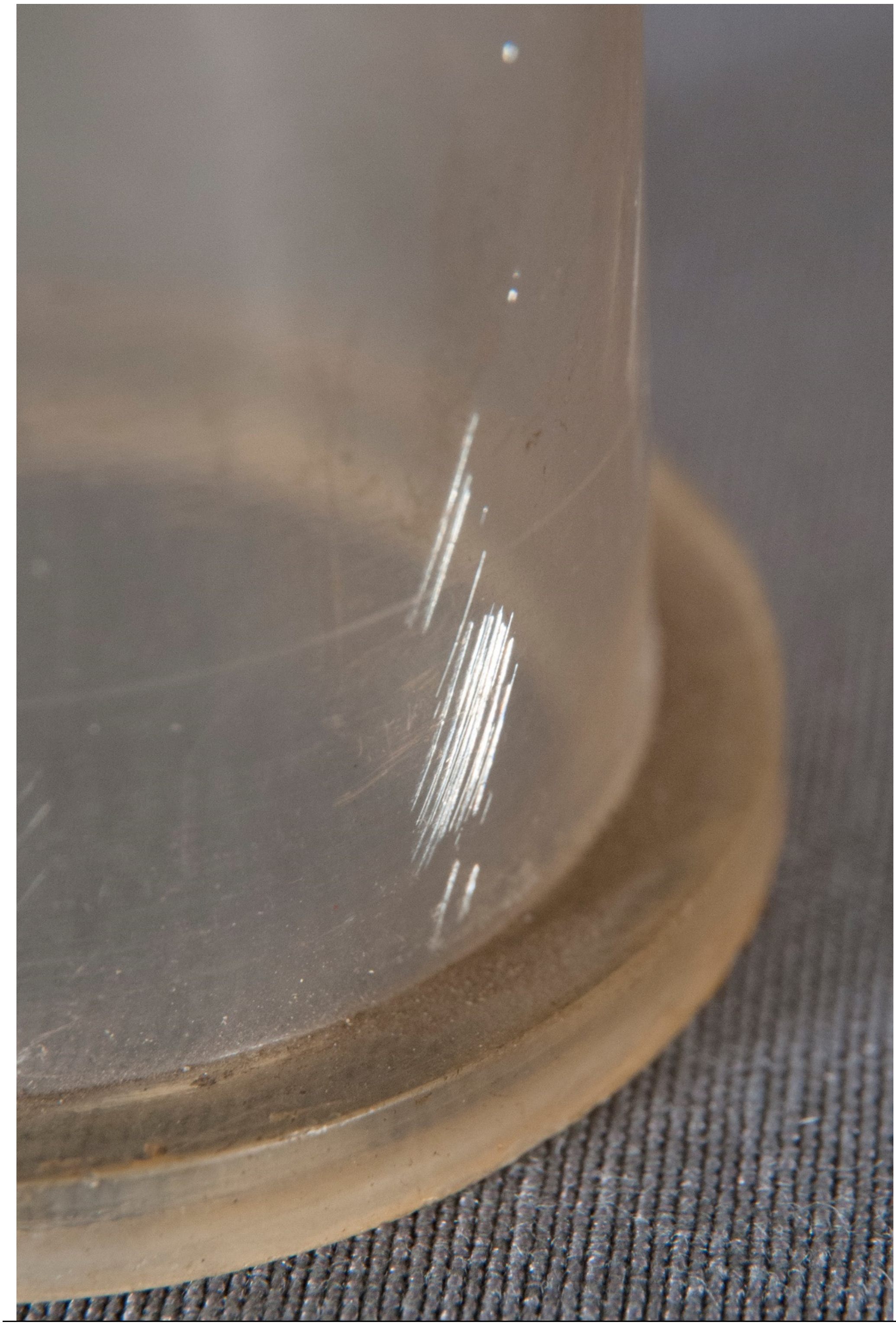

Figure S30. Crazes in the transparent protection cover (polystyrene) of the light bulb of a miner's lamp, visible in oblique light [type a) in the description of <crazing>].

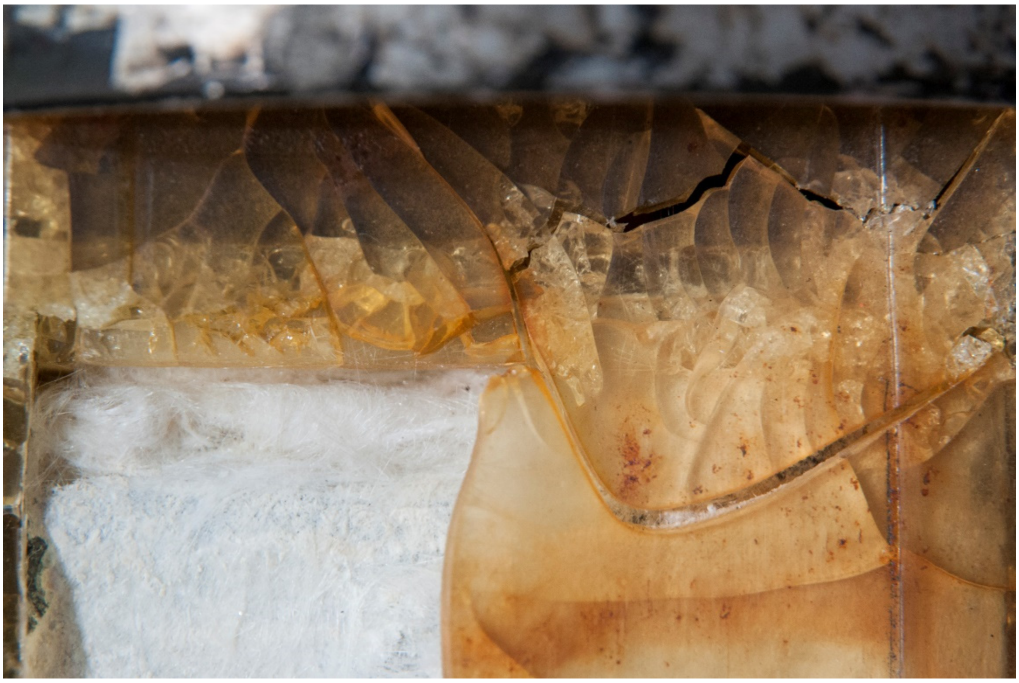

Figure S31. Typical crazing in the yellowed battery case from a miner's lamp (cellulose nitrate); with concomitant transparency loss [type b) in the description of <crazing>].

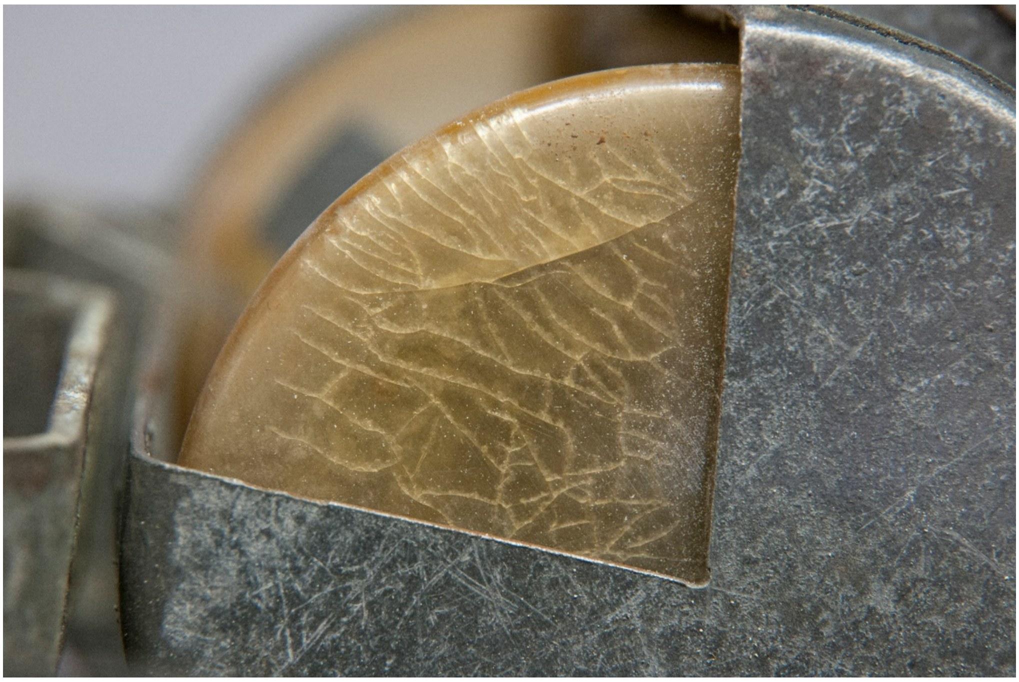

Figure S32. Crazing in a battery case from a miner's lamp made of cellulose nitrate [type b) in the description of <crazing>].

**Crumbling**	Detachment of material due to loss of coherence, characterised by extensive friability that usually starts in the surface and advances into the depth of the material. It can appear e.g. in the form of cube break, or, in the case of foams, lead to powder in its final stages.

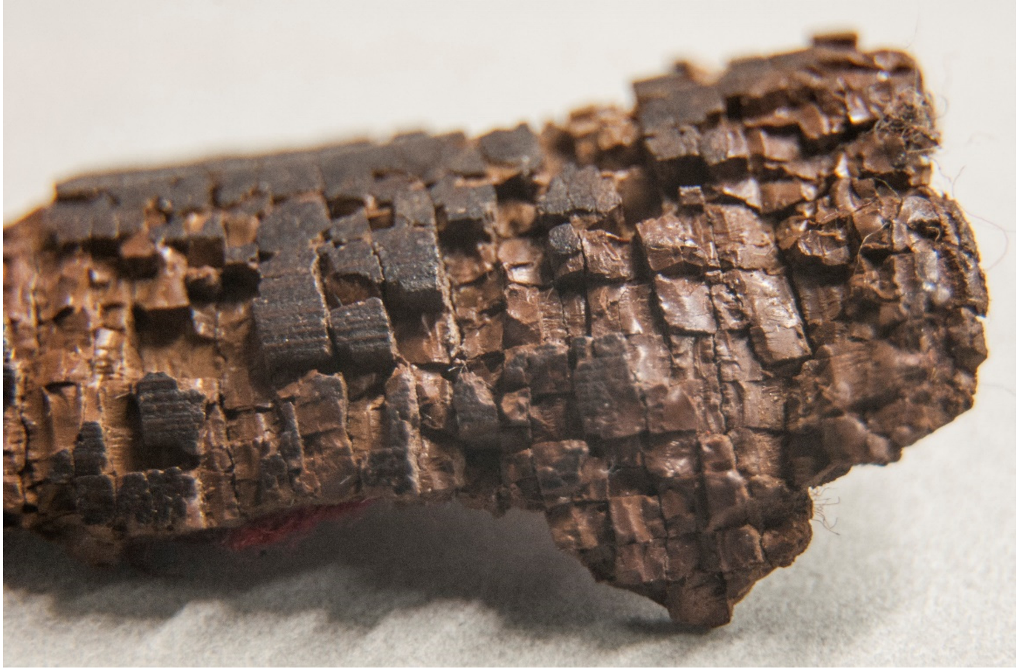

Figure S33. Crumbling as cube break in an electric cable sleeve (polychloroprene) from a battery locomotive.

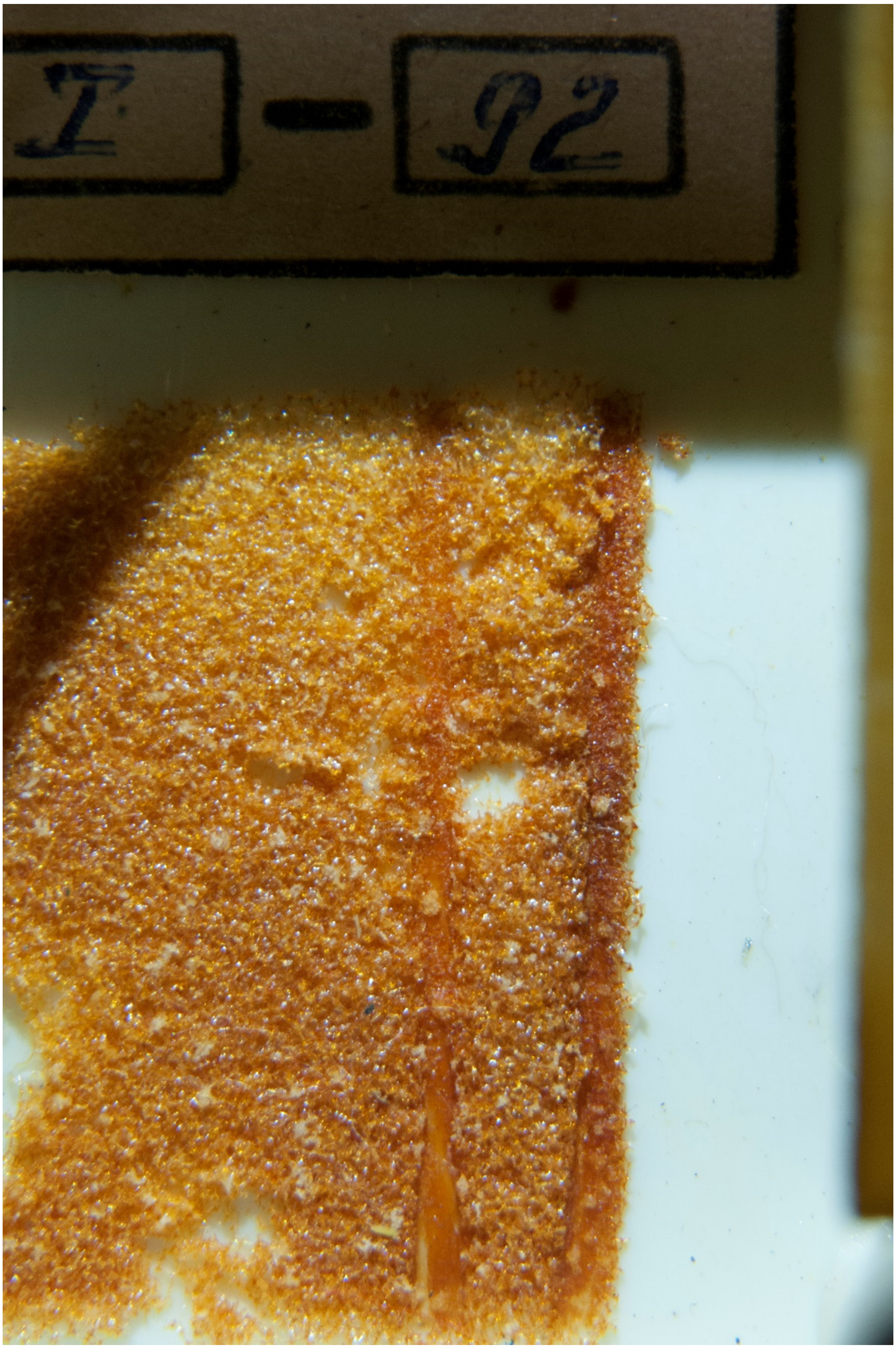

Figure S34. Crumbling polyurethane foam in the battery compartment of a radiation dosimeter.

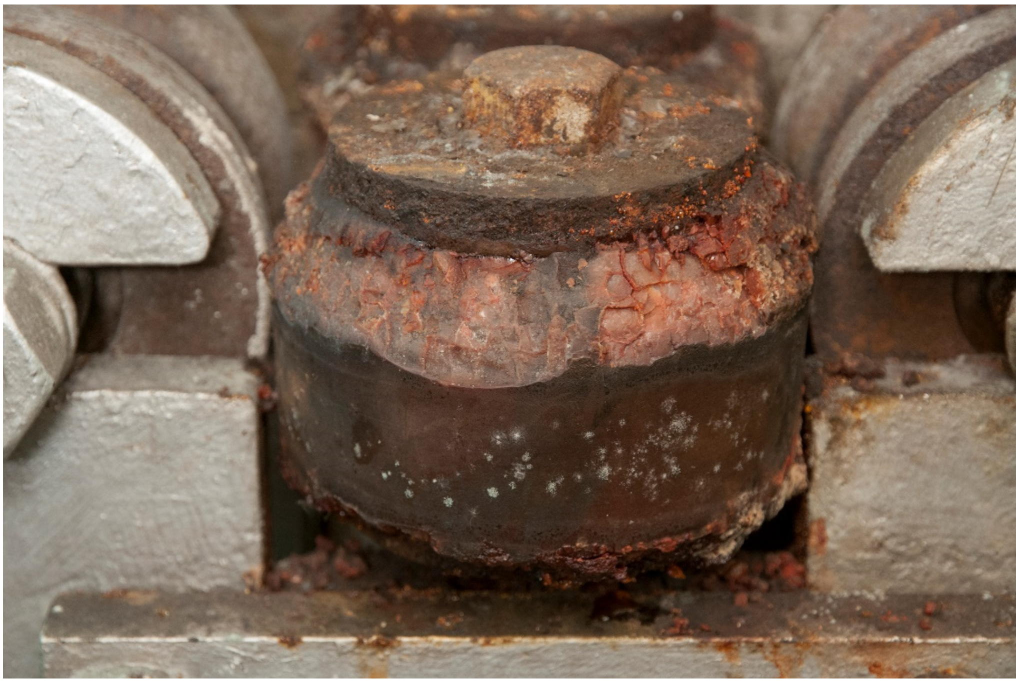

Figure S35. Crumbling friction drive (wheel) [polyester urethane] of a monorail system.

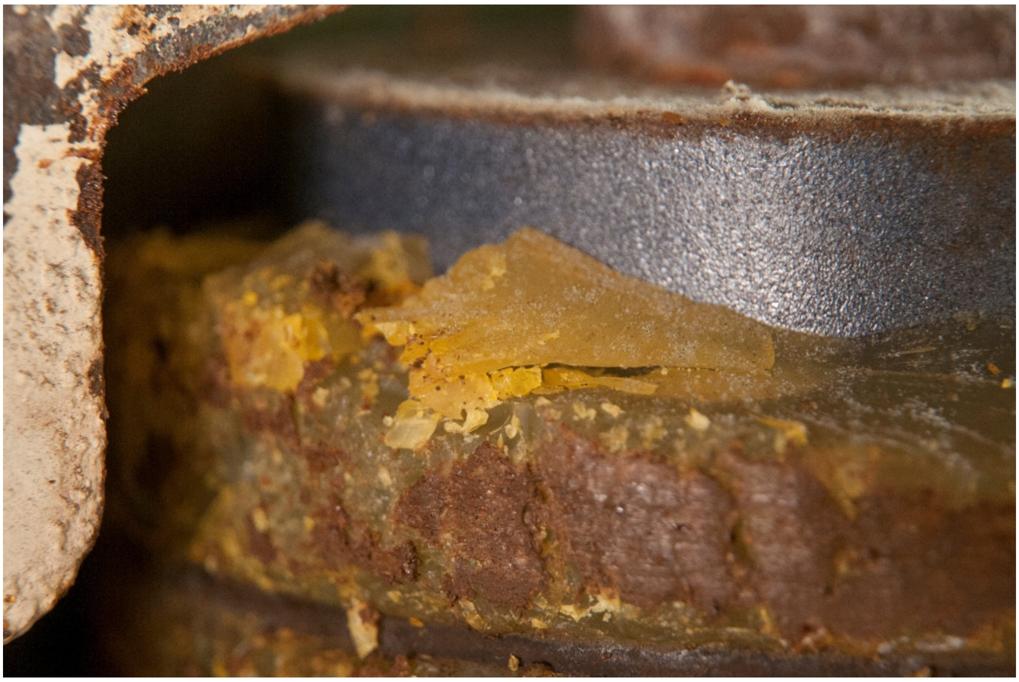

Figure S36. Crumbling with loss of and large fragments in the friction drive (wheel) of a monorail system (polyester urethane).

**Dent**	Three-dimensional form change appearing as a hollow mark, buckle or bump; indentation.

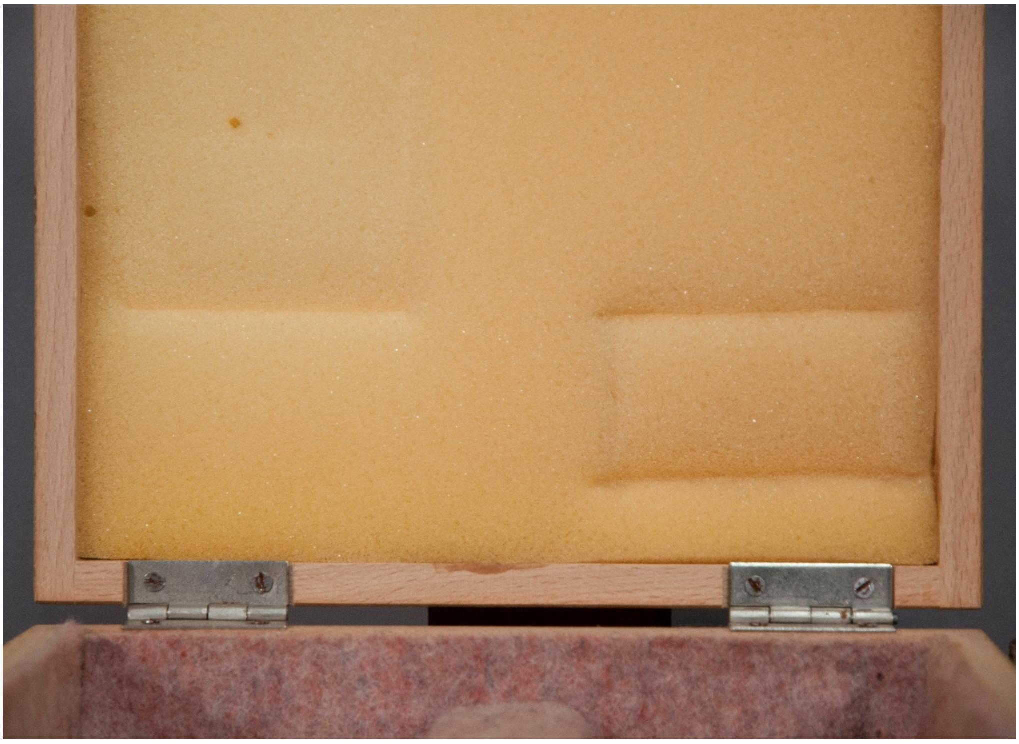

Figure S37. Impression marks of a gadget, visible in the polyurethane foam on the box lid.

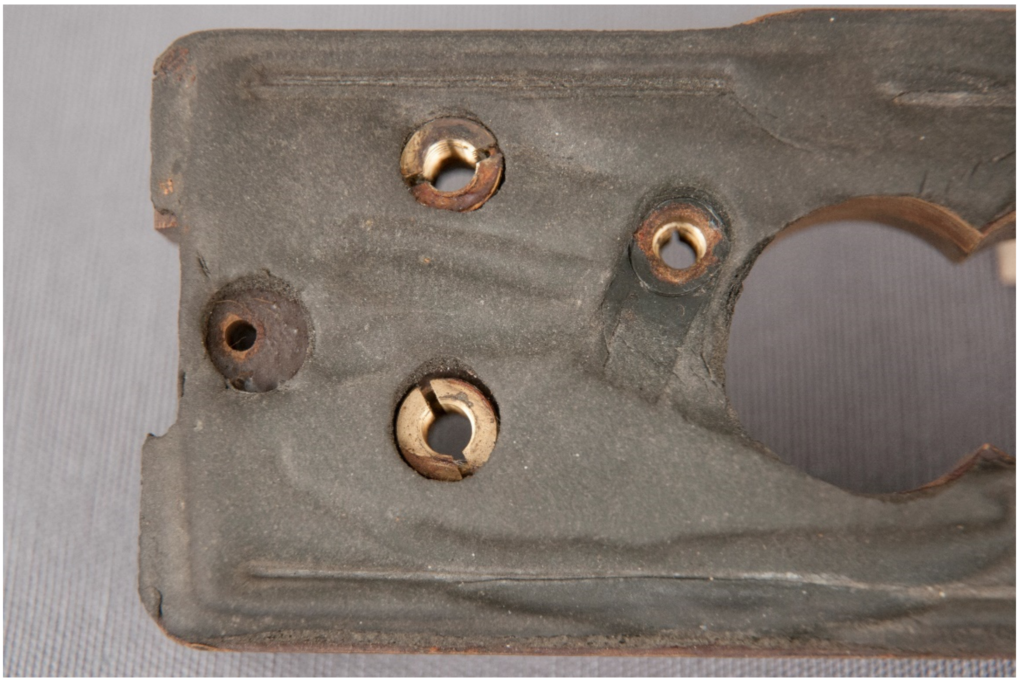

Figure S38. Dented foam pad in the top part of a miner's battery headlamp.

**Dirt**	Solid soiling other than dust or stains, regardless of whether it can be found localised or extended in a wide area.

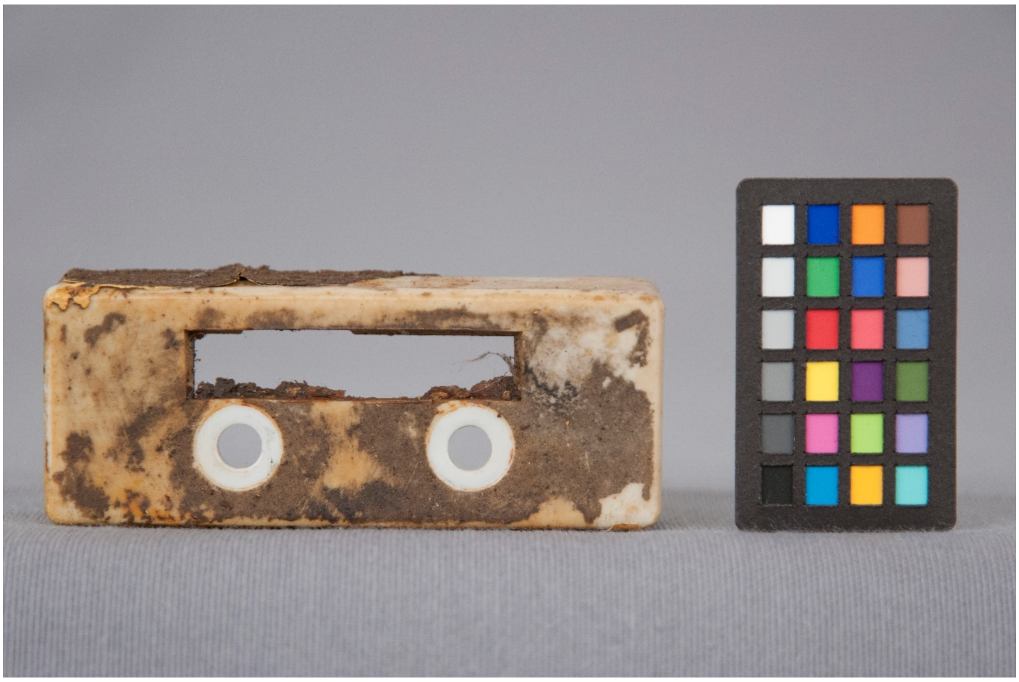

Figure S39. Soiled cover of a hydraulic oil filter display made of polyamide.

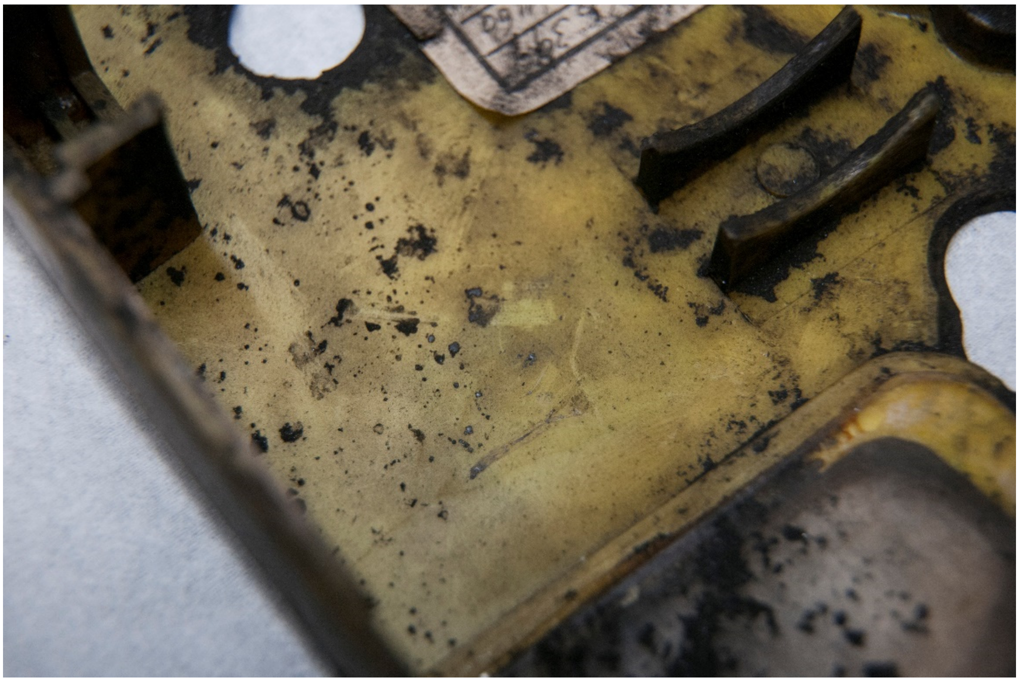

Figure S40. Dirt in the form of coal dust inside a methanometer.

**Discolouration**	Any change in colour of the original material other than fading or yellowing.

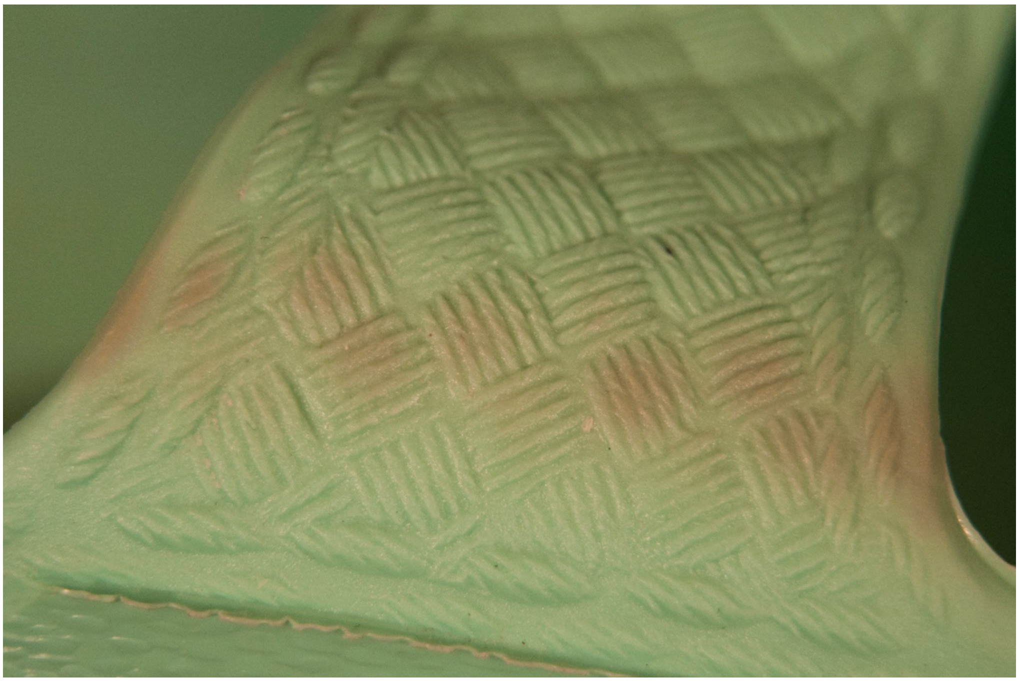

Figure S41. Changed colour in green bathing slippers (plasticised polyvinyl chloride).

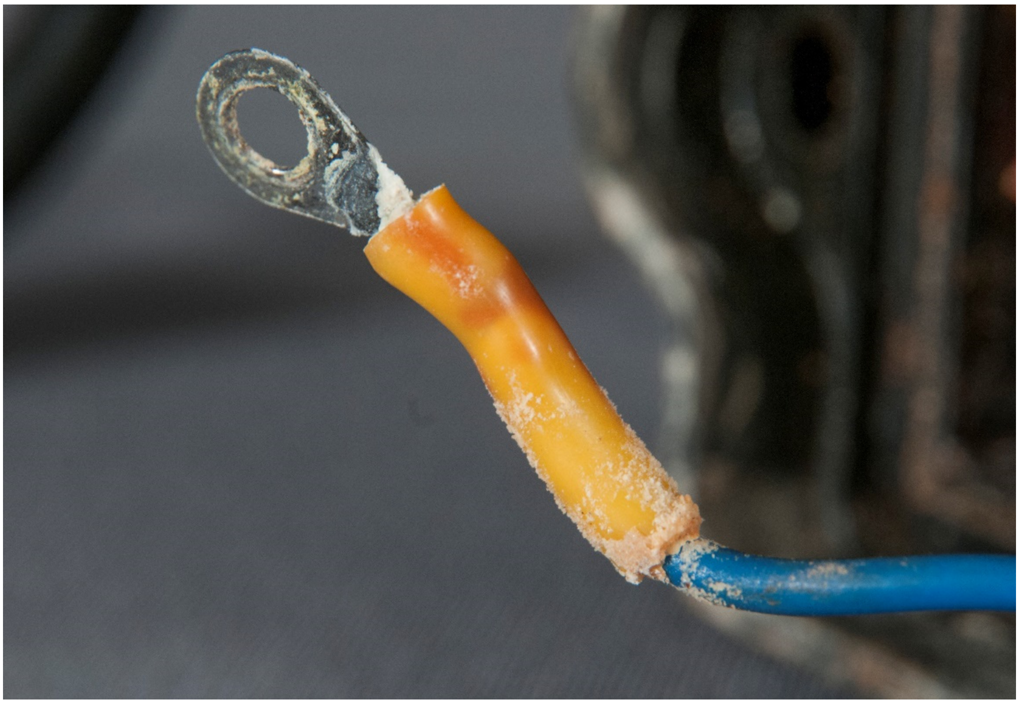

Figure S42. Red-brown colour appearing in a yellow cable sleeve (plasticised polyvinyl chloride).

**Dried fluid**	Solid material left behind as a residue after the drying of a liquid deposit. It usually appears in the form of a coloured, highly glossy semitransparent substance that sometimes shows some visual feature hinting to the previous liquid state of the substance (e.g. drop form).

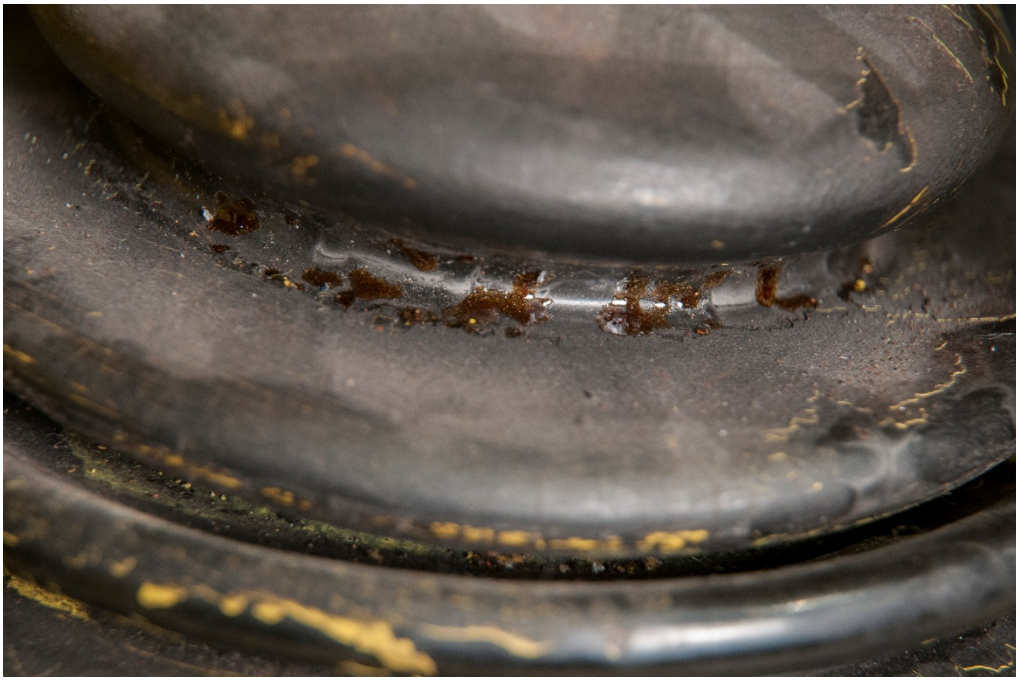

Figure S43. Dried fluid deposit on the surface of a gearshift bag (polyvinyl chloride).

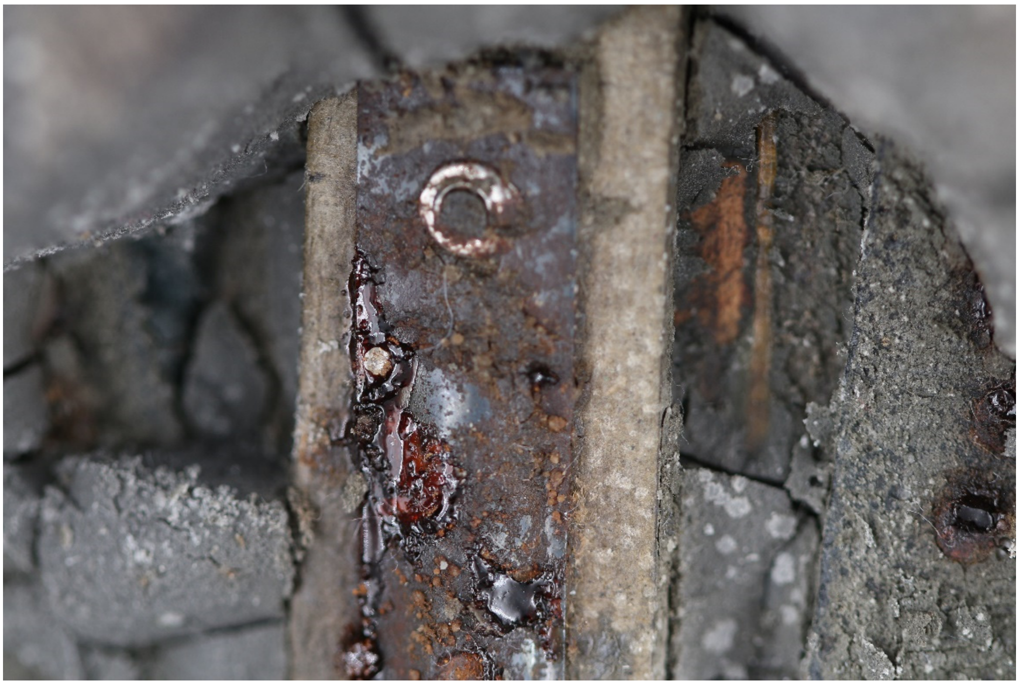

Figure S44. Solidified liquid deposit on a polyester urethane shoe sole (picture: G. Gasperuzzo).

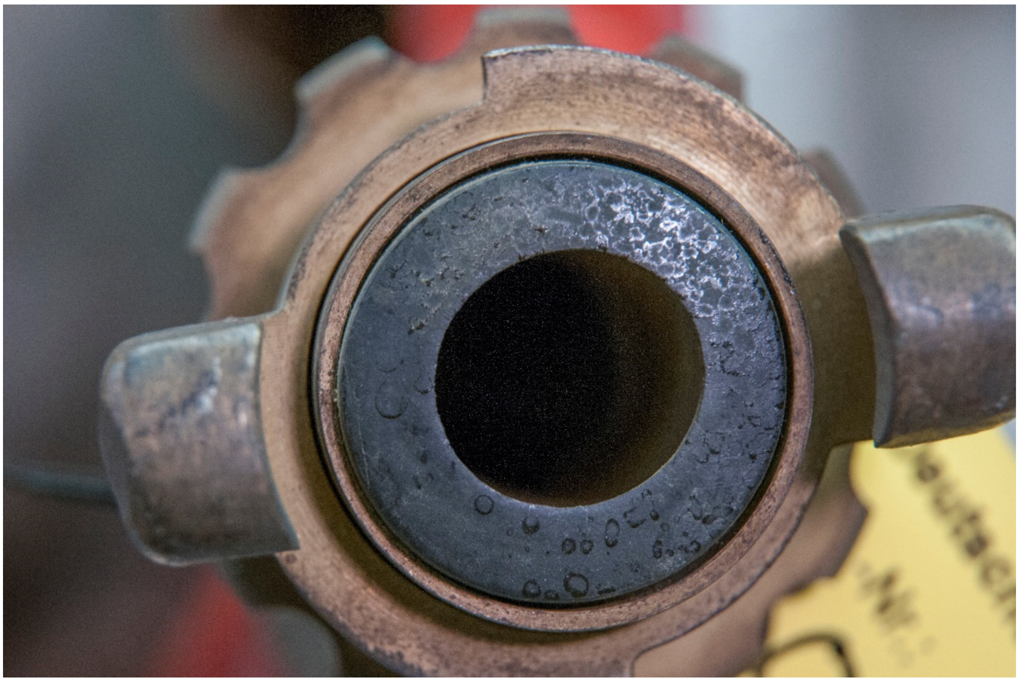

Figure S45. Drop-shaped solid residues on a hose connection.

**Dust**	Fine air-borne particles that deposit more or less extensively, and typically uniformly, in surfaces. Dust can be found either simply lying or adhered to these in combination with other substances (e.g. dirt deposition such as fat).

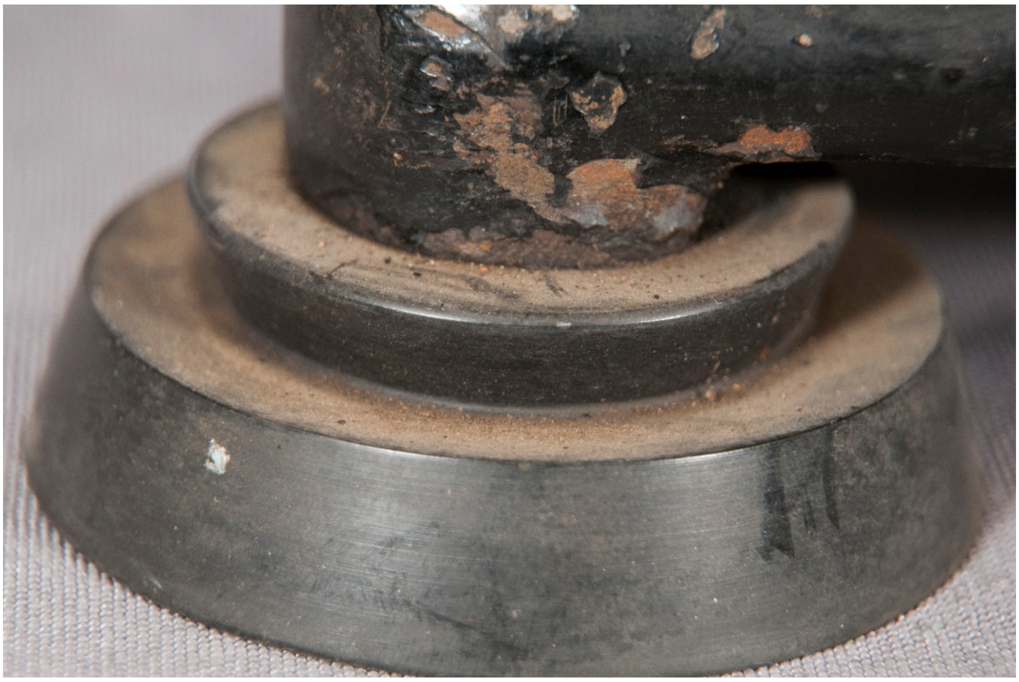

Figure S46. Dust on horizontal surfaces of the stand of a calculator.

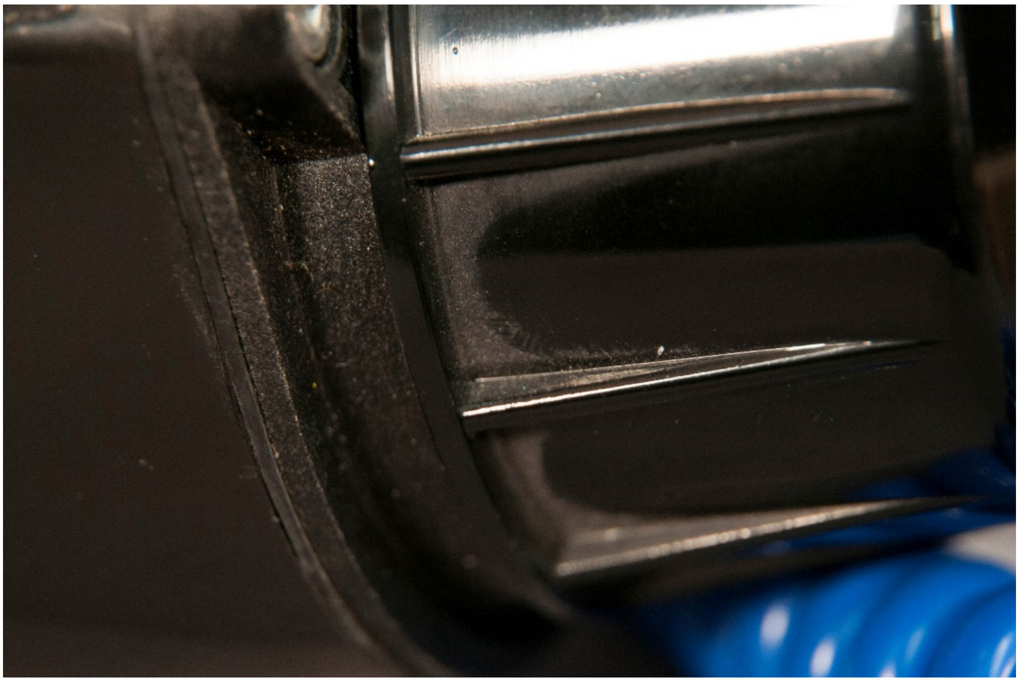

Figure S47. Dusty corners on a telephone handset.

**Fading**	Reduced colour brightness or intensity.

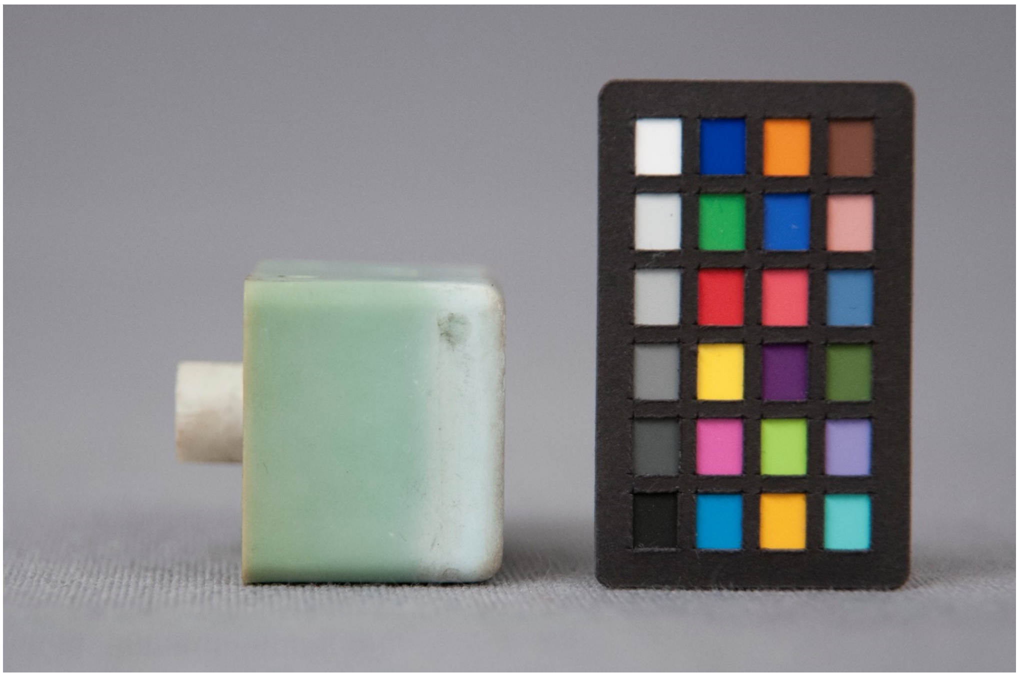

Figure S48. Faded area of a green control button (polyoxymethylene) from a mine control station.

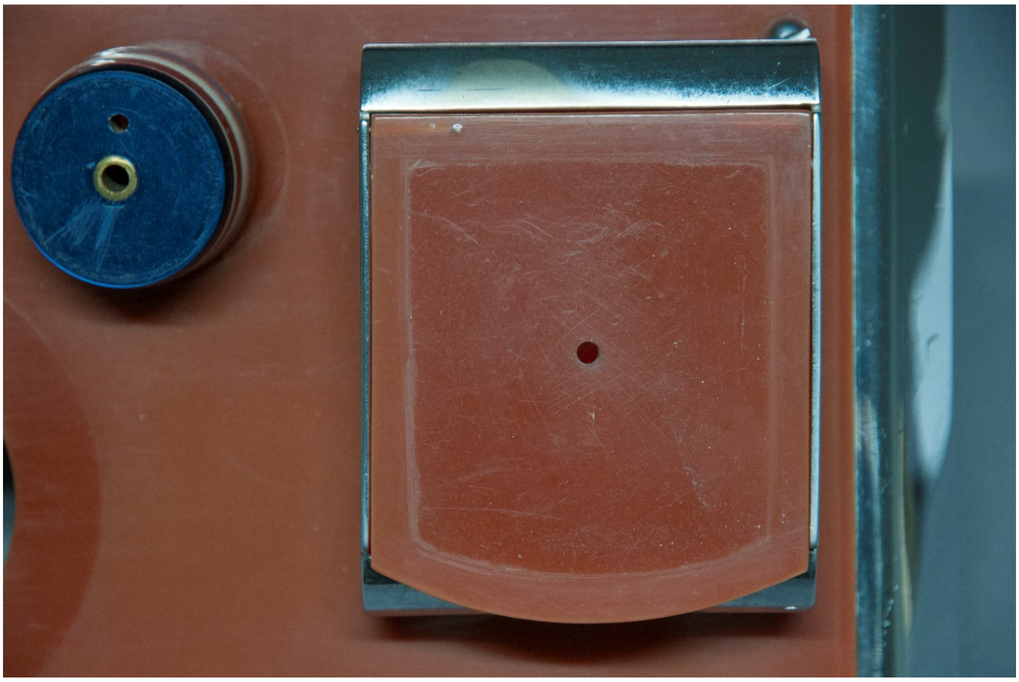

Figure S49. Fading in uncovered areas (compare with Figure 50) of dark brown sheets (unplasticised polyvinyl chloride) of an ashtray set.

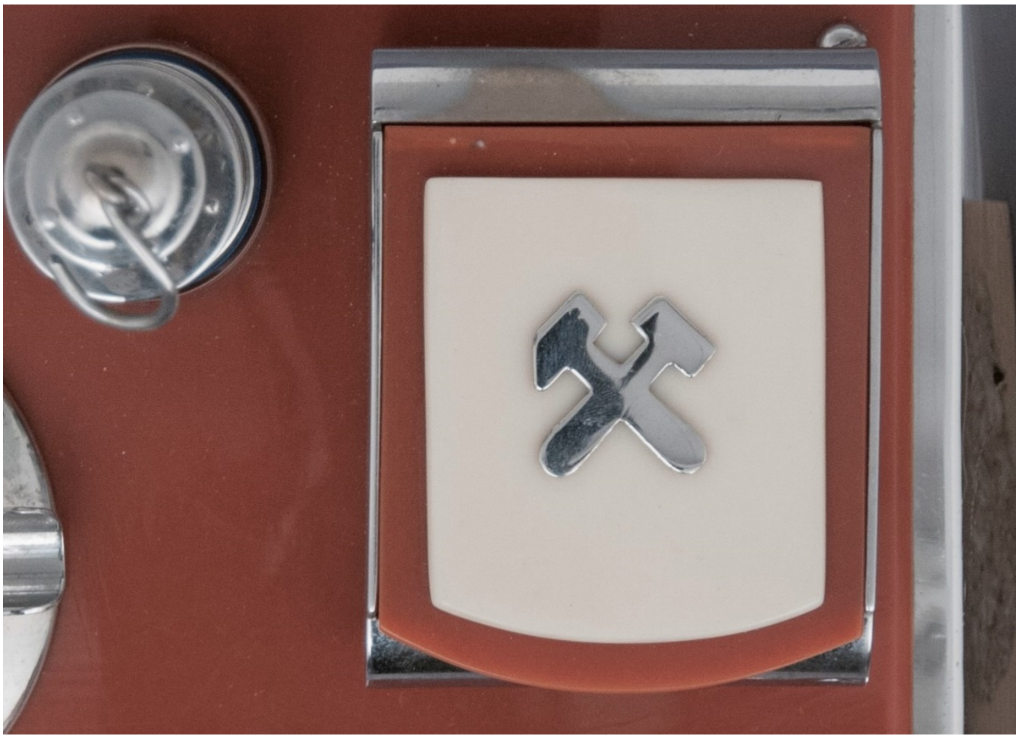

Figure S50. Complete ashtray set, for comparison with Figure 49.

**Flaking**	Usually applied to the detachment of material in the form of small flat platelets.

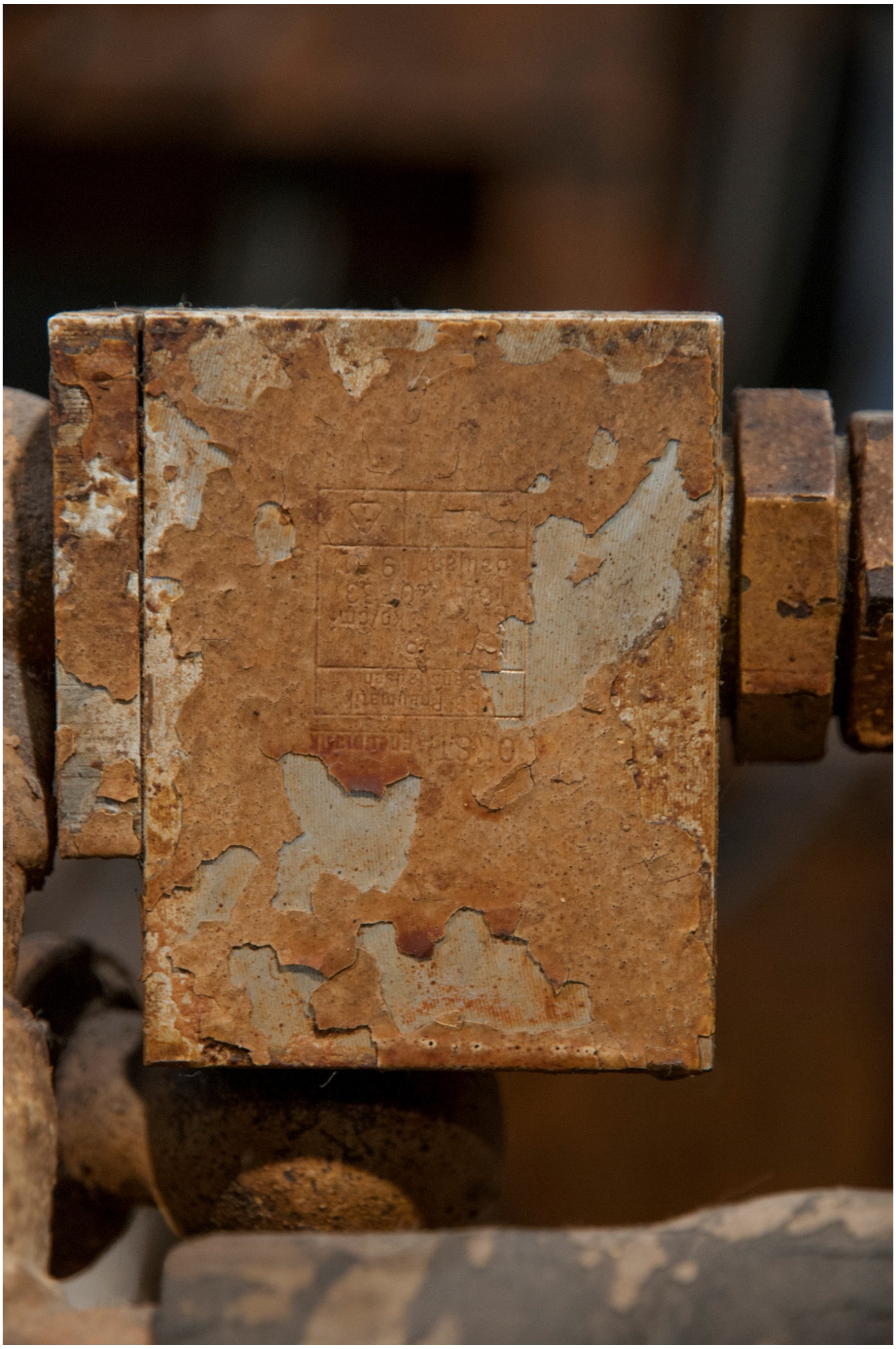

Figure S51. Detaching of a paintwork in the form of flakes.

**Fold**	A bend, crease or kink of an object on itself, causing after a certain time stress in the material, eventually leading to partially irreversible damage.

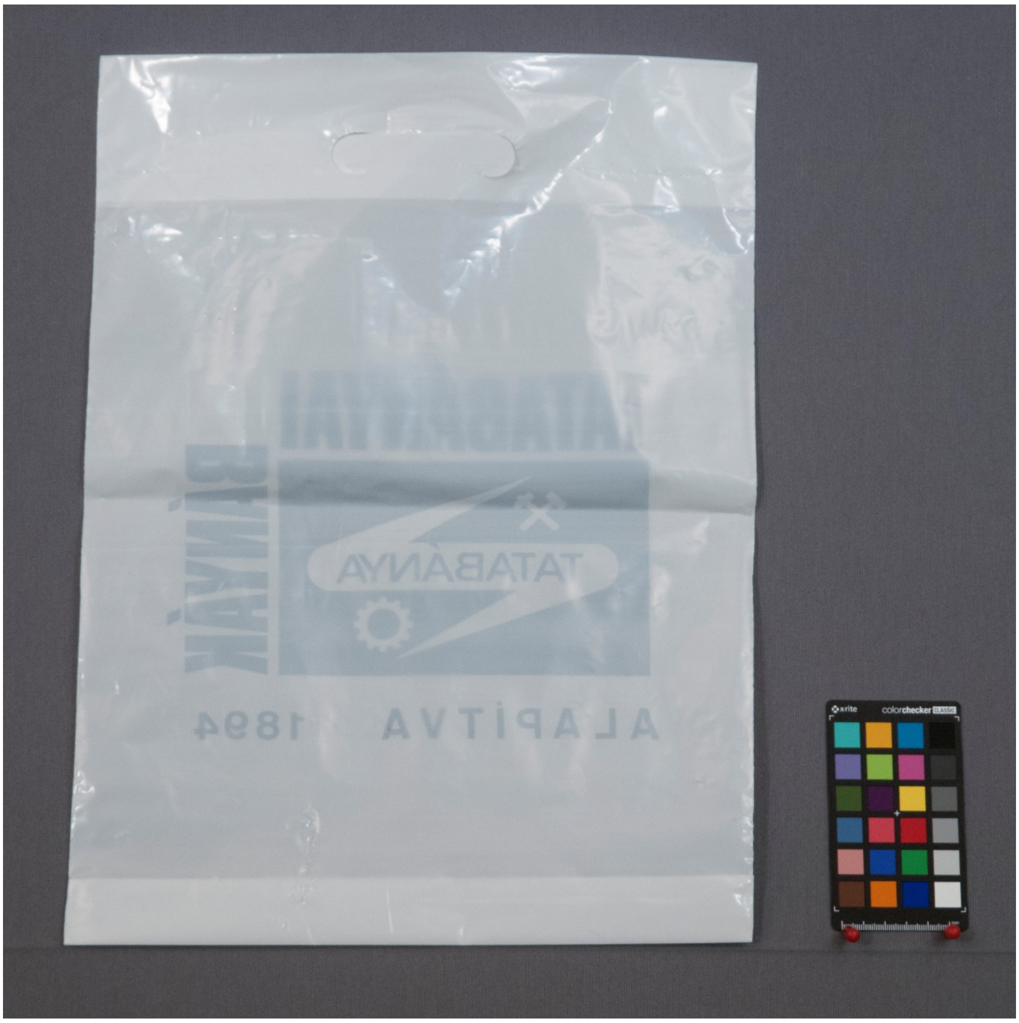

Figure S52. White polyethylene plastic bag with kink fold.

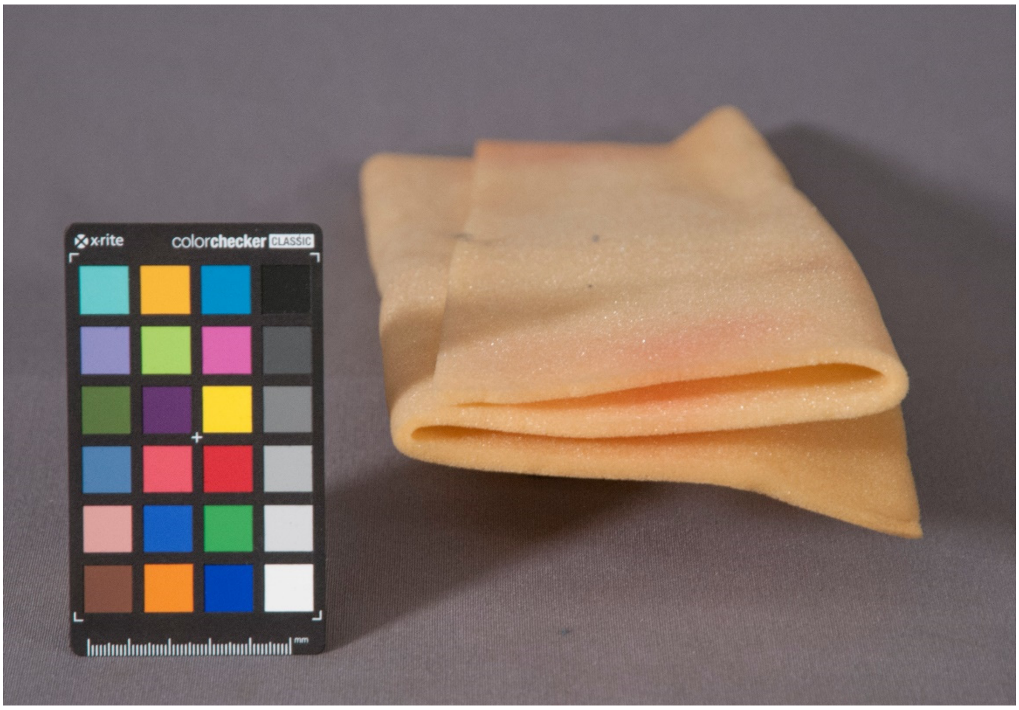

Figure S53. Folded polyurethane foam from a gadget case

**Hardening**	Loss of elasticity and plasticity, (partial) loss of the ability to recover its form and of the ability to change it without break after being submitted to stress.

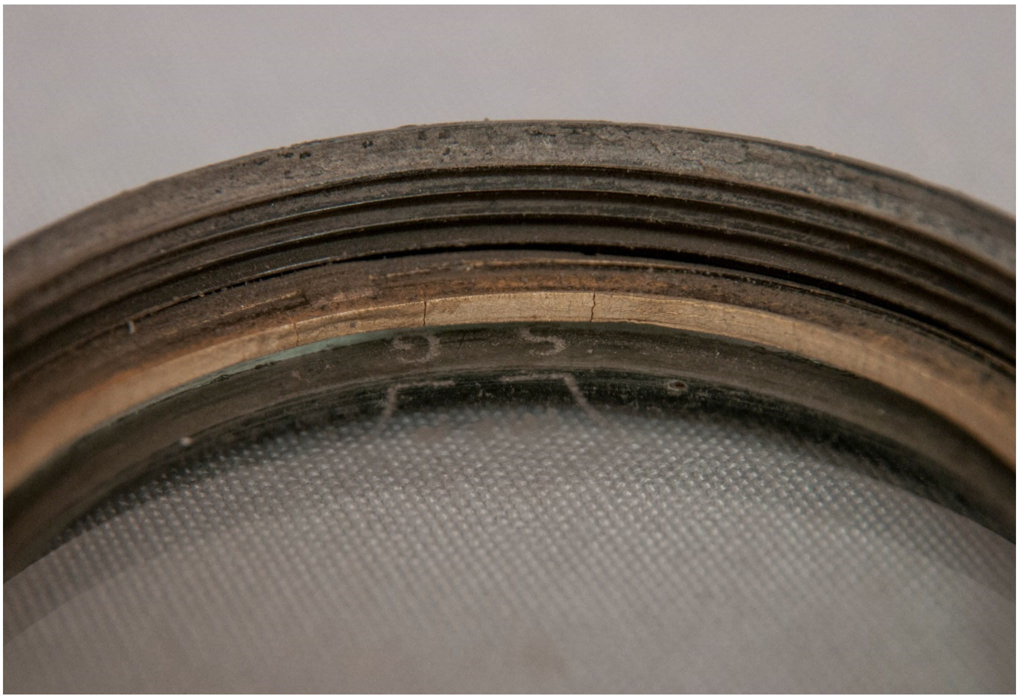

Figure S54. Hardened seal ring (styrene-based rubber) of a miner's lamp.

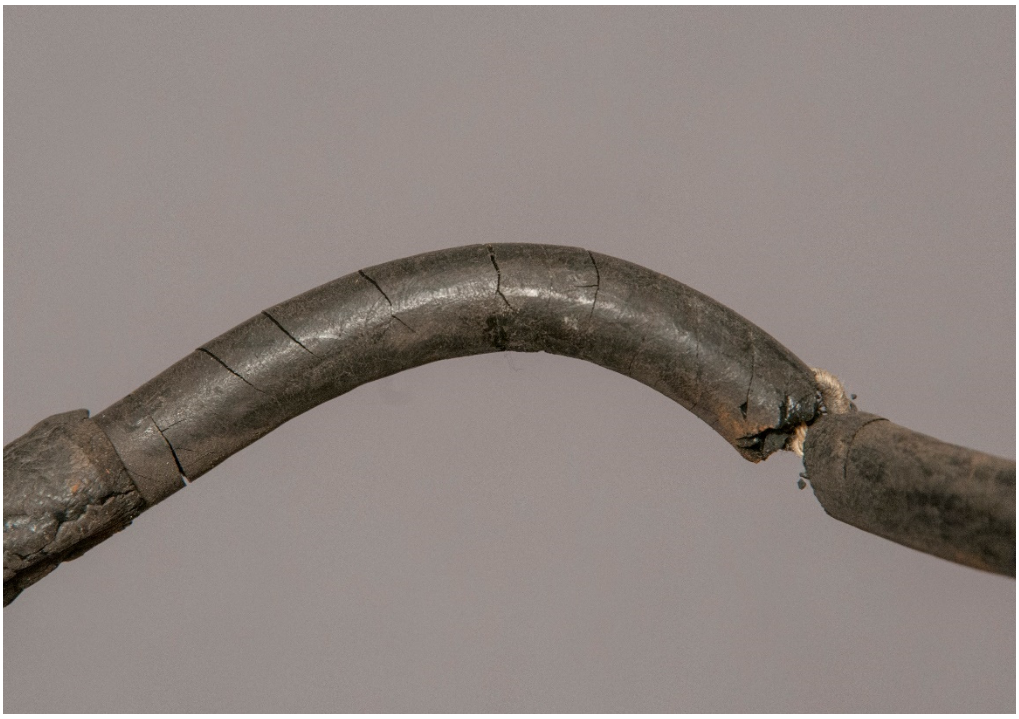

Figure S55. Hardening led to brittleness, cracks and breaks in a rubber-made electric cable sleeve.

**Loose**	Partial detachment of a constituting part of an object, with risk of breakage or loss of material.

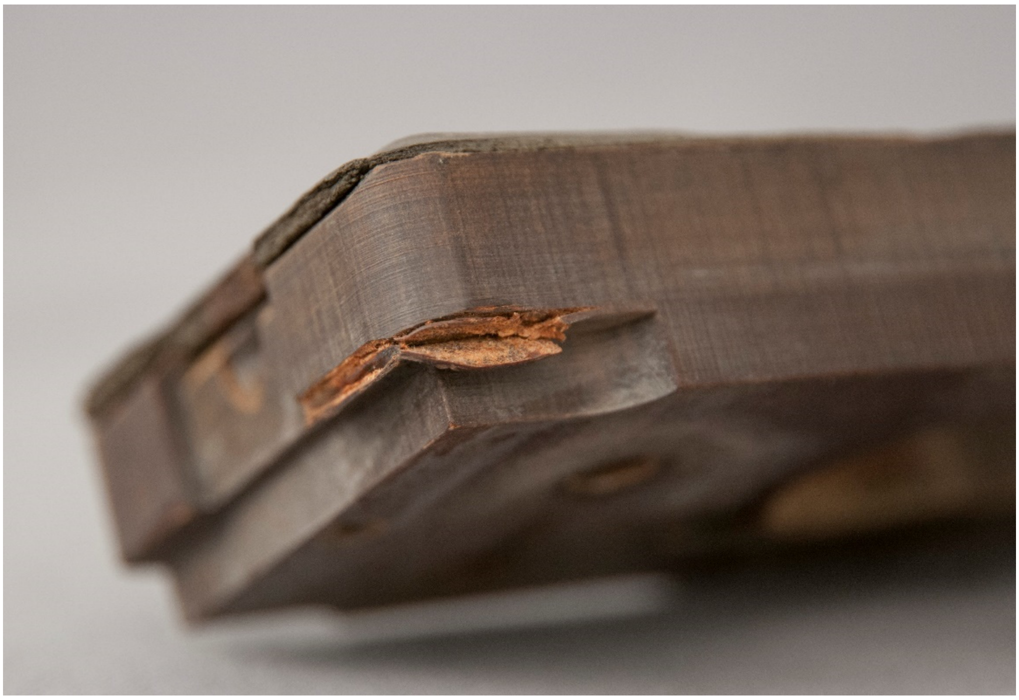

Figure S56. Isolation block from a miner's battery lamp with a loose part at the corner.

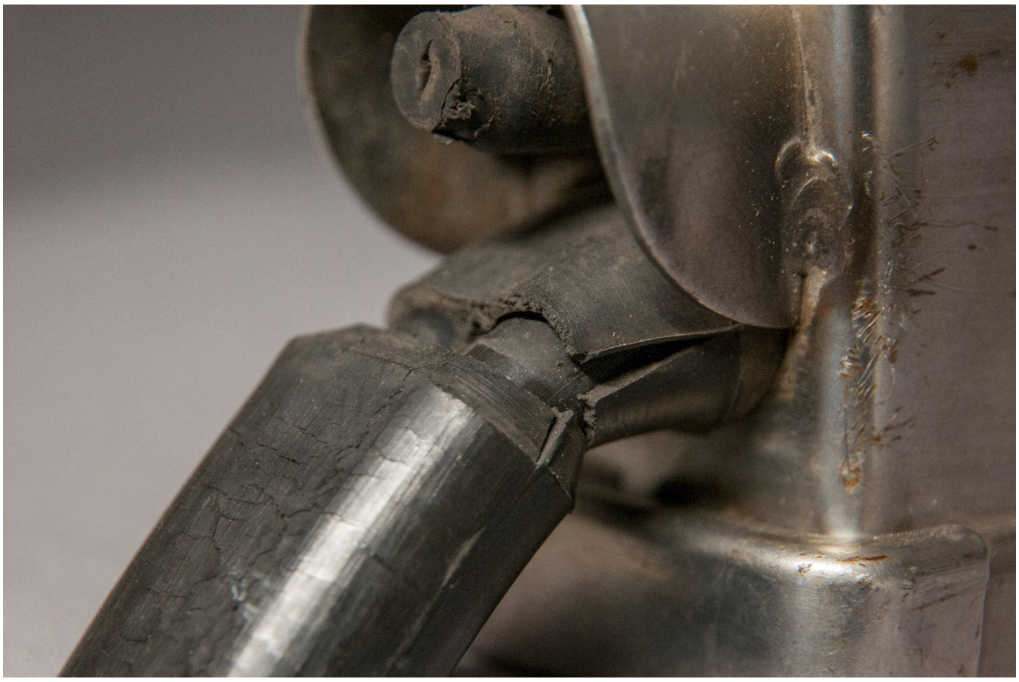

Figure S57. Cracked and loose bend protection of the cable from a miner's lamp.

**Loss of material (other)**	Loss of a constituting part of an object, as caused by a different damage phenomenon than abrasion, chip, crumbling, flaking or scratch.

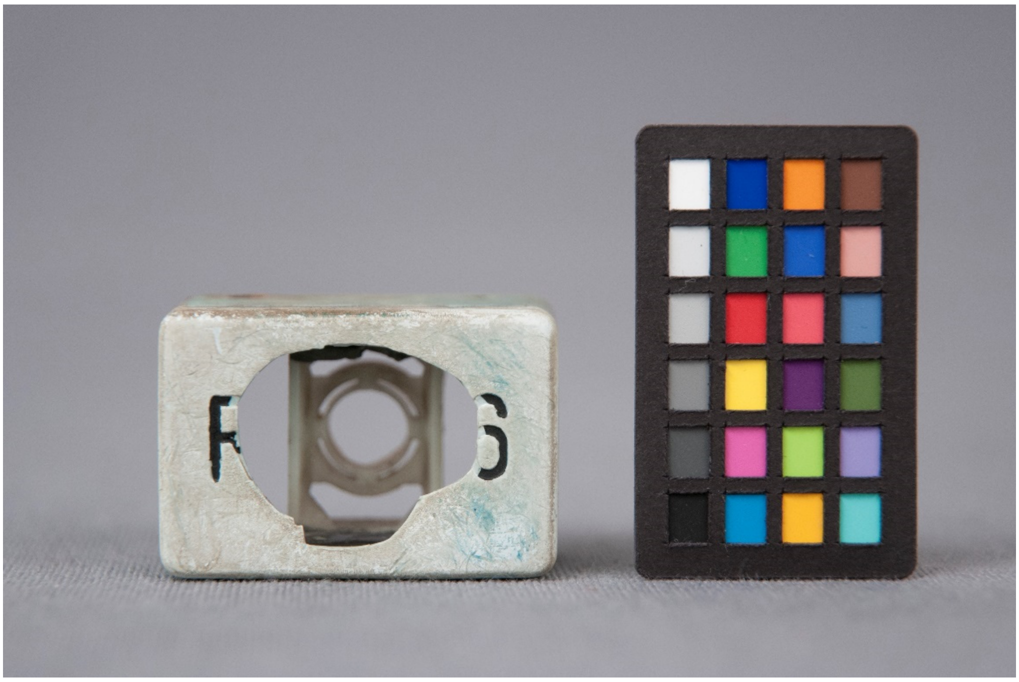

Figure S58. Hole in the button (polyoxymethylene) of a mine control station with material loss.

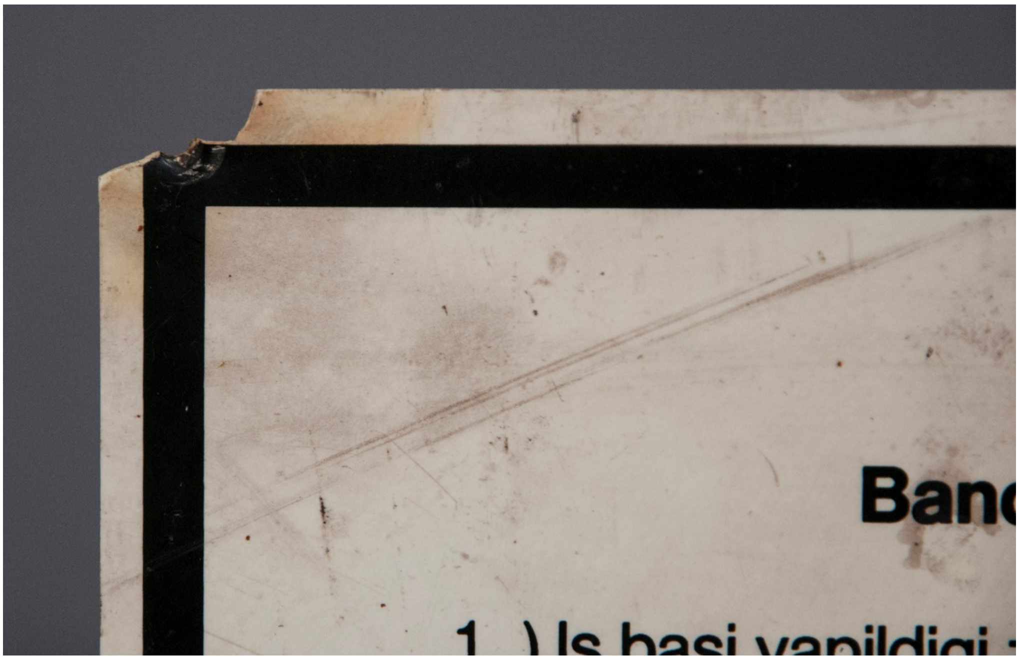

Figure S59. Lost corner part of an information board (polystyrene).

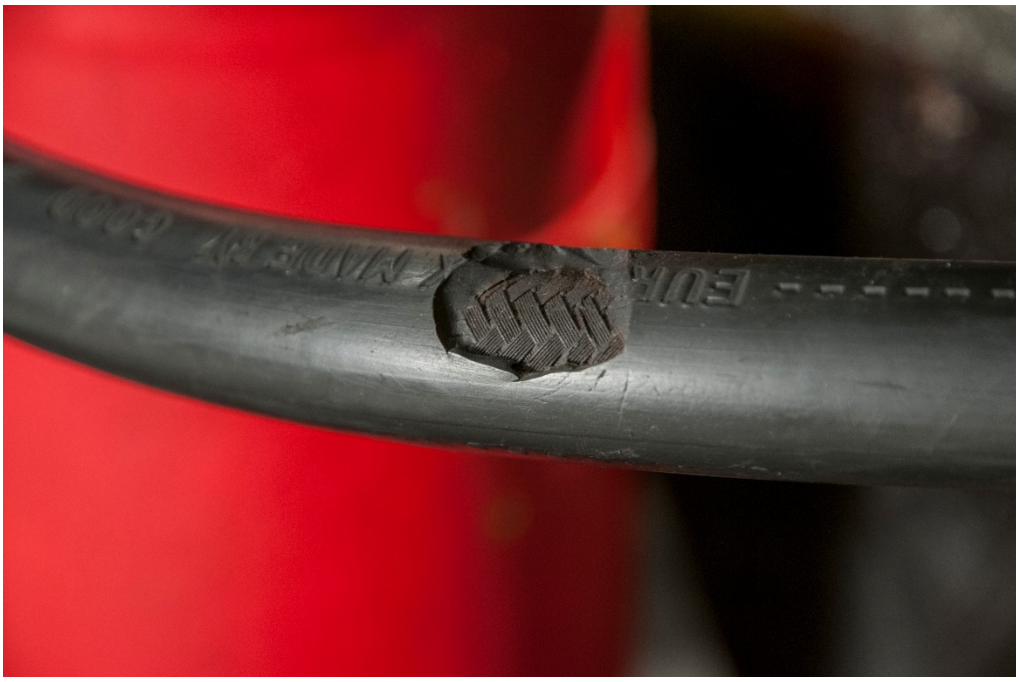

Figure S60. Loss of material in the sheath of a hydraulic hose.

**Loss of transparency**	Increase of the opacity of a transparent or translucent object.

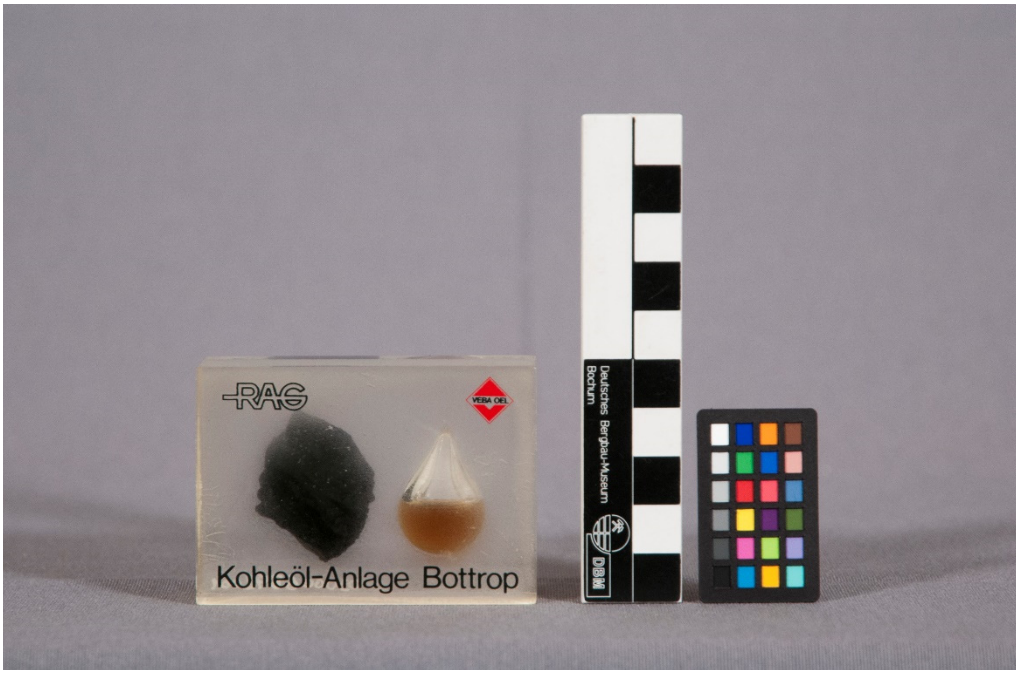

Figure S61. Transparency loss in the casting resin block of a paperweight (polymethyl methacrylate).

**Naphthalene smell**	Aromatic odour of the organic substance naphthalene, reminding of old, 20th century mothballs.

**Overpaint**	Colour layer that has been applied over an original surface.

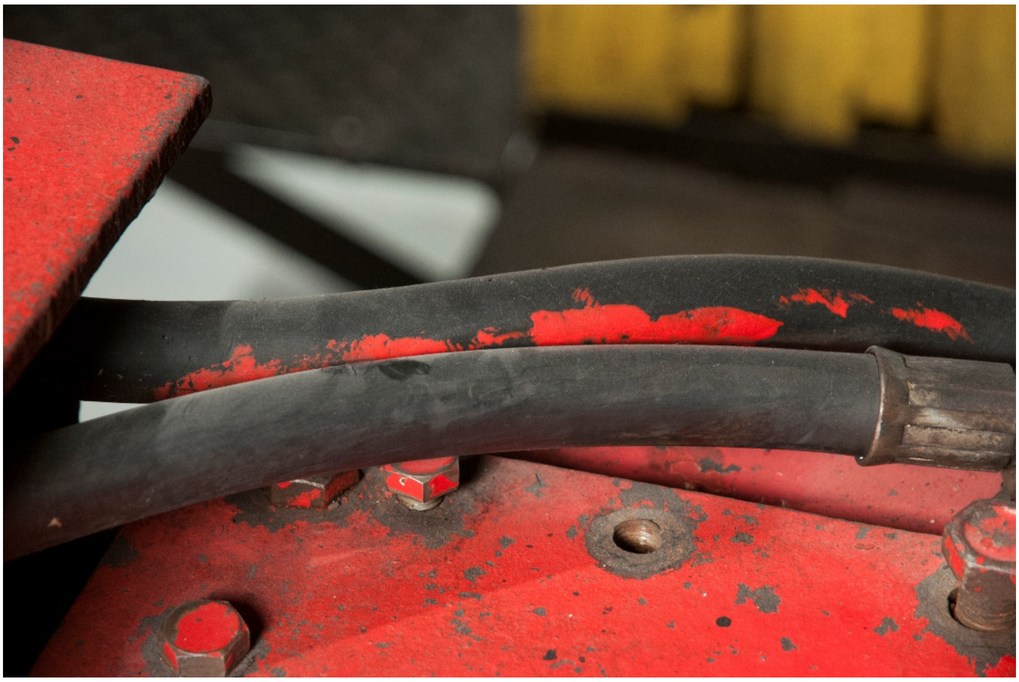

Figure S62. Red paint on the sheath of a hydraulic hose.

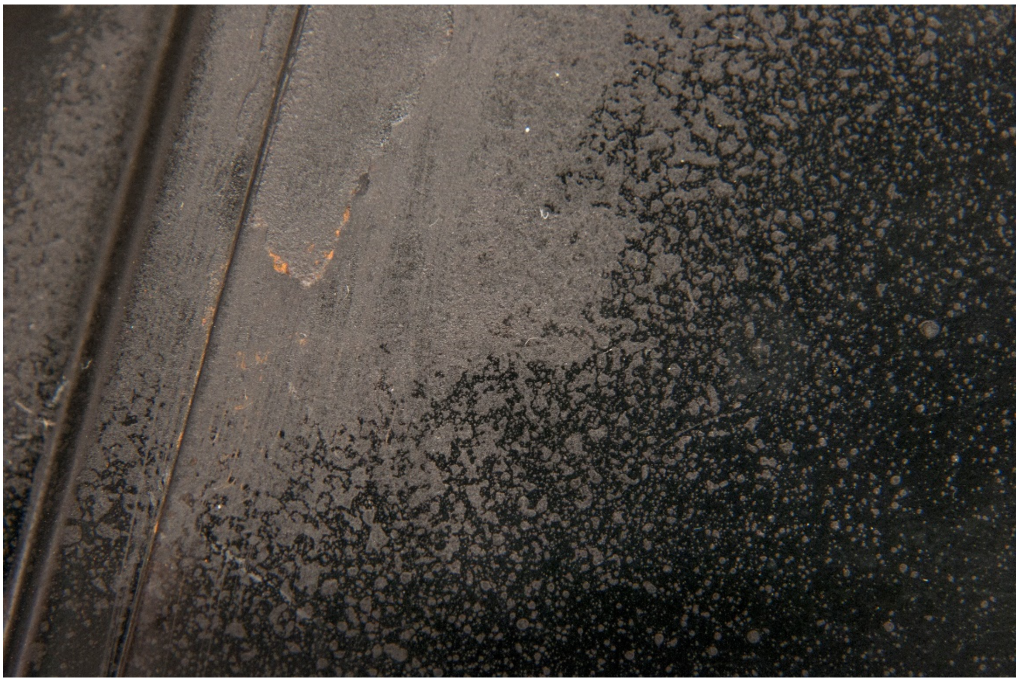

Figure S63. Grey repair of a damaged area in a black coating by use of spray paint.

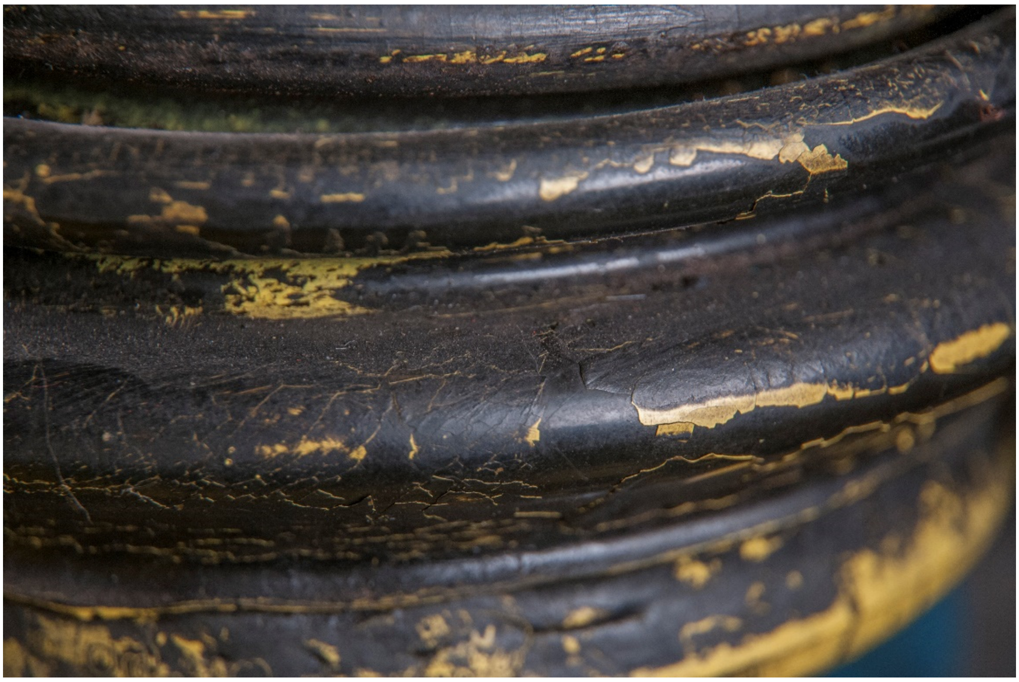

Figure S64. Yellow and black paint layers on a black gearshift bag.

**Peeling**	Detachment of a relatively large coating, foil or other layer from the underlying surface, sometimes accompanied by curling of the detached part; delamination.

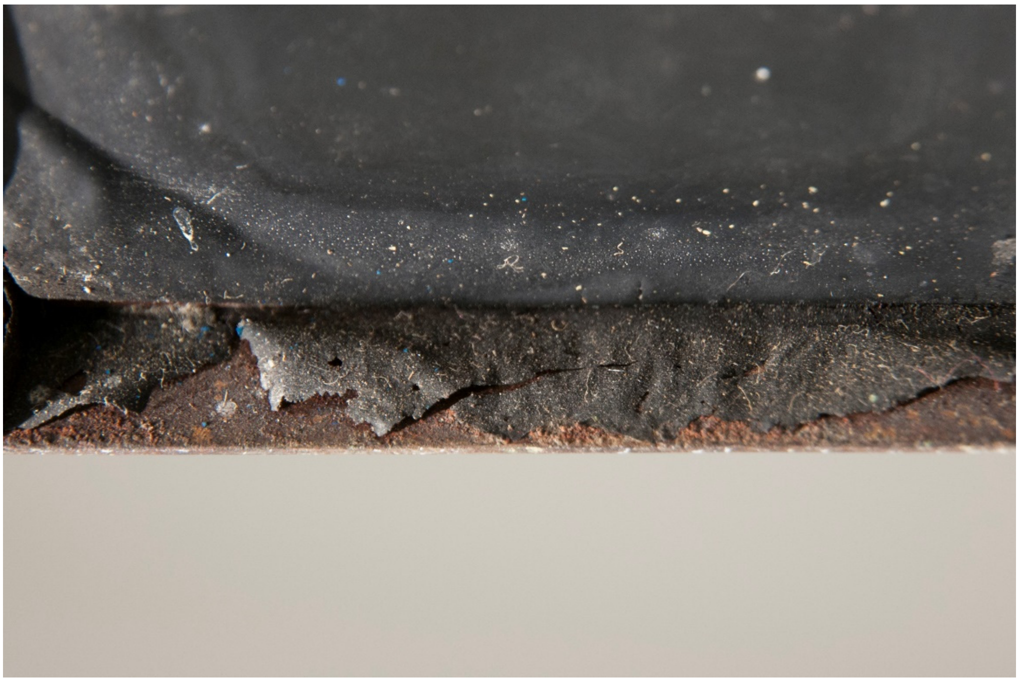

Figure S65. Black alkyd coating peels off from the metal case of a measuring device; with concomitant corrosion.

**Pox**	Small protrusions or elevations with the form of a truncated pyramid with round edges, which may show a different form, colour or texture from the surrounding material.

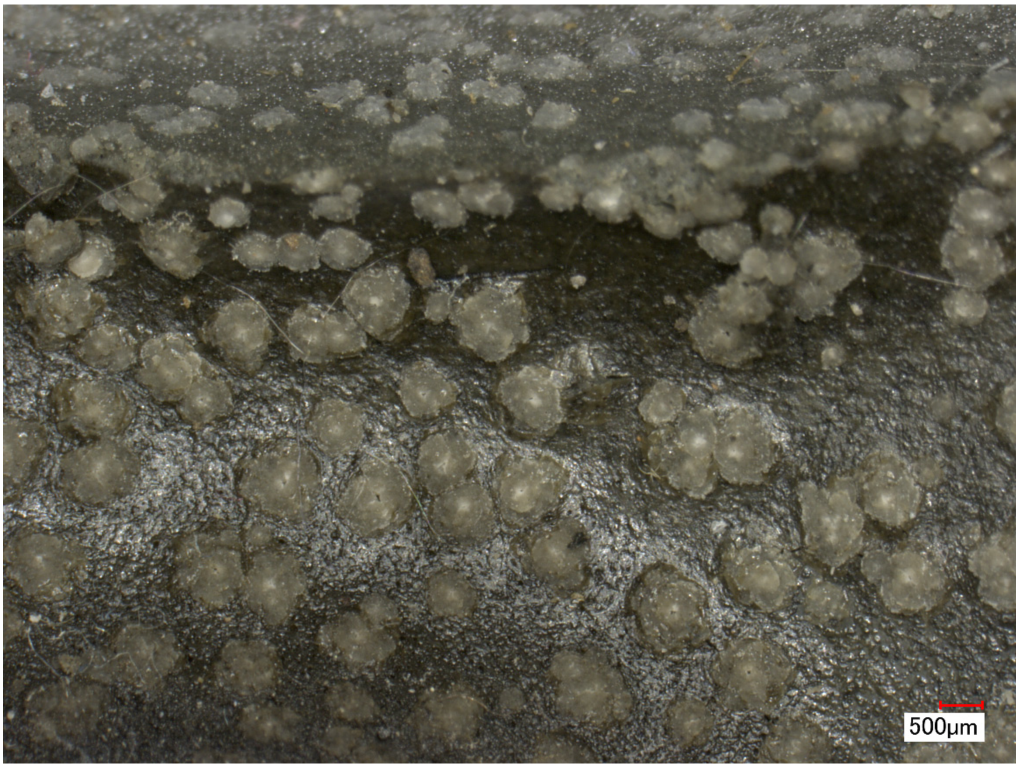

Figure S66. Pox in the surface of polyester urethane sole of miner's shoes (picture: J. Köppen).

**Rubber smell**	A typical rubber smell can be noticed in tyres or cheap rubber products.

**Scratch**	Rather linear, occasional or isolated thin incision(s) or scrape(s) on the surface, regardless of its depth; extensive accumulation of scratches in a given direction is rather an <abrasion>.

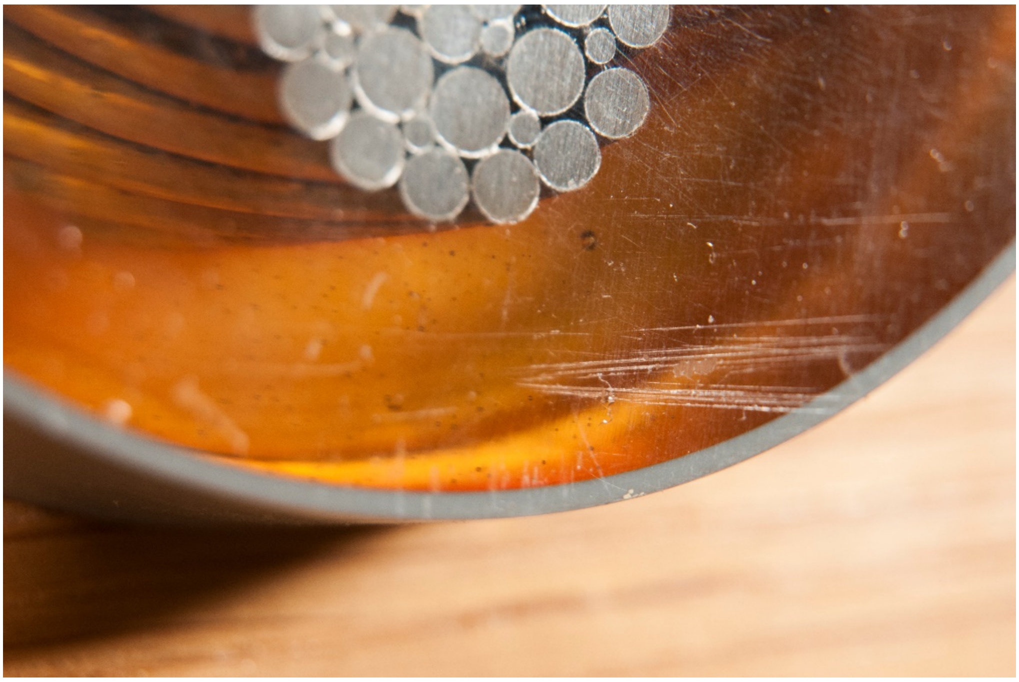

Figure S67. Scratches in the surface of cellulose nitrate varnish over cast resin on a display board.

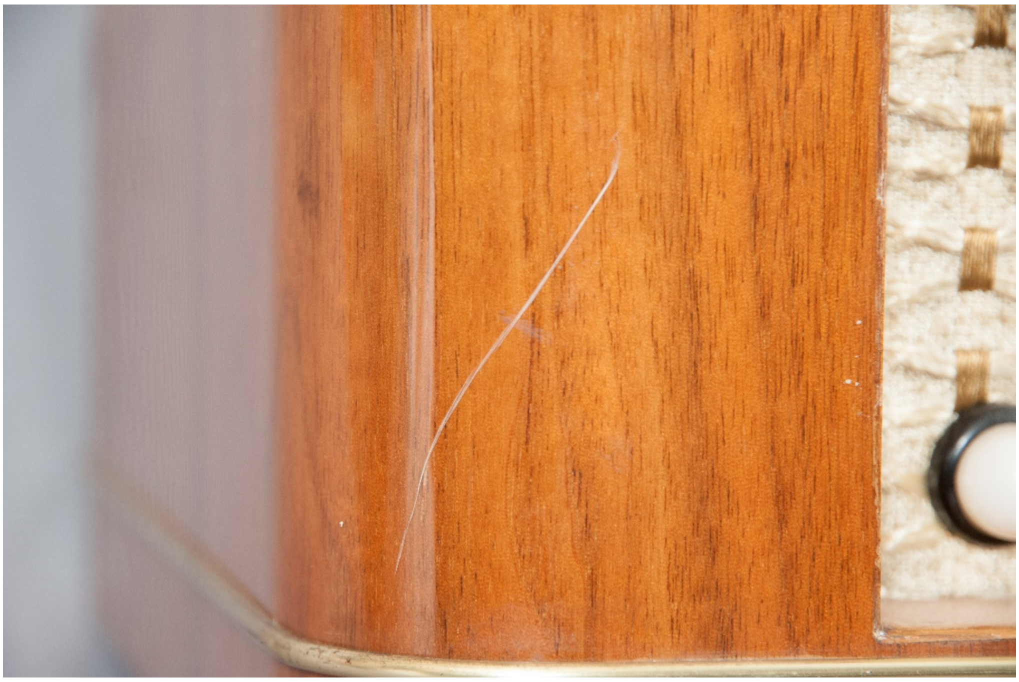

Figure S68. Single scratch in the transparent alkyd coating of a message receiver.

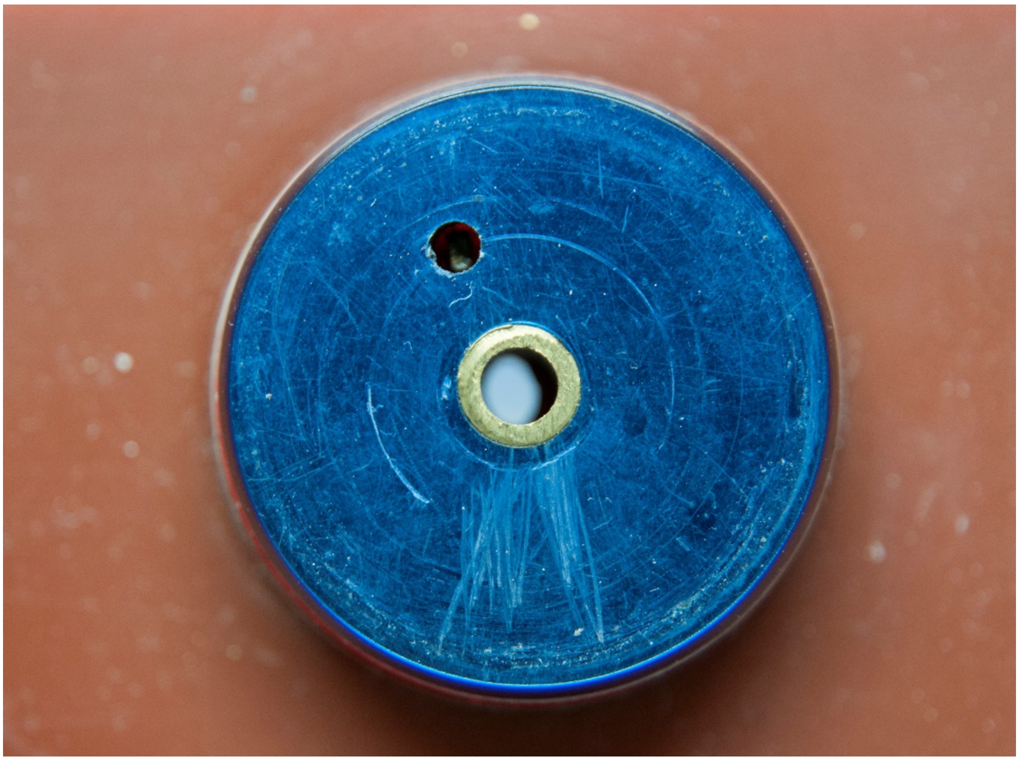

Figure S69. Scratched polymethyl methacrylate surface; also signs of damage by radial abrasion present.

**Shrinkage**	Dimensional reduction of the volume of a material; it may appear together with cracks and breakage.

**Smeary**	Oily or fatty residues, typically as a greasy film but not necessarily, coming from a known, external source (e.g. lamp oil); increased glossiness possible.

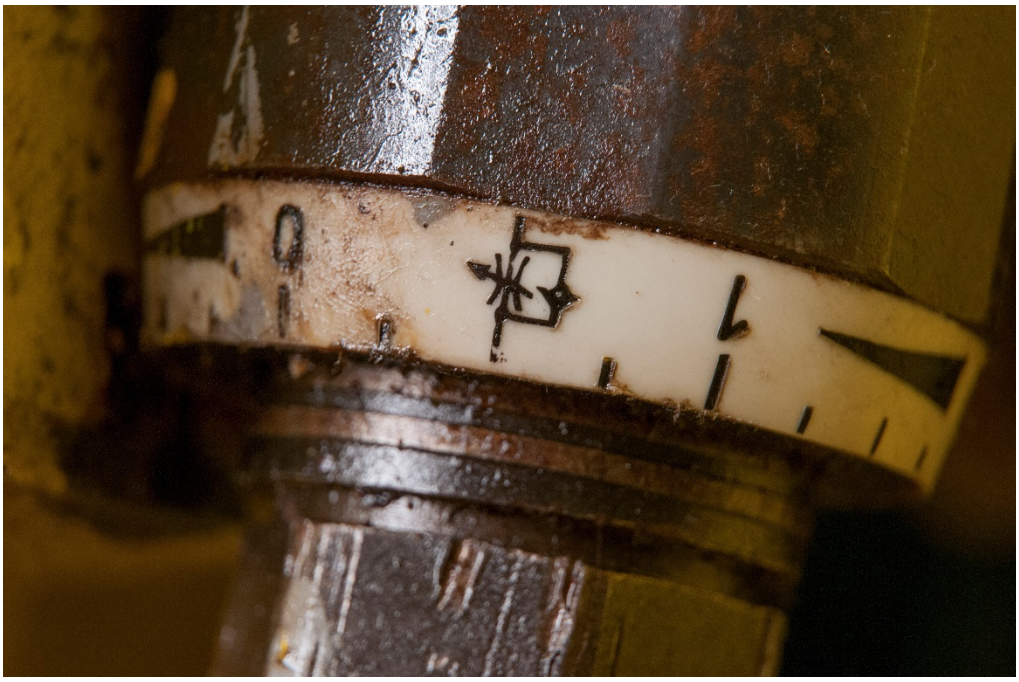

Figure S70. Oily deposits with increased gloss at the scaling of a hydraulic system.

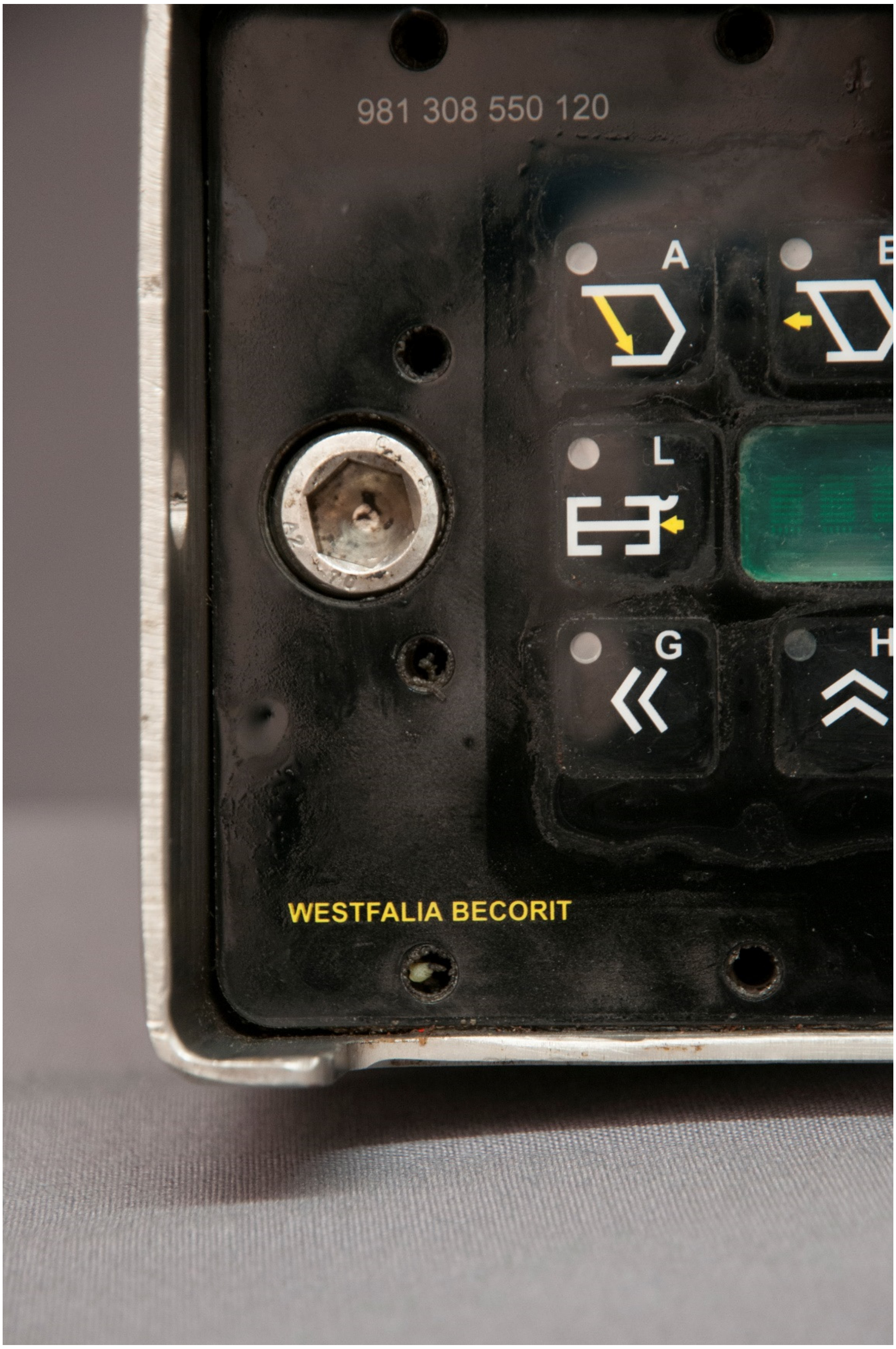

Figure S71. Fatty deposit on the operation field (polycarbonate) of a control unit, best visible over the word 'WESTFALIA'.

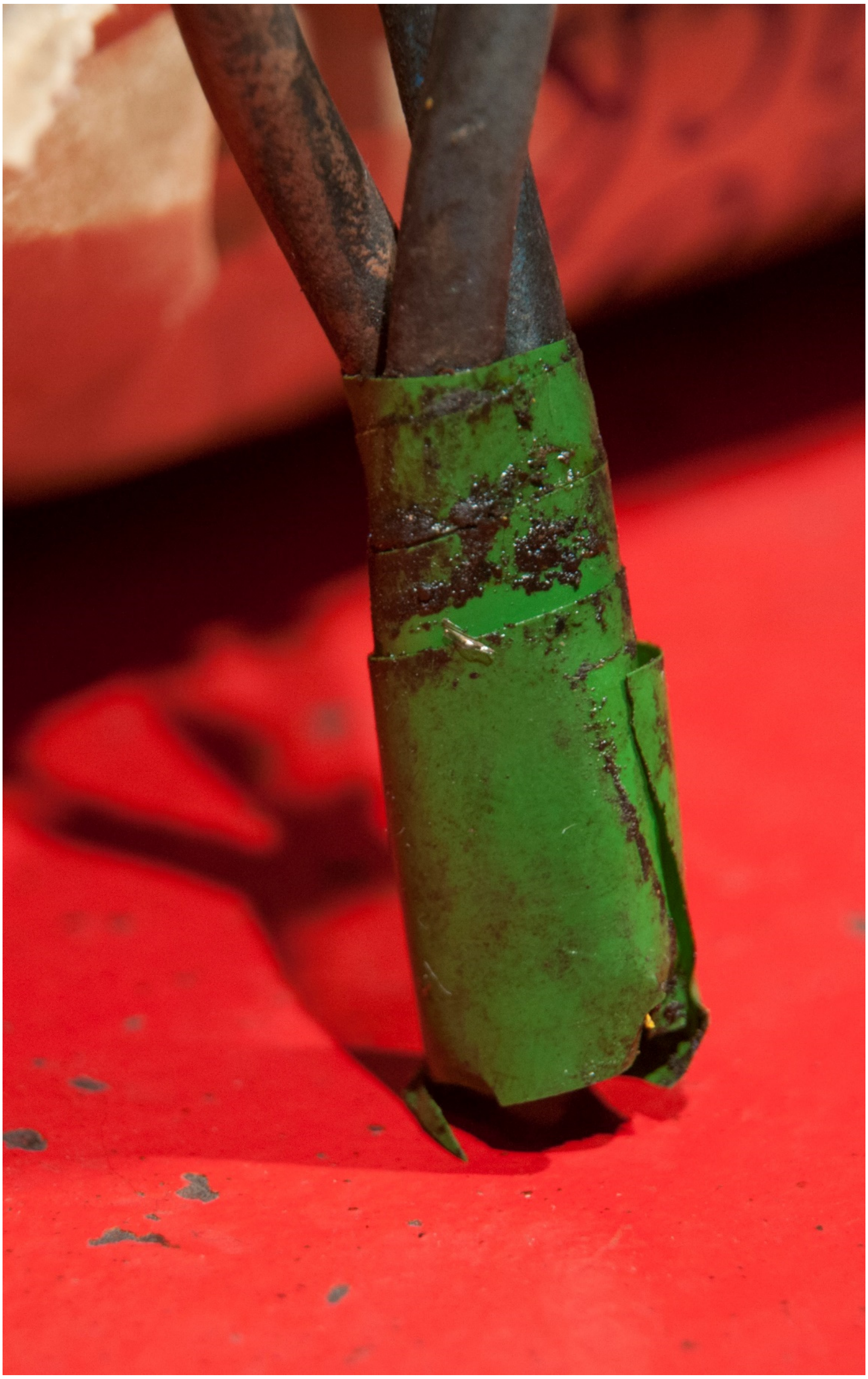

Figure S72. Smeary residue and sticky adhesive of green electric tape (plasticised polyvinyl chloride) at cable contacts.

**Softening**	Loss of hardness or firmness of the material, in extreme cases up to the point of liquefaction, which may have happened at some point in the material life. A (re)solidification may have taken place meanwhile, but the loss of original form, surface texture, glossiness or other changes hint towards a previous softening.

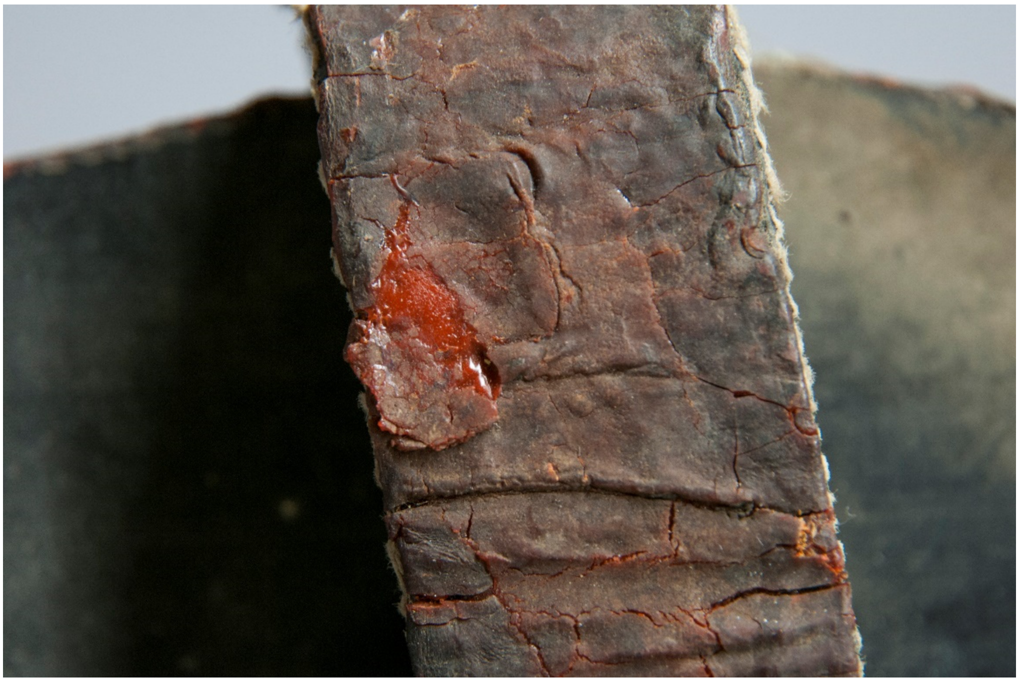

Figure S73. Softened and resolidified part of the polyisoprene-based belt from a bathing slipper from the 1950s.

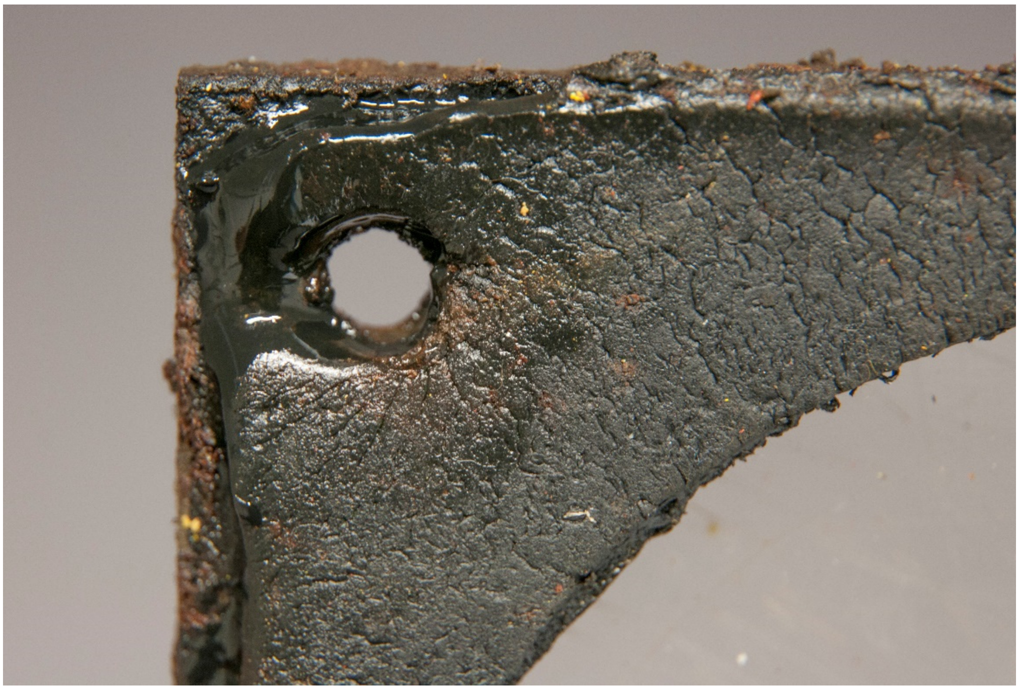

Figure S74. Liquefaction of a foam (probably EPDM-based with a further, styrene-based, polymeric component) in a pressure gauge.

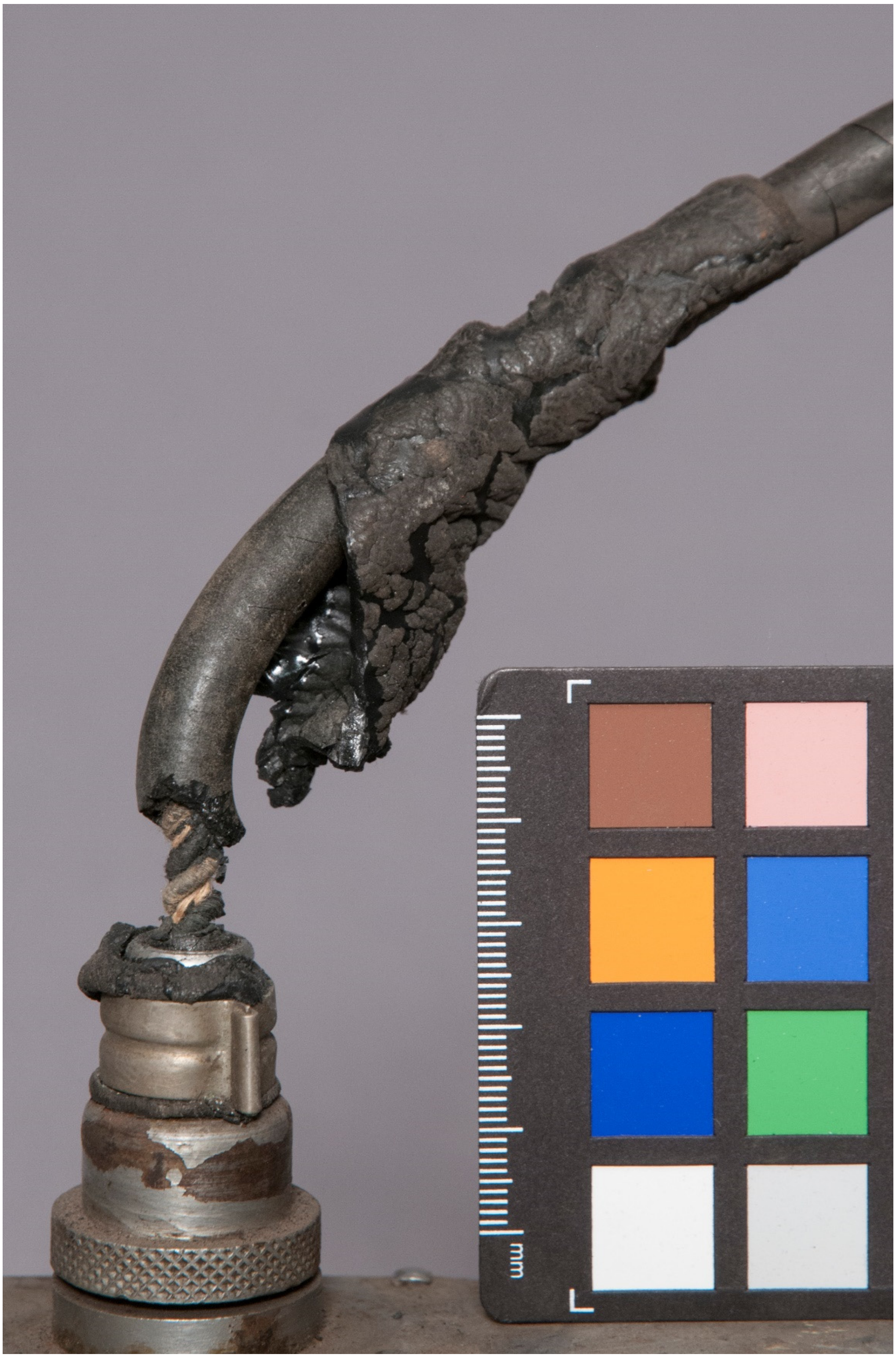

Figure S75. Softened and resolidified rubber-made bend protection at the electric cable of a miner's lamp from the 1920s.

**Stain**	Transfer of foreign material from an external object to the surface of study through rubbing off, spill or other process, causing changes in colour, texture and / or glossiness in a localised area. E.g. dash of colour.

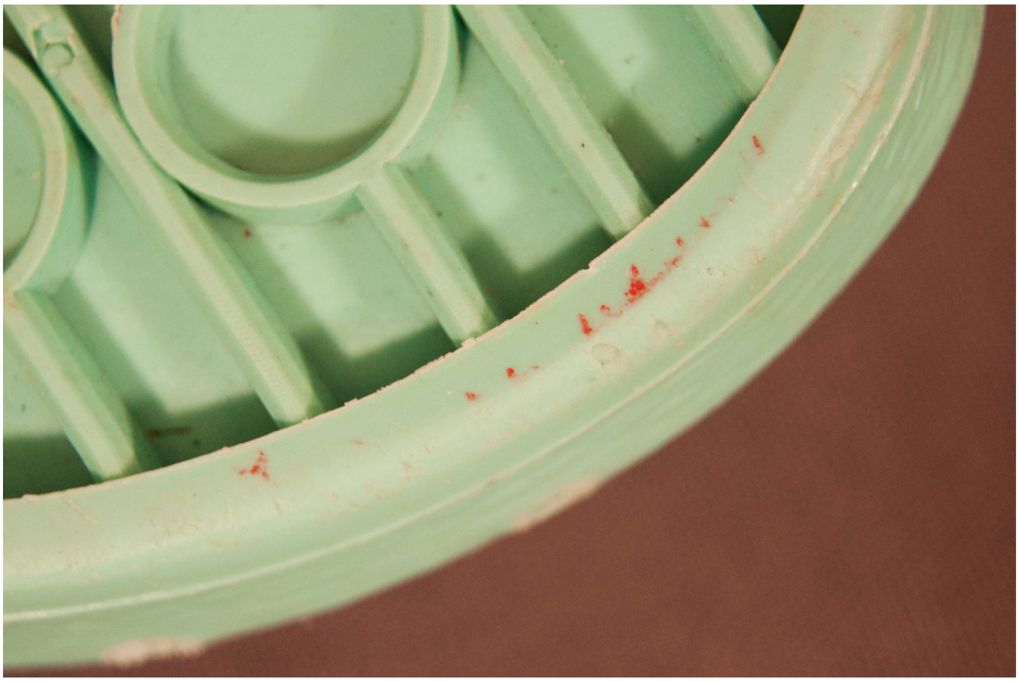

Figure S76. Rubbed off red paint on the sole part of light green bathing slippers.

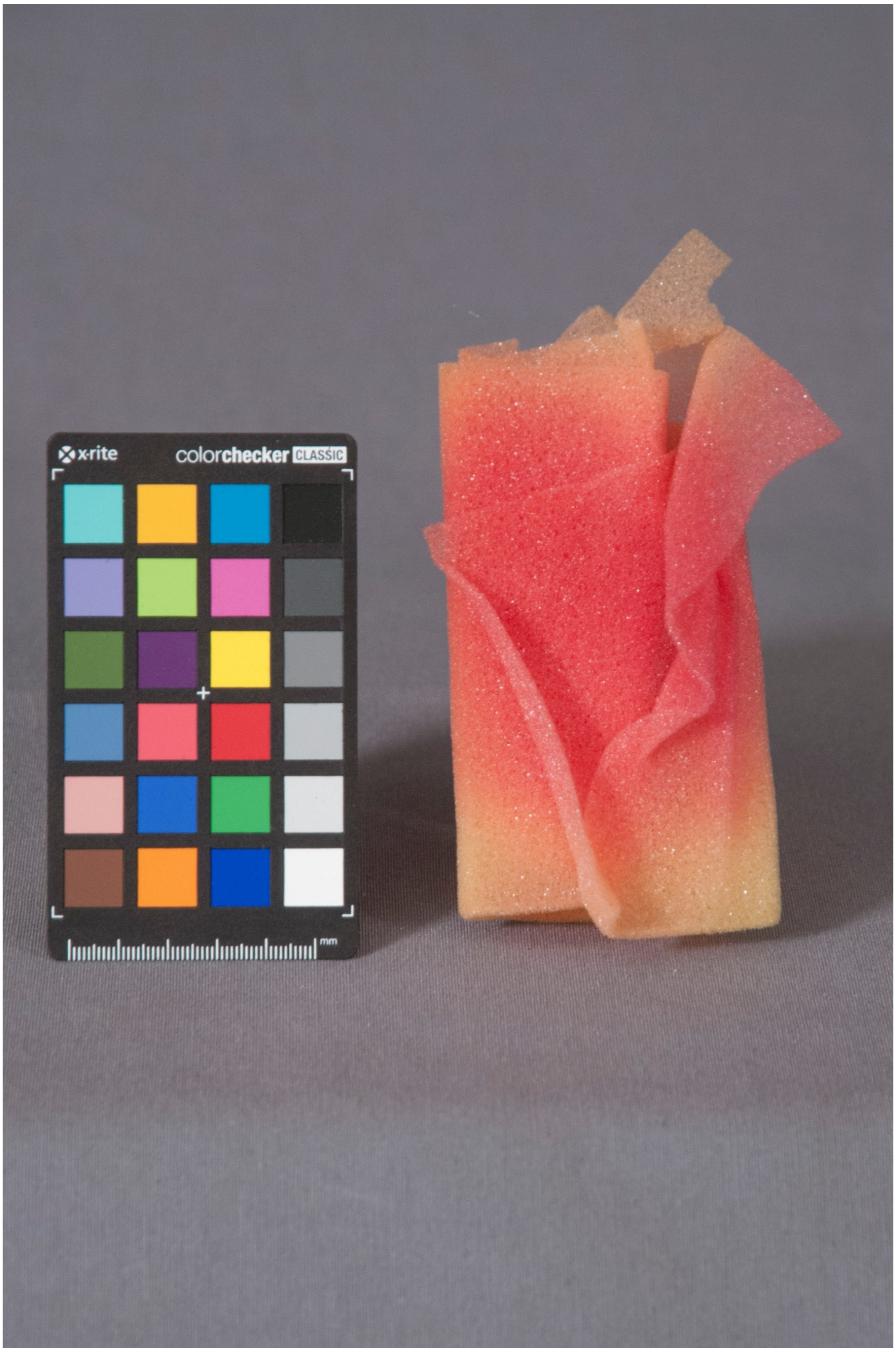

Figure S77. Polyurethane foam dyed by red ink.

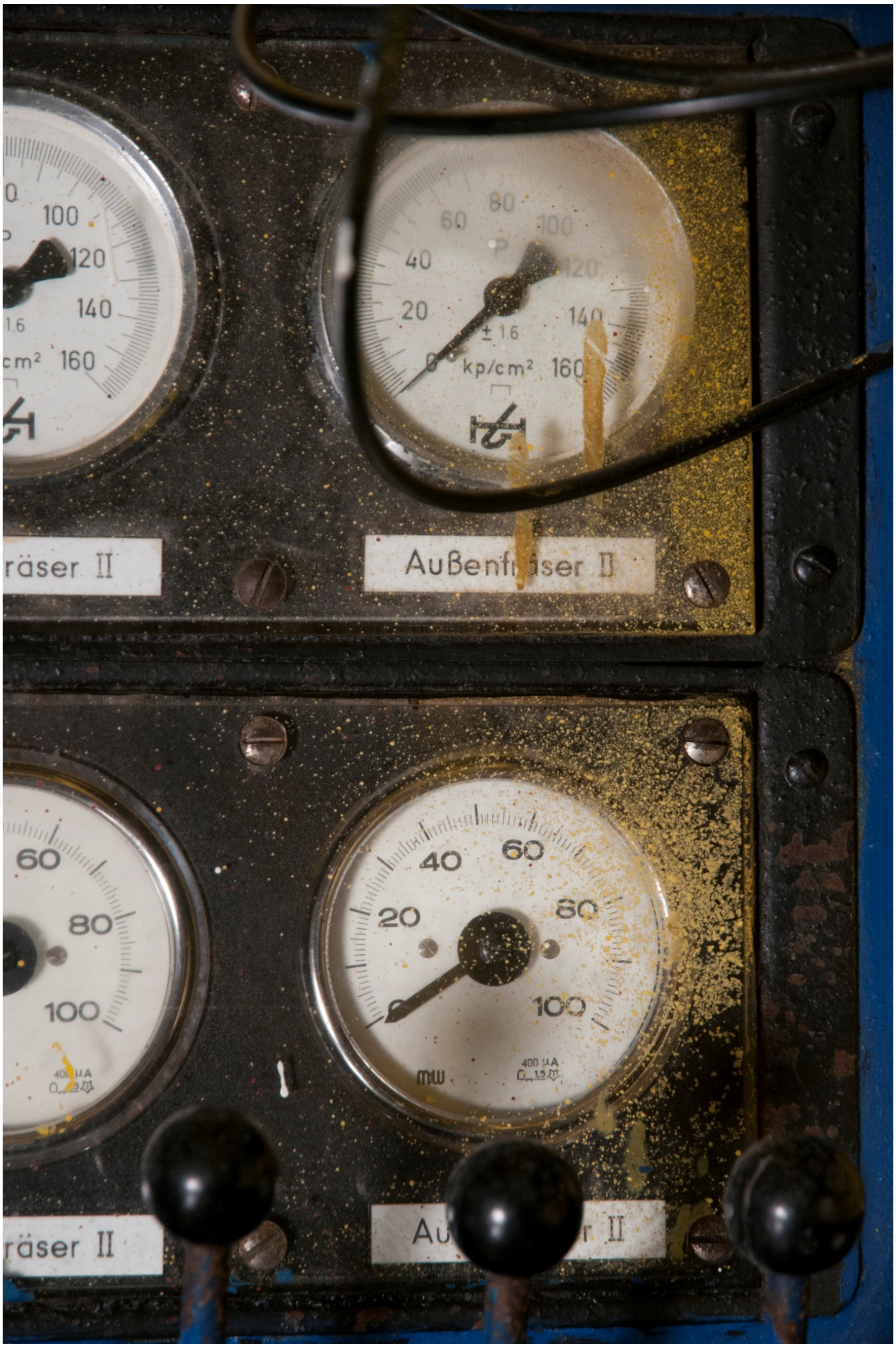

Figure S78. Yellow colour splash on the transparent display window of pressure gauges.

**Sticky**	Adhesive or gluey character of a surface; behaviour of a surface through which it stays attached to other surfaces that it touches.

**Sweating**	Liquid phase occurring either in the form of drops (moisture) or of a film, of origin unknown; increased glossiness possible.

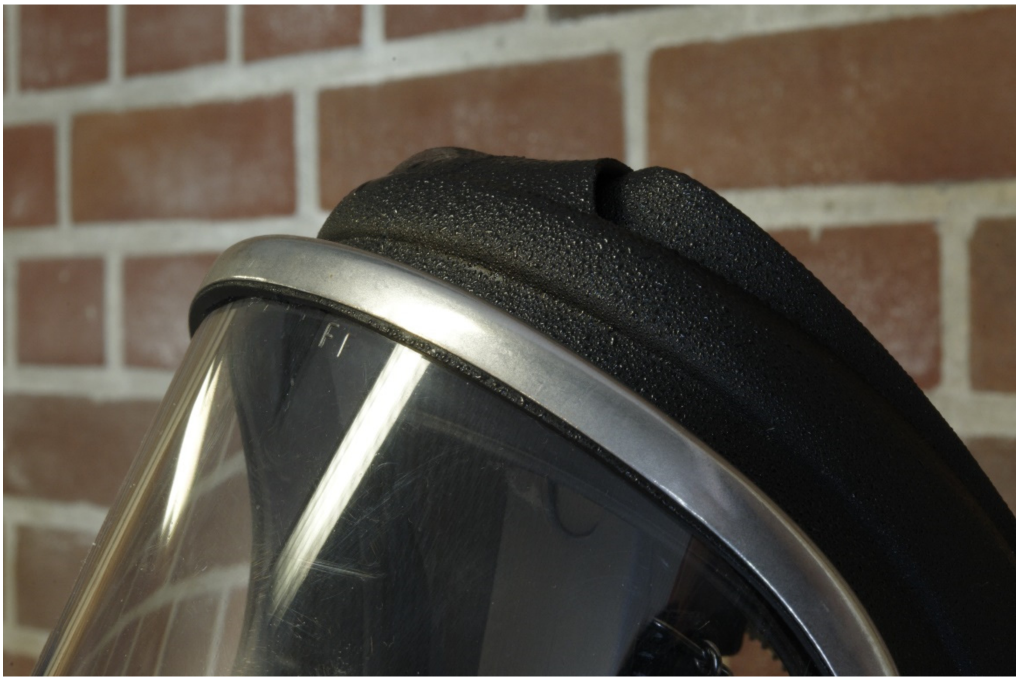

Figure S79. Droplets on the body of a gas mask (isoprene-based rubber), building up sweating.

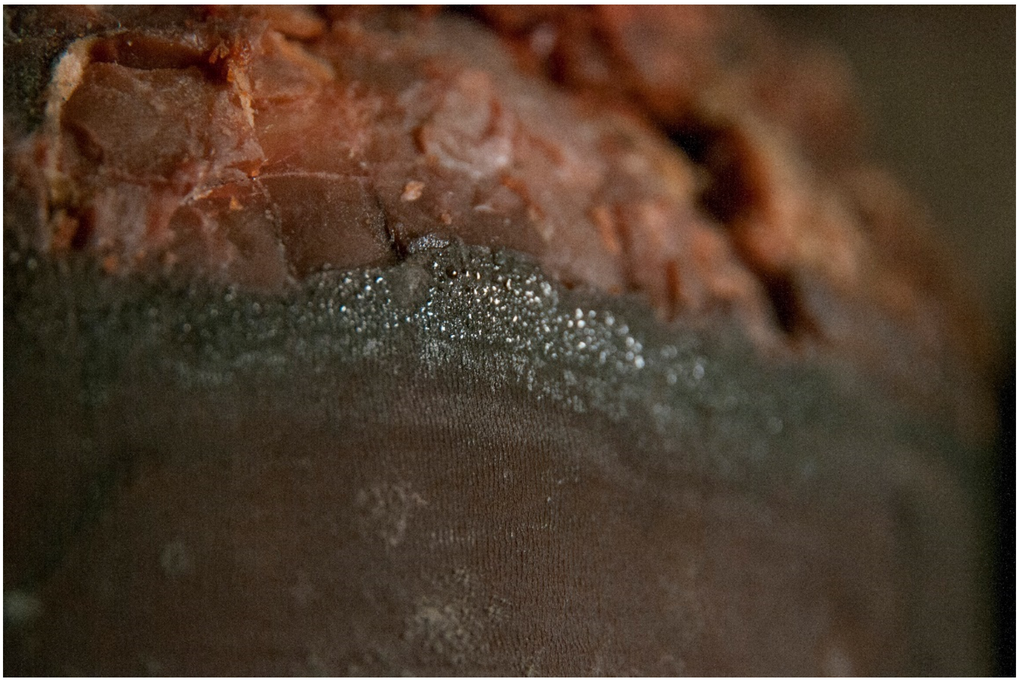

Figure S80. Sweating at a friction drive (wheel) [polyester urethane] of a monorail system.

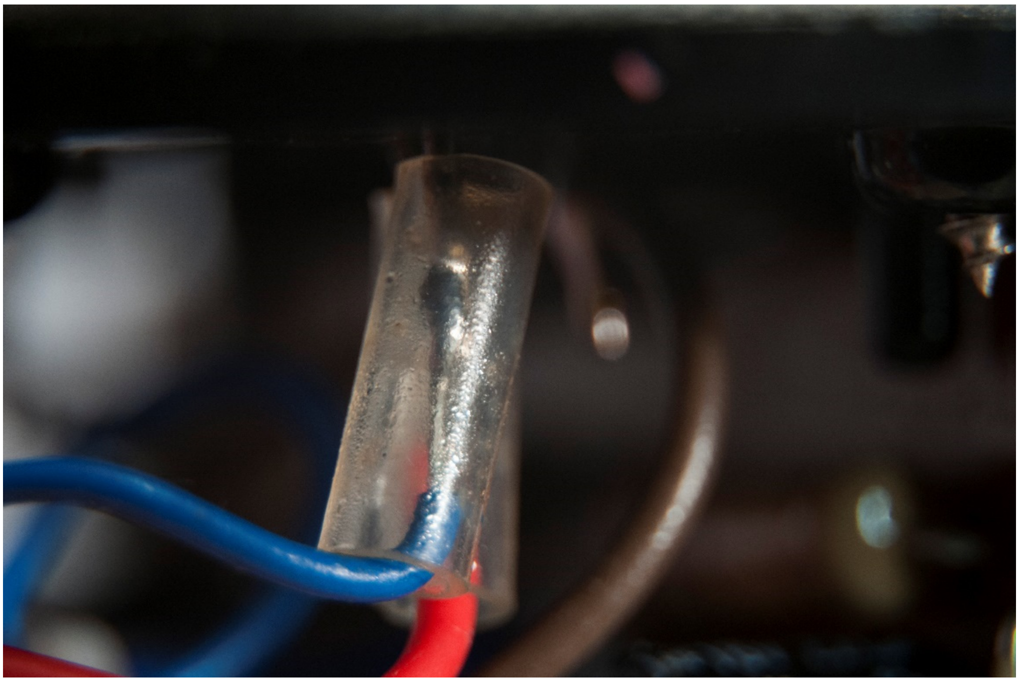

Figure S81. Sweating in an isolating hose (plasticised polyvinyl chloride) for cable contacts.

**Vinegar smell**	Typical smell of vinegar coming from acetic acid appearing as a degradation product.

**Warping**	Three-dimensional form change appearing as concave and / or convex distortion or twisting, sometimes in the form of a wave, so that the material / object surface is no longer flat.

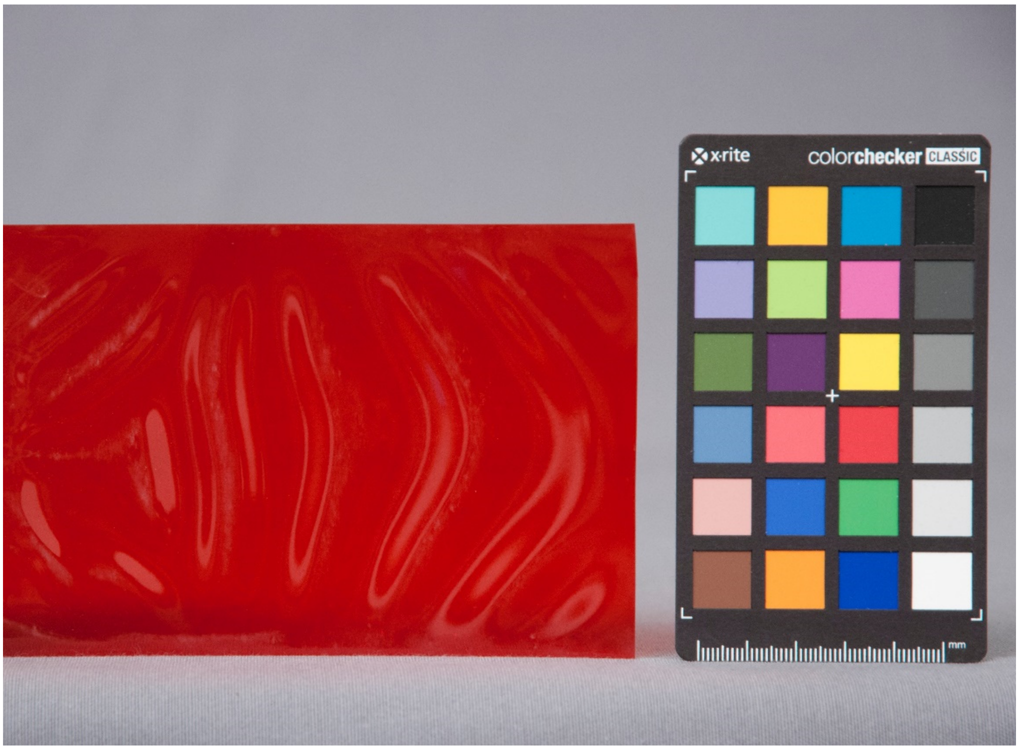

Figure S82. Wave-shaped warping of a red translucent foil (cellulose acetate).

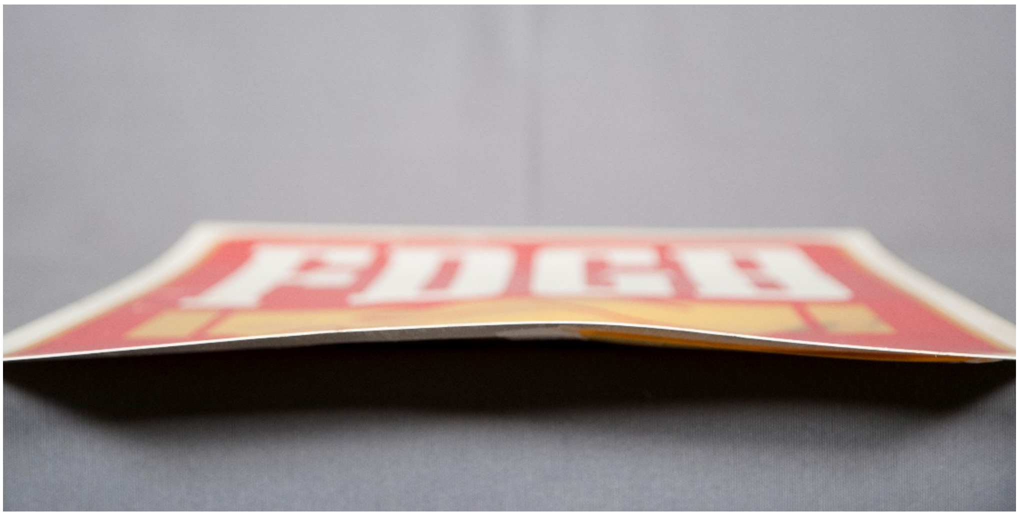

Figure S83. Warped information sign (unplasticised polyvinyl chloride).

**Yellowing**	Colour change to a yellow tinge / hue / taint / cast, which can sometimes be darker (red / brown). Easily visible in clear or lightly coloured objects.

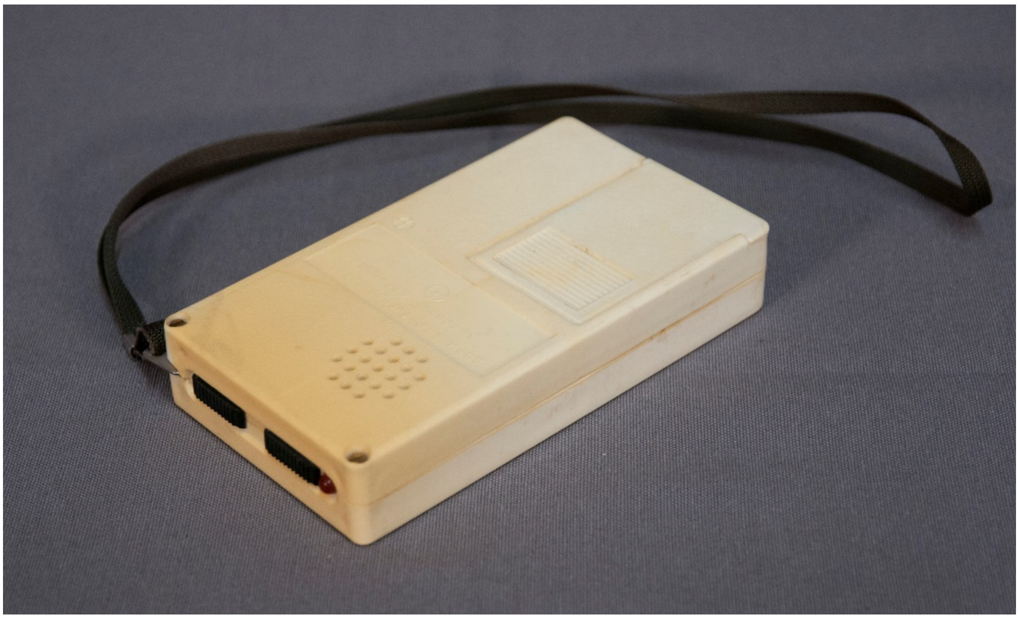

Figure S84. Partly yellowed case of a radiation dosimeter, made of acrylonitrile butadiene styrene.

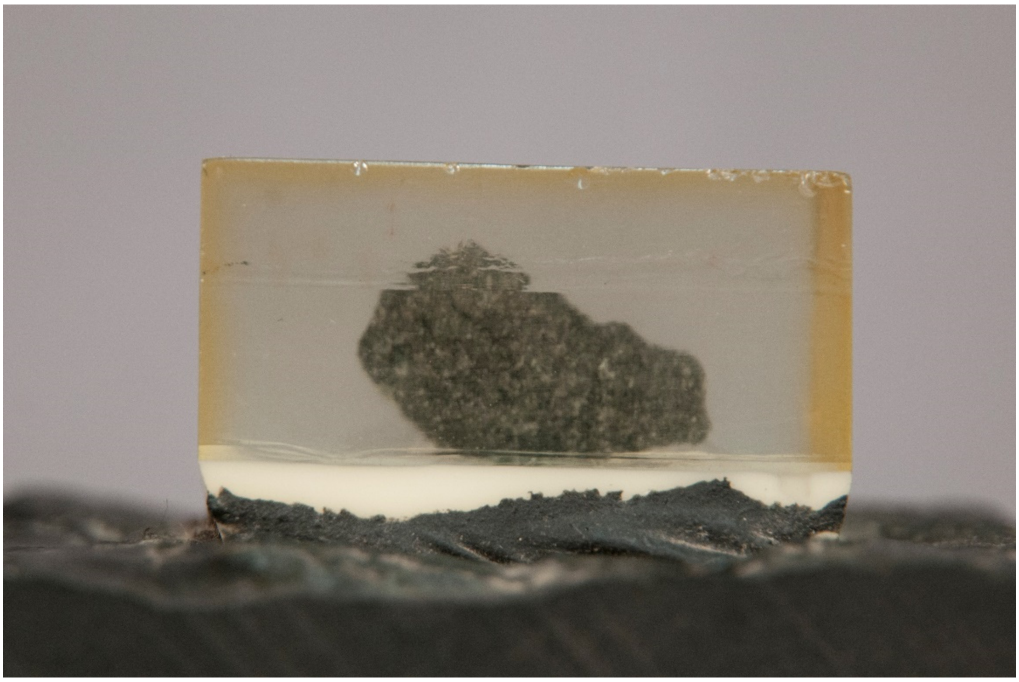

Figure S85. Yellowed transparent casting resin block (unsaturated polyester resin) of a paperweight from the 1960s.

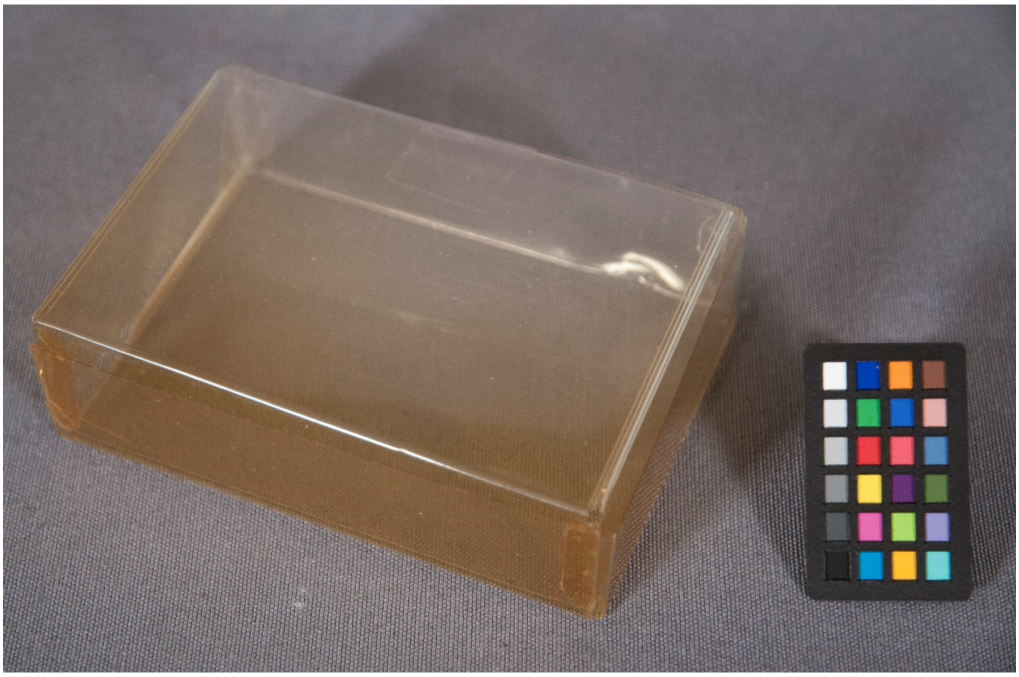

Figure S86. Yellowing of the transparent wrapping (unplasticised polyvinyl chloride) of a souvenir.
